# Analyzing pre-service biology teachers’ intention to teach evolution using the theory of planned behavior

**DOI:** 10.1186/s12052-022-00175-1

**Published:** 2022-11-18

**Authors:** Helena Aptyka, Jörg Großschedl

**Affiliations:** Institute for Biology Education, Faculty of Mathematics and Natural Sciences, University of Cologne, Herbert-Lewin-Straße 10, 50931 Cologne, Germany

**Keywords:** Evolution, Intention, Evolution education, Theory of planned behavior, Teacher education, Pre-service teacher, Structural equation model, Preference, Teaching, Biology education

## Abstract

**Background:**

Even though evolution is the overarching principle that connects all areas of biology, a significant proportion of pre-service teachers do not intend to teach evolution, minimize the teaching of evolution, or teach alternative ideas in biology classes. To prevent adverse teaching practices and promote effective pre-service teacher education, we aimed to identify and analyze variables that foster or hinder their behavioral intentions to teach evolution.

**Method:**

We adopted a behavioral psychology research perspective and developed a research model based on the theory of planned behavior to examine behavioral intentions for teaching evolution in biology classrooms. We extended the model with additional variables that have been delineated by teacher education research as essential determinants for the behavioral intention to teach evolution. We proposed several hypotheses suggesting that the attitude toward teaching evolution, subjective norms, perceived behavioral control, personal religious faith, perceived usefulness, and knowledge about evolution determine a person’s behavioral intention. We conducted a quantitative cross-sectional study in teacher education to test the hypotheses and surveyed *N* = 339 pre-service biology teachers using an online questionnaire. We analyzed the data using a two-stage structural equation model.

**Results:**

We were able to confirm all proposed hypotheses. The most important results revealed that pre-service teachers’ knowledge about and perceived usefulness of evolution are only moderately pronounced. Moreover, the subjective norm is a predictor not only of behavioral intention but also of the attitude toward teaching evolution. The variable of perceived behavior control partly moderates the relationship between knowledge about evolution and behavioral intention. Additionally, perceived usefulness is an important and marginally stronger predictor of a person’s attitude than personal religious faith.

**Conclusion:**

The extended model of the theory of planned behavior has highlighted the need for educational programs to increase knowledge about and the perceived usefulness of evolution even stronger. The findings delineated the effects of essential determinants on behavioral intentions and provided information about the necessary levers of teacher education.

**Supplementary Information:**

The online version contains supplementary material available at 10.1186/s12052-022-00175-1.

## Introduction

More than 150 years have passed since Darwin’s theory of evolution Darwin (1859) was published. It caused much resentment at that time, as the theory of evolution represented a dissent from the social worldviews. Nevertheless, this scientific theory explains the biodiversity of life and the origin and change of biological entities. Therefore, it revolutionized the conceptualization of biology. Since then, knowledge and understanding of evolution have been considered essential for the comprehension of and reflection on daily processes that underlie evolutionary concepts such as variation, individual fitness, and mutations (Council of Europe 2007; Darwin 1859; Nationale Akademie der Wissenschaften Leopoldina [Leopoldina] 2017). For example, evolution empowers students, non-professionals, and researchers to learn from the COVID-19 situation, weigh the risks of mutations, and promote medical research to effectively combat future viruses (Dillon 2016; Serpa et al. 2021; Smith 2010).

Despite the importance of evolution, a substantial proportion of pre-service and in-service teachers do not accept it as the currently most powerful scientific explanation for evolutive processes (e.g., Graf and Soran 2010; Kim and Nehm 2011). Additionally, studies showed that a substantial proportion of pre-service and in-service teachers did not intend to teach evolution in biology classrooms (e.g., Moore and Cotner 2009; Trani 2004) or aimed to teach creationism instead of or alongside evolution (e.g., Berkman and Plutzer 2011; Moore 2008; Moore and Cotner 2009; Nehm et al. 2009; Trani 2004). Consequently, a significant proportion of teachers either avoid or minimize evolution education in school and focus on alternative beliefs instead (e.g., creationism or intelligent design; Berkman et al. 2008; Rutledge and Mitchell 2002). Critically, this most likely means that educators transmit alternative beliefs and misconceptions to their students (Berkman and Plutzer 2011; Sickel and Friedrichsen 2013; Yates and Marek 2014) when in fact, they should have a crucial role as mediators between scientific evidence and society (Glaze and Goldston 2015). The Council of Europe resolution warned of the societal consequences of teaching creationism in biology classes. They predicted that “if we prevent our students from accessing scientific knowledge, we run the risk of their being unable to compete effectively with other students who are being educated in states where science has a key status” (Council of Europe 2007, p 17).

Due to the visible consequences of detrimental teaching practices, many researchers in teacher education have recognized the importance of examining teachers’ behavioral intention to teach evolution. Therefore, current research has identified and summarized multiple variables that may determine teachers’ behavioral intention (e.g., Großschedl et al. 2014; Nehm et al. 2009; Pobiner 2016; Sickel and Friedrichsen 2013; Smith 2010). For example, a teacher’s behavioral intention depends on cognitive, affective, and contextual variables, such as the attitude toward evolution and teaching it (e.g., Graf 2010; Großschedl et al. 2014), subjective norm (e.g., Griffith and Brem 2004), perceived behavioral control (e.g., Sanders and Ngxola 2009), personal religious faith (e.g., Downie and Barron 2000; Graf and Soran 2010; Trani 2004), perceived usefulness (e.g., Salman and Güven 2021), knowledge about evolution (e.g., Griffith and Brem 2004; Nadelson and Nadelson 2010; Rutledge and Mitchell 2002; Tekkaya et al. 2012; Trani 2004), and socio-demographic variables (e.g., Clément 2015; Deniz and Borgerding 2018; Goldston and Kyzer 2009). However, while theoretical literature reviews identified a conglomerate of determinants for behavioral intention, the presented empirical studies are limited to only examining isolated variables. Thus, we aim to systematically investigate primary factors that foster or hinder pre-service teachers’ behavioral intention to teach evolution in biology classrooms.

We focused particularly on pre-service biology teachers as they are still forming their teaching personalities. We consider our contribution relevant because the examined determinants of behavioral intention can inform adequate teacher preparation and further professional development at an early stage. Moreover, undesirable teaching behaviors can be understood and resolved early onward and do not solidify into rigid idiosyncratic behaviors over years of teaching practice (Tschannen-Moran et al. 1998).

To analyze variables that foster or hinder the behavioral intention to teach evolution in biology classrooms more systematically, we draw on the theory of planned behavior (Ajzen 1985, 1991). This approach from behavioral psychology holds that the determinants of attitude toward a behavior, subjective norm, and behavioral control determine a behavioral intention and result in a behavior. The theory is versatile and can be used to analyze a wide range of behavioral intentions and behaviors (Ajzen 1991; Francis et al. 2004). Thus, it is frequently used by interdisciplinary researchers in, for example, technology use in education (e.g., Lee et al. 2010; Sadaf et al. 2012; Teo 2012) and general educational settings (Heuckmann et al. 2020; Kilic 2012; Knauder and Koschmieder 2019; MacFarlane and Woolfson 2013; Martin and Kulinna 2004). We used this theory to analyze pre-service teachers’ behavioral intentions rather than behavior because pre-service teachers have little to no experience with teaching evolution. Furthermore, the behavioral intention has the strongest predictive power for someone’s behavior and can thus be used as a proximal measure of behavior (Francis et al. 2004). Beyond that, this theory recommends extending the analyses with background factors that are important to the specific context being studied. Including background factors can increase the variance explained in the target variable (e.g., Ajzen and Fishbein 2005). Accordingly, we extended the theory of planned behavior with additional variables (e.g., personal religious faith, perceived usefulness, and knowledge about evolution), which are repeatedly reported as important determinants of the behavioral intention to teach evolution.

## Theoretical background

### Behavioral intention and behavior

Behavioral intention is a variable that is based on someone’s motivation and indicates the strength of willingness and effort a person would invest in performing a behavior. Thus, someone who firmly intends to perform a behavior most likely acts on it (Ajzen 1985, 1991; Ajzen and Fishbein 2005). Similar patterns have been discovered in previous studies in the behavioral intention to teach evolution and the behavior of pre-service and in-service teachers. In particular, it was evident that some teachers only taught evolution if they were personally convinced of its merits. Generally, the country or state and its local educational curriculum stipulate the extent to which evolution should be taught in biology classes. The local educational requirements differ significantly in that some prescribe teaching evolution as early as elementary school age, and others ban the topic (Deniz and Borgerding 2018; Lerner et al. 2012; Siani and Yarden 2020). However, even if teaching evolution is required, the requirements for teaching evolution are not always heeded in biology classrooms, and teaching evolution appears to be under the volitional control of teachers (Moore 2002). Thus, not all teachers adequately meet teaching requirements. Teachers’ behavioral intention to teach evolution in their classes varies considerably. Studies repeatedly report that pre-service and in-service biology teachers do not accept evolution, doubt its epistemological status, or teach alternative beliefs such as creationism (Berkman and Plutzer 2011; Bönisch 2010; Deniz et al. 2011; Graf 2010; Graf and Soran 2010; Großschedl et al. 2014; Kilic 2012; Nehm and Schonfeld 2007; van Dijk 2009).

### Attitude toward a behavior

The variable attitude toward a behavior describes to what extent someone evaluates a particular behavior as positive (accepting) or negative (rejecting). If someone has a positive attitude toward a behavior, that person is more likely to have the behavioral intention to engage in the behavior (Ajzen 1991; Lee et al. 2010). Regarding the behavioral intention to teach evolution, it can be assumed that pre-service teachers’ acceptance or rejection of teaching evolution is crucial to whether they intend to teach evolution or not (Deniz and Sahin 2016; Großschedl et al. 2014; Kilic 2012; Smith 2010). Furthermore, an international comparison showed that pre-service teachers’ acceptance of evolution is relatively high in Germany (Kuschmierz et al. 2020; Miller et al. 2006). Graf (2010) showed that only 15% of pre-service teachers and 7% of pre-service biology teachers showed a negative attitude toward teaching evolution. Although this rate is comparatively low, it accounts for a considerable proportion of pre-service teachers. With a few exceptions (Southcott and Downie 2012), research findings on the historical development of pre-service teachers’ attitudes revealed a change in acceptance. Findings indicated a decline in acceptance and an increase in the rejection rate in the last three decades (Downie and Barron 2000; Miller et al. 2006; Plutzer and Berkman 2008; Unsworth and Voas 2018). Thus, it is crucial to maintain and strengthen a positive attitude toward teaching evolution. Regarding the relationship between attitude and behavioral intention, we assume H1:

#### H1

Pre-service teachers’ attitude toward teaching evolution is predictive of behavioral intention to teach evolution in biology classrooms.

### Subjective norm

The subjective norm describes the extent to which someone feels pressured by significant others to follow or not follow certain behavior. If the social environment supports the behavior in question, the person’s behavioral intention will probably increase (Ajzen 1991). Studies have shown that people are generally more inclined to adopt behavior or communicative positioning that strengthens their relationships with individuals with whom they have essential joint commitments. This adaptation to the expectations of significant others or social norms is particularly pronounced among newcomers to a group and could correspondingly also apply to pre-service teachers, who are soon to be teaching in the new school environment (Humphrey and Aime 2014; Kahan, 2010; Moreland 1985). For pre-service biology teachers, authoritative colleagues, students’ parents, friends, family, and superiors could represent significant others decisive for the behavioral intention to teach evolution (Humphrey and Aime 2014; Kilic 2012; Siani et al. 2022). The findings of Brem et al. (2003) indicated that college-educated individuals associated teaching evolution with a “negative ‘spin’ […], seeing it as decreasing spirituality, increasing selfishness and racism, and interfering with one’s sense of purpose and self-determination” (p. 198). Furthermore, studies discerned that teachers neglected to teach evolution because they felt pressured by others to teach creationism rather than evolution (e.g., by students’ religious parents), were suspended from school, or were legally accused (Asghar et al. 2007; Balgopal 2014; Berkman et al. 2008; Graf and Lammers 2010; Moore and Karen 2005).

Moreover, the subjective norm can predict the attitude toward a particular behavior (Lung-Guang 2019; Teo 2012; Venkatesh and Davis 2000). It is assumed that the social groups people identify with shape their perception and attitude (Torcello 2016). For pre-service teachers, this could mean that in addition to their behavioral intention, they may also adapt their attitude to the expectations of significant others. Thus, society-oriented variables such as social barriers, fear of disapproval, or immorality concerns could cause alterations in the attitude toward teaching (Arthur 2013). The subjective norm may also indirectly affect individuals’ behavioral intention to teach evolution. Based on these findings, we formulate H2 and H3 as follows:

#### H2

Pre-service teachers’ subjective norm is predictive of the behavioral intention to teach evolution in biology classrooms.

#### H3

Pre-service teachers’ subjective norm is predictive of someone’s attitude (and indirectly affects the behavioral intention to teach evolution in biology classrooms).

### Perceived behavioral control

Perceived behavioral control reflects the extent to which someone feels competent, skilled, and equipped with resources to control and perform a behavior (Ajzen 1991). When individuals have a high level of perceived behavioral control over a particular behavior, they are more likely to perform it (Ajzen 1991). For example, if pre-service teachers are confident that they have a high perceived behavioral control over teaching evolution, they will probably follow the behavioral intention. Conversely, a low level of perceived behavioral control can decrease the behavioral intention to teach evolution (Kilic 2012). A low perceived behavioral control can result from self-concern or the feeling of being unprepared. Additionally, the feeling of lacking materials may lead to losing perceived behavioral control over teaching evolution (Griffith and Brem 2004; Kilic 2012; Sanders and Ngxola 2009). Drawing on these findings, we hypothesize H4:

#### H4

Pre-service teachers’ perceived behavioral control predicts the behavioral intention to teach evolution in biology classrooms.

### Personal religious faith

Personal religious faith is a variable reflecting individual religiosity (Beniermann 2019). Prior studies suggest that religiosity relates negatively to accepting evolution and its teaching. Simultaneously, it was detected that it indirectly affects the behavioral intention to teach evolution (Betti et al. 2020; De Smedt and De Cruz 2020; Downie and Barron 2000; Moore 2002; Trani 2004). The negative correlation between personal religious faith and someone’s attitude can be attributed to individuals’ beliefs shaping the lens through which information such as scientific evidence is filtered (Glaze 2013). If individuals perceive a conflict between their fundamental religious beliefs and scientific explanations of evolution, they will probably reject the latter (Glaze 2013; Köse 2010; Meadows et al. 2000). Consequently, religious pre-service teachers are more likely to reject evolution in their classrooms (Deniz and Sahin 2016; Graf and Soran 2010; Großschedl et al. 2014). Furthermore, they might teach evolution only to a limited extent (Grogan 2020; Trani 2004). Despite the high probability that religious individuals tend to reject evolution, some religious believers accept evolution (Downie and Barron 2000; Levesque and Guillaume 2010), including religious teachers who teach evolution in biology classrooms (Silva et al. 2015; Trani 2004) and evolutionary biologists who see no conflict between scientific knowledge and faith as a worldview orientation (Miller 2002; Silva et al. 2015). Excluding the last presented exceptions, we posit H5:

#### H5

Pre-service teachers’ personal religious faith predicts the attitude (and indirectly affects the behavioral intention to teach evolution in biology classrooms).

### Perceived usefulness

In this context, the perceived usefulness encompasses the recognized advantages of behavior for a person and their performance at the job. This variable is regularly used to predict the behavioral intention within the technology acceptance model (Davis 1989). However, this construct has also been adopted for the theory of planned behavior (Sadaf et al. 2012). In initial conceptualizations, a disagreement occurred regarding whether perceived usefulness is a direct predictor of the behavioral intention or whether their relationship is mediated by the attitude (Davis 1989). Recent studies demonstrated that perceived usefulness can function as a strong, direct predictor of attitude and an indirect one of behavioral intention. Previous studies showed that the attitude is expected to be high when the perceived usefulness is high (Cheng 2018; Sadaf et al. 2012). Although the variable perceived usefulness has received minimal attention in teacher education, it can be an essential extension to the analysis of the behavioral intention to teach evolution. Teachers should be aware of the usefulness of evolution (Salman and Güven 2021). Therefore, our study suggests H6:

#### H6

Pre-service teachers’ perceived usefulness is a predictor of the attitude (and indirectly affects the behavioral intention to teach evolution in biology classrooms).

### Knowledge about evolution

This variable covers someone’s knowledge about evolution and underlying key mechanisms, such as natural selection (Anderson et al. 2002). Although pre-service teachers are expected to demonstrate a solid knowledge about evolution, their knowledge frequently does not correspond to the current scientific understanding of evolution (Graf and Soran 2010; Großschedl et al. 2018; Kuschmierz et al. 2020; Nehm et al. 2009). Insufficient knowledge about evolution is problematic, as pre-service teachers will have an important role in disseminating scientific knowledge to students (Glaze et al. 2015). Teachers must possess a thorough knowledge about evolution and be well-prepared to teach evolution (Rutledge and Mitchell 2002; Sickel and Friedrichsen 2013). If pre-service teachers are not adequately qualified, this can lead to inaccurate communication about evolution and even complete avoidance of teaching it (Balgopal 2014; Berkman et al. 2008; Glaze and Goldston 2015). Recent studies accentuated that knowledge about evolution is positively intertwined with the behavioral intention to teach evolution. Thus, it constitutes a necessary determinant (Griffith and Brem 2004; Rutledge and Mitchell 2002; Sickel and Friedrichsen 2013; Smith 2010).

However, profound knowledge about evolution does not inevitably increase behavioral intention (Nehm and Schonfeld 2007). Research on the relationship between knowledge about evolution and teaching evolution yielded inconsistent results (Balgopal 2014; Großschedl et al. 2014; Nehm and Schonfeld 2007). Inconsistencies may be attributable to the knowledge-behavior gap, which can also rigorously guide examining the relationship between knowledge and behavioral intention as a predictor of behavior. Rimal (2000) found that the correlation between knowledge and behavior depends on the ability to exert personal control over the behavior in question. Other studies indicated that updated knowledge about evolution can enhance the feeling of being prepared, responsible for teaching evolution in class, and perceived behavioral control (Griffith and Brem 2004; Nadelson and Nadelson 2010; Tekkaya et al. 2012). Individuals with a higher perceived behavioral control also exhibit stronger correlations between knowledge about evolution and behavioral intention than individuals with a lower one (Rimal 2000). Therefore, knowledge about evolution and perceived behavioral control are fundamental to investigating the behavioral intention in question (Griffith and Brem 2004; Knauder and Koschmieder 2019). In consideration of these findings, we hypothesize H7 and H8:

#### H7

Pre-service teachers’ knowledge about evolution predicts the behavioral intention to teach evolution in biology classrooms.

#### H8

Pre-service teachers’ knowledge about evolution predicts perceived behavioral control (and indirectly affects the behavioral intention to teach evolution in biology classrooms).

## Research model

**Fig. 1:**
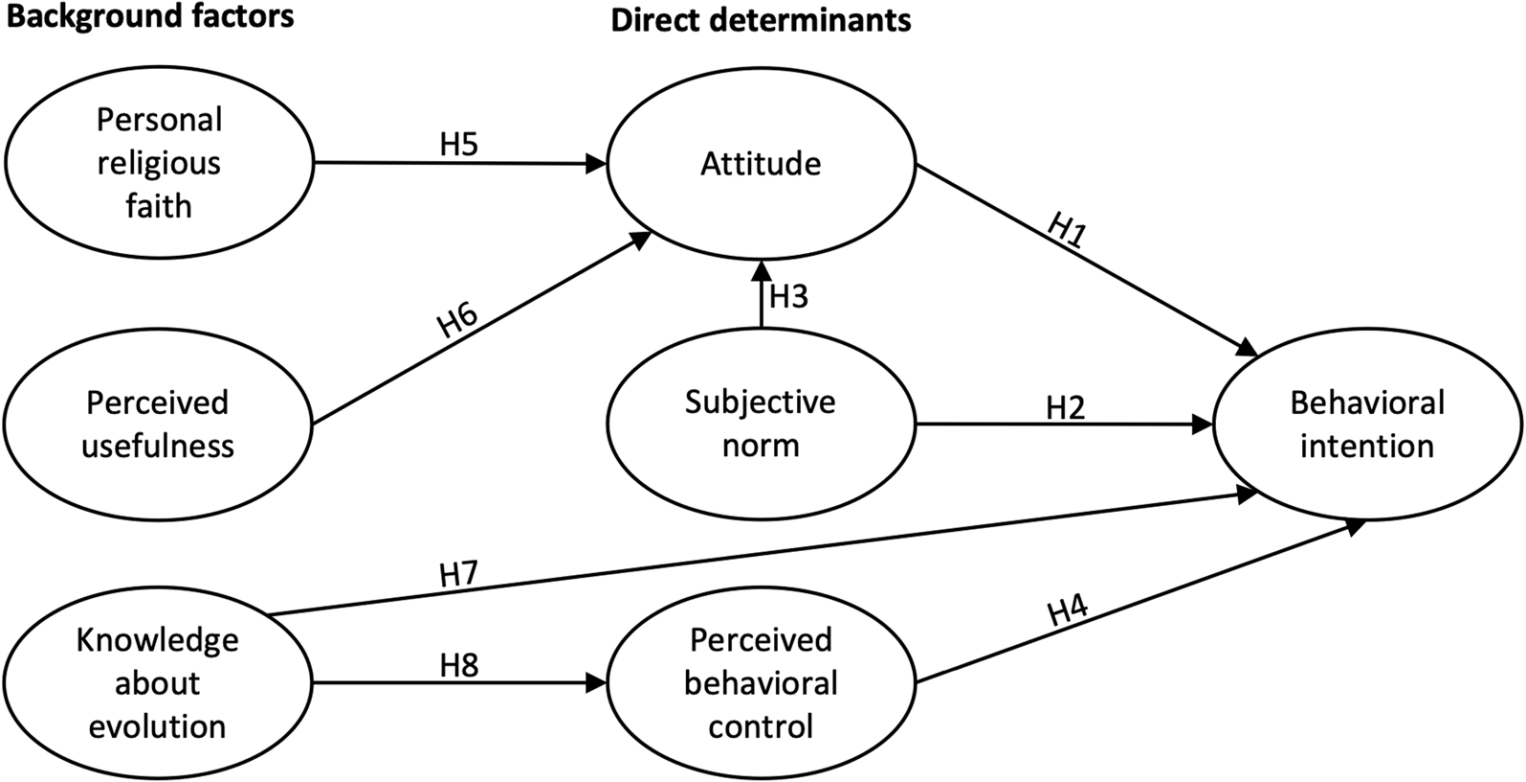
Theoretical model and research hypotheses

We analyzed the variables that foster or hinder teachers’ behavioral intention to teach evolution in biology and promote adequate teacher preparations and further professional development. We employed the validated theory of planned behavior (Ajzen 1985, 1991; Ajzen and Fishbein 2005) and complemented it with additional variables (i.e., personal religious faith, perceived usefulness, and knowledge about evolution) that are repeatedly reported as essential determinants for the behavioral intention to teach evolution (e.g., Cheng 2018; Clément 2015; Deniz and Borgerding 2018; Griffith and Brem 2004; Trani 2004). The proposed model, including the eight hypotheses, is presented in Fig. 1.

## Methods

To address the research aim, we conducted a quantitative cross-sectional study on teacher education. We employed an online questionnaire to collect the data and used them to conduct a structural equation model (Gefen et al. 2011; Kamel and Guillaume 2019). We followed the recommendations for a two-stage process by first validating the measurement model and subsequently employing the structural equation model to analyze our hypotheses (Gefen et al. 2011; Gerbing and Anderson 1988).

### Questionnaire development and measures

The questionnaire was divided into two sections. The first section covered the theoretically derived constructs. To develop the constructs of the theory of planned behavior and generate the questionnaire (Ajzen 1991), we followed the guideline of Francis et al. (2004). This guideline clearly instructs how to create the constructs and underlying items. For example, it includes sample items that should be adapted to the context under study. According to the guideline, we (1) outlined the target sample; (2) defined the behavior with respect to the target, action, context, and time (TACT); (3) opted for measurement methods (e.g., for the behavioral intention); (4) discussed the advantages and disadvantages of teaching evolution; (5) ascertained people who can be considered as authoritative and influential significant others; and (6) determined what increases or decreases perceived behavioral control. We pinpointed the clear focus of the study because a lack of granularity renders ambiguity unavoidable (Lee et al. 2010). After establishing a clear focus, we developed the items of the constructs of behavioral intention (four items), attitude (nine items), subjective norm (four items), and perceived behavioral control (three items; Francis et al. 2004).

For the constructs not described in the theory of planned behavior, we chose items and instruments that were already available. Therefore, we adopted the validated perceived usefulness construct by Davis (1989) to the study’s context and employed three items. We used the Personal Religious Faith 2.0 (PERF 2.0; Beniermann 2019) to assess participants’ religious beliefs. This measure not only captures the religiosity of single denominations but also covers a wide range of monotheistic belief systems and is appropriate for the targeted sample of pre-service teachers. Furthermore, this measure and its 10 items were evaluated and modified by experts from related research fields such as religious sciences, psychology, and sociology to ensure content validity (Beniermann 2019). We additionally employed the Conceptual Inventory of Natural Selection (CINS; Anderson et al., 2002) as it is a widely used measure to determine the knowledge about evolution through natural selection of pre-service teachers (Anderson et al. 2002; Nehm and Schonfeld 2008). The CINS comprises 20 items in a multiple-choice test format (one correct and three incorrect answers). The response options of the items cover 10 evolutionary key concepts (e.g., natural resources, variation within a population, and differential survival) and distractors based on alternative misconceptions (Anderson et al. 2002). Sufficient validity of the CINS was ensured by its being designed or examined by subject area experts (content validity; Nehm and Schonfeld 2008; Tekkaya et al. 2011), verified by independent content experts (face validity; Anderson et al. 2002), and inspected by principal component (construct validity; Athanasiou and Mavrikaki 2014; Pinxten et al. 2020) and correlation analyses (convergent or/and discriminant validity; Fiedler et al. 2019; Nehm and Schonfeld 2008). Additionally, we included one marker variable (on the motivation to engage in sports) that was theoretically and statistically unrelated to the others. It was used to strengthen our arguments against common method bias and ensure discriminant validity (Podsakoff et al. 2003; Semin et al. 2005).

The second section contained, among others, single self-assessment items to better understand the sample and the context in which the study took place. These items capture socio-demographic data; knowledge and feelings regarding teaching evolution; interest in general biological topics; the semester; and the teacher’s training school type. Furthermore, we adopted and marginally modified three items from Nehm et al. (2009) to collect additional data on the conflict between evolution and religiosity, teachers’ preferred approaches to teaching (evolution, creationism, both), and teachers’ preferred beliefs (evolution, creationism, both). The questionnaire ended with a request to rate the study’s usefulness and the conscientiousness of answering the questionnaire.

Overall, most items were based on a 7-point Likert scale, where a value of 1 indicated a low (or negative) and 7 a high (or positive) expression of the respective characteristic. The items of the CINS were scored dichotomously.

### Recruitment and sample

The enrolled study participants were German pre-service biology teachers recruited over four semesters. The pre-service teachers were asked to participate in the online questionnaire via an anonymous link to the survey platform Qualtrics. Furthermore, they were free to forward the link to other pre-service biology teachers. The questionnaire contained an informed consent form, information about the data protection, anonymity, and confidentiality of the survey to minimize the risk of common method bias, for example, social desirability bias. Moreover, it was noted that no incentives were offered for participation (King and Bruner 2000; Podsakoff et al. 2003). The participants were informed about the definition of what is meant by “teaching evolution” (see Additional file 1); the procedure and length of the study; the requirements for participation; the absence of risks associated with participating; and the contact details of the study administrator. Moreover, we encouraged the pre-service teachers to select the answers that best applied to them and complete the survey diligently to avoid overlooking inversely worded items.

In total, *N* = 339 pre-service biology teachers participated in our study. Of these, 30 pre-service teachers were excluded (see the “Preliminary analysis and data preparation” section for more details). Accordingly, the data of *n* = 309 (91.2%) pre-service teachers were included for further descriptive and inferential statistical analyses. The participants needed *M* = 56.2 min (*SD* = 23.5) to answer the questionnaire, found its implementation useful (*M* = 5.0, *SD* = 1.4), and indicated that they had completed it conscientiously (*M* = 6.4, *SD* = 0.7).

The pre-service teachers were, on average, *M* = 25.0 years old (*SD* = 2.1), and 77.2% were female. The denominational affiliation was distributed as follows: 50.2% Catholics, 23.3% Protestants, 6.1% Muslim denominations, 2.3% members of the Christian Free Church, 1.0% members of the Orthodox Church, 0.6% Hindus, 12.9% atheists, and 3.6% others. Utilizing the single self-assessment items, we ascertained that 91.7% of pre-service teachers saw no conflict between evolution and religious beliefs. Additionally, 72.1% of the participants favored teaching evolution to their students in biology classes, 0% favored creationism, and 27.9% wanted to teach both together. Moreover, 83.3% of the participants reported that they preferred to believe in or accept evolution, 2.8% voted for creationism, and < 14.0% favored both simultaneously. The pre-service teachers indicated that they were strongly interested in general biological topics (*M* = 6.3, *SD* = 0.8), rated their knowledge as moderate (*M* = 4.7, *SD* = 1.2), and reported that they would feel comfortable teaching evolution (*M* = 5.1, *SD* = 1.3). The participants were in their *M* = 2.8 (*SD* = 1.0) of four master’s semesters. They studied to become teachers at schools that educate students for non-academic (special education [29.4%], lower secondary and comprehensive school [29.8%], or vocational school [1.9%]) and academic track (gymnasium and upper comprehensive school [38.8%]). The curriculum of all school types mandates teaching evolution (Secretariat of the Standing Conference of the Ministers of Education Cultural Affairs of the Länder in the federal republic of Germany [KMK] 2020).

### Data analysis

#### Preliminary analysis and data preparation

We used IBM SPSS Statistics (SPSS) version 28.0 for data preparation, including screening, purging, and recoding the data of *N* = 339 cases (International Business Machines Corporation [IBM] 2021). Moreover, we used IBM SPSS Statistics to adjust variances across observed variables to decrease the possibility of threats to the convergent validity (Muthén and Muthén 1998–2010) and to identify and handle missing data (see Additional file 2). After that, all further analyses were carried out in RStudio version 4.2.0 (R Core Team 2022; see Additional file 3). We filtered the pre-service teachers who studied teaching for a school type in which evolution is not usually taught (Ministry for School and Further Education of the State of North Rhine-Westphalia [MSW NRW] 2008) or cases that showed extreme statistical outliers regarding the variable age. Consequently, the overall dataset used for the descriptive and inferential statistical analyses comprised *n* = 309 cases, surpassing the minimum number of required cases to perform a structural equation model (Francis et al. 2004; Kline 2015; Westland 2010). When screening the data, we identified four items with missing values (per item < 2%) in the variables we intended to use for inferential statistics. Therefore, we performed a missing completely at random (MCAR) test. The test indicated that data were missing completely at random, χ^2^ (196,309) = 111.78, *p* < 1.00. We used the expectation-maximum (EM) algorithm with multiple imputations to prepare our dataset for further analysis (Enders 2006). Subsequently, we applied descriptive statistics for transparent sample descriptions and analyzed the preconditions of data (Fox and Weisberg 2019; Korkmaz et al. 2014; Neter et al. 1990). The results showed that the data were not normally distributed but that the variables’ relationship was approximately linear; the variance inflation factor (VIF) indicated no thread to multicollinearity as it was below the recommended threshold of 10 (1.06–1.66); and the Durbin-Watson score suggested no autocorrelation as it was close to the recommended threshold of 2 (1.74–2.10; Kline 2015; Neter et al. 1990). As the data showed no normal distribution, we relied on a robust maximum likelihood method (MLM) for further analyses. The advantages of this estimator are that standard errors are robust to non-normality, and it calculates robust fit indices using Satorra-Bentler scaled chi-square statistics (Kamel and Guillaume 2019; Satorra and Bentler 2001).

Before the validation, we tested and refined the measurement model using the RStudio package “lavaan” (Rosseel 2012) and analyzed the statistical values in combination with the underlying content to further ensure the content validity of the constructs (Chin 1998; Gefen et al. 2011; MacKenzie et al. 2011). It must also be noted that we did not use the variable knowledge about evolution as a latent construct but as a sum score (Cronbach’s α = .73, *M* = 13.23, *SD* = 3.66) due to its nature (e.g., 20 dichotomously rated items). Thus, the CINS was not required for validating the measurement model and was inserted as a sum score in the structural equation model.

#### Measurement model and validation

To assess the probability of common method bias, we applied Harman’s single-factor test. It showed that the maximal variance of the model explained by one variable was 38.6%. Hence, the explained variance fell below the upper limit of 50% and indicated no major threat to common method bias (Podsakoff et al. 2003). Additionally, the applied marker variable showed no to minor correlations with the other variables, strengthening the case against common method bias (Semin et al., 2005; Simmering et al. 2014).

We then evaluated the convergent validity of the measurement model using the factor loadings (λ), Cronbach’s α, composite reliability (CR), and average variance extracted (AVE). The loadings reflect how far the manifest variables are deemed a part of the latent variable. Loadings for each item should be at least .5 to signify indicator reliability (Chin 1998; Hair et al. 2010). Table 1 shows that the loadings were above the recommended threshold. The Cronbach’s α and CR are indicators for the construct reliability. The lowest Cronbach’s α was .76, and the lowest CR was .77. Thus, both exceeded the recommended threshold of .7 (Fornell and Larcker 1981; Kline 2015). The AVE indicates convergent validity and reveals how far one latent construct explains the associated indicators. The AVE did not fall below the recommended threshold of .5 for any of the constructs (Fornell and Larcker 1981).

**Table 1:** Validity and reliability for constructs

Variables	Items	Formulated items	λ	α	CR	AVE
Behavioral intention (BI)				.81	.82	.55
	BI1	It is essential to me to teach evolution.^1^	.86			
	BI2	I am enthusiastic about teaching evolutionary biology.^1^	.79			
	BI3	I expect to teach evolution.^1^	.64			
	BI4	I intend to teach evolution in a scientifically correct way.^1^	.61			
Attitude towards a behavior (AT)				.91	.91	.54
	AT1	Teaching evolution in biology classes is/would be… *useless – useful.*	.79			
	AT2	Teaching evolution in biology classes is/would be… *wrong – good.*	.73			
	AT3	Teaching evolution in biology classes is/would be… *uncomfortable – comfortable.*	.51			
	AT4	Teaching evolution in biology classes is/would be… *harmful – beneficial.*	.77			
	AT5	Teaching evolution in biology classes is/would be… *unpleasant – pleasant.*	.64			
	AT6	Teaching evolution in biology classes is/would be… *undesirable – desirable.*	.81			
	AT7	Teaching evolution in biology classes is/would be… *misleading – purposeful.*	.79			
	AT8	Teaching evolution in biology classes is/would be… *worthless – helpful.*	.87			
	AT9	Teaching evolution in biology classes is/would be… *ineffective – effective.*	.77			
Subjective norm (SN)				.83	.84	.58
	SN1	Most people I care about think that I *should – should not* teach evolution.^R^	.91			
	SN2	People whose opinions I value will encourage me to teach evolution in biology classes.^1^	.86			
	SN3	People whose opinions I value will recommend that I teach evolution in biology classes.^1^	.59			
	SN4	I follow the conviction of my college to make evolution a significant part of my biology teaching.^1^	.66			
Perceived behavioral control (PBC)				.76	.77	.53
	PBC1	My way of teaching evolution is clear and understandable.^1^	.88			
	PBC2	I find it challenging to teach evolution.^1R^	.58			
	PBC3	I find it easy to teach the information about evolution that I have planned to teach.^1^	.77			
Perceived usefulness (PU)				.92	.92	.79
	PU1	Teaching evolution in biology classes increases my effectiveness in my job.^1^	.93			
	PU2	Teaching evolution in biology classes improves my performance in my job.^1^	.84			
	PU3	Teaching evolution in biology classes increases my productivity.^1^	.90			
Personal religious faith (PRF)				.96	.96	.71
	PRF1	I believe in God.^1^	.90			
	PRF2	I feel that God exists.^1^	.92			
	PRF3	I think there are good arguments for the existence of God.^1^	.87			
	PRF4	I would describe myself as a faithful person.^1^	.87			
	PRF5	Without faith, my life is/would be pointless.^1^	.78			
	PRF6	I believe there is a heaven.^1^	.63			
	PRF7	I pray and believe that my prayers can change what happens (in the future).^1^	.85			
	PRF8	I feel most fulfilled when I am in a close connection with God.^1^	.88			
	PRF9	Because of my faith, I have hope for life after death.^1^	.80			
	PRF10	My life is meaningful because I am wanted by God^1^	.88			

^1^Response format: Items were rated on a 7-point Likert scale (*strongly disagree – strongly agree*)

^R^Inversely worded items were recoded prior to the analyses

Subsequently, we assessed the discriminant validity. Discriminant validity is given when one variable that is theoretically unrelated to another is also statistically unrelated. In this case, discriminant validity can be measured using the Fornell-Larcker criterion. Discriminant validity exists when the square root of the AVE of a latent construct (bold diagonal in Table 2) exceeds its correlation with the other latent constructs within the model, as this indicates that the modeled constructs can be reliably separated (Fornell and Larcker 1981).

**Table 2:** Discriminant validity

Variable	*M*	*SD*	1	2	3	4	5	6
1 Behavioral intention	6.03	0.91	**.74**					
2 Attitude	6.13	0.76	.67**	**.74**				
3 Subjective norm	5.95	1.00	.60**	.58**	**.76**			
4 Perceived behavioral control	4.98	1.07	.44**	.32**	.36**	**.73**		
5 Perceived usefulness	4.31	1.21	.31**	.36**	.26**	.20**	**.89**	
6 Personal religious faith	3.47	1.68	–.17**	–.28**	–.24**	.00	–.10	**.84**

*p < .05 level (2-tailed). **p < .01 level (2-tailed). Values on the diagonal (bold) are the square root of the average variance extracted (AVE), while the off diagonals show Pearson’s product-moment correlation coefficients

Our data revealed discriminant validity as the square root of each AVE was always higher than the correlation between the respective construct with the other ones. To strengthen the robustness of the discriminant validity, we further conducted the heterotrait-monotrait (HTMT) method. The results are summarized in Table 3. The values of the HTMT do not exceed the threshold of .85 and thus meet the recommended criteria (Henseler et al. 2015).

**Table 3:** Heterotrait-monotrait

Variable	1	2	3	4	5	6
1 Behavioral intention						
2 Attitude	.78					
3 Subjective norm	.74	.66				
4 Perceived behavioral control	.54	.36	.44			
5 Perceived usefulness	.33	.39	.28	.24		
6 Personal religious faith	.16	.28	.25	.07	.07	

Shaded boxes are the standard reporting format for the HTMT procedure

Overall, the measurement model showed reliable and valid results. Hence, we continued with the second stage of the structural equation model analyses. We computed fit indices, analyzed regression and determination coefficients, effect sizes, and presented indirect and total effects.

## Results

### Structural equation model

First, we calculated the fit indices of our structural equation model. The analysis of the central χ^2^ distribution (Jöreskog, 1969) showed that our model did not hold an exact central χ^2^ distribution, χ^2^ (508,309) = 820.63, *p* < .001. Such a non-central distribution is not uncommon. Nevertheless, alternative tests must be used to analyze the data and determine whether this model is a helpful approximation for the research aim in question (Gefen et al. 2011). Accordingly, we analyzed the fit indices: the root mean square error of approximation (RMSEA), standardized root mean square residual (SRMR), comparative fit index (CFI), and Tucker-Lewis index (TLI). The RMSEA and SRMR showed a (very) good approximate fit when showing values below .5 and a (good) approximate fit with values below .8 (Browne and Cudeck 1992; Hu and Bentler 1999; Kamel and Guillaume 2019). Our model revealed a good to approximate fit with a robust RMSEA = .045 and SRMR = .077. Additionally, the robust CFI = .952 and the robust TLI = .947 indicated a very good model fit as they surpassed the threshold of .9 for a good fit (Kamel and Guillaume 2019; Marsh et al. 2004; Satorra and Bentler 2001).

**Fig. 2:**
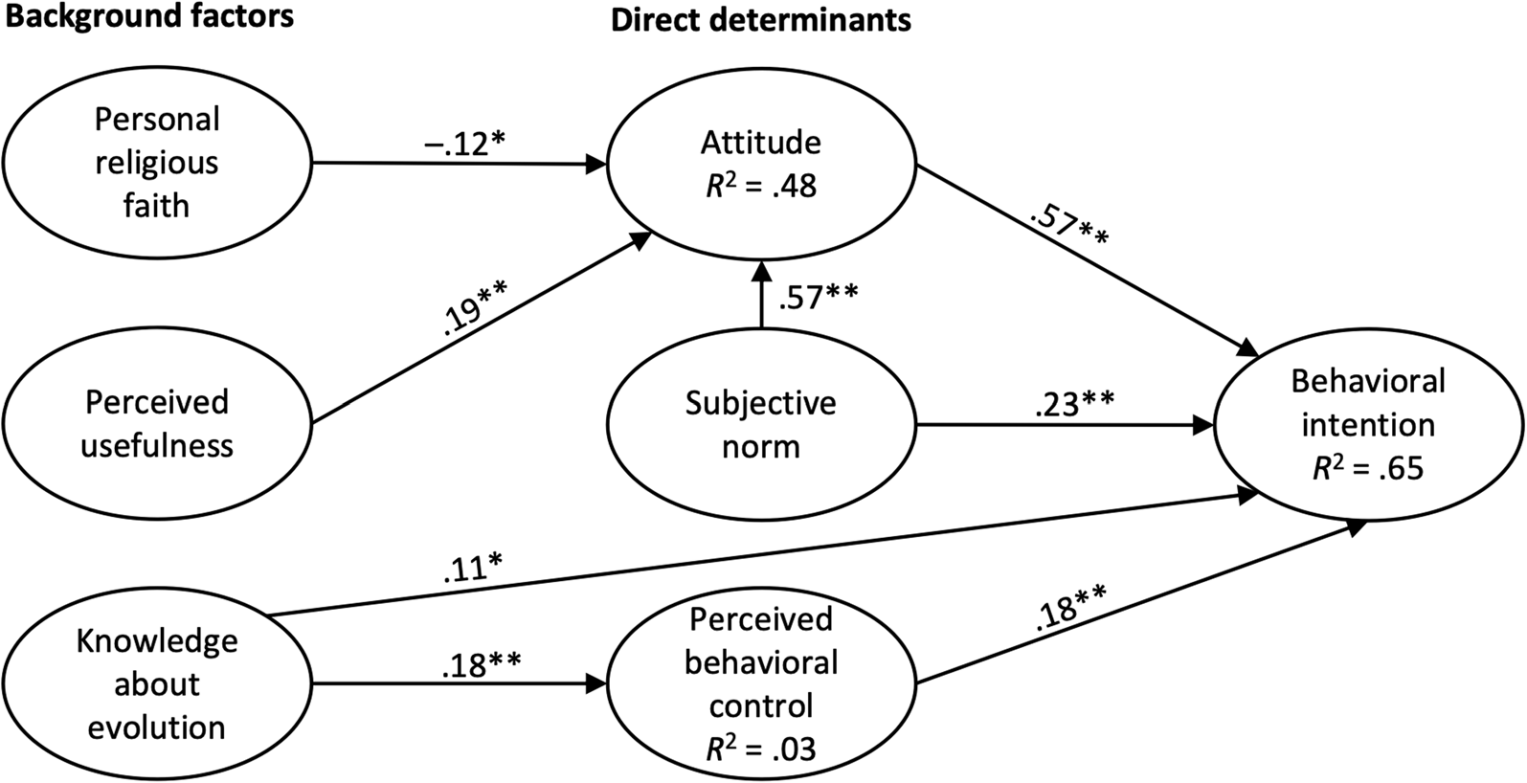
Results of the structural equation model. Critical *t*-values. *1.96 (*p* < .05); **2.58 (*p* < .01).

Second, we investigated standardized regression and determination coefficients (see Fig. 2). The results showed that the attitude (β = .57, *p* < .001), subjective norm (β = .23, *p* = .002), and perceived behavioral control (β = .18, *p* < .001) significantly affected the behavioral intention to teach evolution in biology lessons. Moreover, the subjective norm (β = .57, *p* < .001), personal religious faith (β = –.12, *p* = .027), and perceived usefulness (β = .19, *p* = .001) had a significant effect on the attitude of teachers and are predictive for 48.4% of the variance of attitude (Cohen 1988). Knowledge about evolution affected behavioral intention (β = .11, *p* = .028) and perceived behavioral control (β = .18, *p* = .002). Moreover, knowledge about evolution explained only a small amount (3.4%) of the variance of perceived behavioral control. Overall, the variables attitude, subjective norm, perceived behavioral control, and knowledge about evolution led to 65.4% of the variance in the behavioral intention being explained, indicating a high goodness of fit (Cohen, 1988).

Third, based on recommendations for good scientific reporting of structural equation models (Gefen et al. 2011), we calculated the effect size ƒ^2^. This effect size is used to determine the substantive significance and, thus, the importance of the independent variables for explaining the variance of one dependent variable (Cohen 1988). To calculate the effect size, the change in the explained variance *R*^2^ must be examined when one of the paths, from the independent variables to a dependent variable, is included in or excluded from the model. Accordingly, we ran the model once in the original version and then repeated calculating the model by iteratively excluding one path at a time. Thereby, we examined the changes in the explained variance *R*^2^. Subsequently, we applied the following formula to obtain the effect size ƒ^2^: ƒ^2^ = *R*^2^_included_ – *R*^2^_excluded_ / (1 – *R*^2^_included_). Conventionally, ƒ^2^ values of .02 represent small, .15 medium, and .35 large effects (Chin 1998; Cohen 1988). Table 4 shows that almost all paths have a small effect. For example, excluding perceived usefulness reduced the variance explained in attitude from *R*^2^ = .484 to *R*^2^ = .461 (ƒ^2^ = .04). This effect is small but still higher than the change in the variance explained in attitude when dropping personal religious faith, which is only visible in the third decimal place as it decreased from *R*^2^ = .484 to *R*^2^ = .476 (ƒ^2^ = .02). Conversely, the change in the variance explained is strong when the paths from the subjective norm to the attitude or from attitude to behavioral intention are excluded. The former dropped from *R*^2^ = .484 to *R*^2^ = .212 (ƒ^2^ = .53), and the latter from *R*^2^ = .654 to *R*^2^ = .480 (ƒ^2^ = .50).

**Table 4:** Hypotheses testing

Hypotheses	Beta	*z*-values	Decision	ƒ^2^
H1: Attitude → Behavioral intention	.57	7.48**	Supported	.50
H2: Subjective norm → Behavioral intention	.23	3.06**	Supported	.03
H3: Subjective norm → Attitude	.57	7.42**	Supported	.53
H4: Perceived behavioral control → Behavioral intention	.18	3.54**	Supported	.09
H5: Personal religious faith → Attitude	− .12	− 2.21*	Supported	.02
H6: Perceived usefulness → Attitude	.19	3.29**	Supported	.04
H7: Knowledge about evolution → Behavioral intention	.11	2.19*	Supported	.03
H8: Knowledge about evolution → Perceived behavioral control	.18	3.12**	Supported	.04

Critical *z*-values. *1.96 (*p* < .05); **2.58 (*p* < .01)

Fourth, we focused the analyses on the mediators, indirect and total effects. The standard method for calculating these effects in the used R package “lavaan” is the delta method (Rosseel 2012; Sobel 1982). As this method is critically discussed (Zhao et al. 2010), we applied a bias-corrected and accelerated (BCa) bootstrap with 5000 resamples. The BCa bootstraps offer the general advantage of assigning precision measures to the data, such as confidence intervals (CIs), and controlling and checking the stability of the results, even after 5000 resamples. BCa bootstrapping is advantageous as it corrects both the bias and the skewness of the bootstrap distribution (Preacher and Hayes 2008). Taken all together, the results of the effects and bootstraps are depicted in Table 5. All indirect effects are significant, as the 95%-CIs do not cross zero, confirmed by the significant *z*-values. To determine the nature of the mediation, we included direct and indirect effects in the analysis. Since the direct and indirect paths were significant, we concluded that the model contained partial mediations. More specifically, we found complementary mediation in the paths from the subjective norm over the attitude to the behavioral intention and from knowledge about evolution over perceived behavioral control to behavioral intention. These results suggest that the effect of the subjective norm and knowledge about evolution on behavioral intention is reinforced through mediation (Zhao et al. 2010).

**Table 5:** Indirect and total effects

Effect type	Hypotheses	Beta	*SE*	*z*-value	95%-CI [LB, UB]	95%-CI [LB, UB]^1^
Indirect	SN → AT → BI	.33	.04	6.01**	[.18, .35]	[.18, .36]
	PRF → AT → BI	− .07	.02	− 2.13*	[− .06, − .00]	[− .07, − .00]
	PU → AT → BI	.11	.03	2.97**	[.03, .13]	[.03, .14]
	KaE → PBC → BI	.03	.01	2.35*	[.00, .04]	[.01, .05]
Total	SN → BI + (SN → AT*AT → BI)	.55	.06	7.63**	[.33, .57]	[.33, .59]
	KaE → BI + (KaE → PBC*PBC → BI)	.14	.03	2.93**	[.03, .17]	[.03, .17]

^1^BCa bootstrap with 5000 samples. Critical *z*-values. *1.96 (*p* < .05); **2.58 (*p* < .01); *BI* behavioral intention, *AT* attitude towards a behavior, *SN* subjective norm, *PBC* perceived behavioral control, *PU* perceived usefulness, *PRF* personal religious faith, *KaE* knowledge about evolution

##  Discussion

This study contributes to the prevailing discourse on teacher education by revealing that attitude, subjective norm, perceived behavioral control, personal religious faith, perceived usefulness, and knowledge about evolution are significant determinants of behavioral intention to teach evolution.

### Behavioral intention to teach evolution

Our study showed that the pre-service teachers had a high behavioral intention to teach evolution in biology classes. They were highly enthusiastic and motivated to teach in the future. No one stated that they would replace evolution with creationism in their teaching and they showed a high interest in general biological topics. When comparing the behavioral intention with suitable reference groups, the study of Großschedl et al. (2014), conducted in Germany, showed pre-service teachers with comparably high behavioral intention. Studies in other countries indicated lower behavioral intention than our sample (Deniz et al. 2011; Deniz and Sahin 2016; Nehm et al. 2009; Nehm and Schonfeld 2007). However, the study of Plutzer et al. (2020) on American science teachers showed encouraging improvements of evolution education instructions from 2007 to 2019. Kilic’s (2012) findings suggested that the sociocultural background may be related to the manifestation of the behavioral intention to teach evolution, as they concluded that German pre-service teachers are more disposed to teach evolution than Turkish ones.

Although the single self-assessment items revealed that no pre-service teacher would exclusively teach creationism, 27.9% indicated that they would teach evolution and creationism simultaneously. These results allow considerable latitude for interpreting how a pre-service teacher would contrast evolution and creationism in the class (similar to the findings of Moore and Cotner 2009). Addressing both components should not necessarily be viewed as detrimental. However, teaching must be carefully conducted (Scott and Branch 2003). For example, if such biology lessons aim to present the strengths of evolution and demarcate the boundaries between evolution and creationism, this could help students understand evolution even better (Reiss 2009).

### Behavioral intention, attitude toward teaching evolution, and subjective norm

In interdisciplinary research (e.g., Heuckmann et al. 2020; Knauder and Koschmieder 2019; Lee et al. 2010) and research on teacher education (e.g., Deniz and Sahin 2016; Großschedl et al. 2014), attitude is described as a critical determinant of behavioral intention. Similarly, we found that a positive attitude toward teaching evolution was the strongest determinant for pre-service teachers’ behavioral intention. This high intention was reflected by pre-service teachers perceiving teaching evolution in biology classes as desirable, helpful, effective, useful, and purposeful. The attitude toward teaching evolution was comparable to other German (Großschedl et al. 2014, 2018; Kuschmierz et al. 2020) and relatively high in comparison to international results (Miller et al. 2006). These results suggest that a positive attitude toward teaching evolution is relatively consistent, conflicting with other results suggesting attitudes are deteriorating (Downie and Barron 2000; Miller et al. 2006; Plutzer and Berkman 2008; Unsworth and Voas 2018).

The subjective norm was the second strongest predictor of behavioral intention. Consistent with Kilic (2012), significant others play a critical role in conditioning pre-service teachers’ behavioral intentions. Additionally, we revealed that this social sphere strongly affects the attitude toward teaching evolution and that the effect of subjective norms on behavioral intention is partially mediated through this attitude. The relationship between the three variables could be explained by pre-service teachers, as newcomers to their field, being more willing to adapt their attitudes and behavioral intention to their social environment than long-term members of such an environment (Humphrey and Aime 2014). The demographics of our sample may also contribute to the relationship between subjective norms, attitudes, and behavioral intention. Studies analyzing the *Big Five* personality traits suggested that individuals who are professionalizing in teaching (Üstüner 2017) or are female (Rubinstein 2005) are more likely to have agreeable personalities. High agreeableness implies that they are altruistic, cooperative, and accommodating. This tendency to agreeableness can be disadvantageous if significant others criticize the teaching of evolution, as pre-service teachers could be negatively swayed by this (Arthur 2013; Balgopal 2014).

### Attitude toward teaching evolution, personal religious faith, and perceived usefulness

The participants in our sample exhibited moderate personal religious faith. Only 2.8% of the pre-service teachers stated that they believed in and accepted creationism. The findings corroborate the “religion light” phenomenon observed in Germany, describing a religious orientation that is only minimally pursued (Ziebertz et al. 2003). Moreover, the findings indicate that the pre-service teachers’ personal religious faith had a small negative direct effect on attitude and a small negative indirect effect on behavioral intention. The research discourse on the effects of personal religious faith presented mixed results. Some results indicated that the attitude toward religion has no predictive power regarding accepting evolution or the preference to teach it (Großschedl et al. 2014), while others revealed a strong negative relationship (Graf and Soran 2010). The results might be explained by the fact that some people can reconcile scientific evidence about evolution and religious beliefs, showing no or minor correlations between personal religious faith and their attitude toward evolution. Others perceive a conflict that causes a strong negative relationship (cf. Köse 2010). This assumption would explain the small effect of personal religious faith on our sample’s attitude toward teaching evolution. Our results revealed that 91.7% saw no conflict between evolution and religious beliefs, and 14% of the pre-service teachers preferred to believe in and accept evolution and creationism simultaneously.

Additionally, the variable perceived usefulness had a small positive effect on the attitude toward teaching evolution, which was stronger than that of personal religious faith. Moreover, perceived usefulness is an indirect predictor of behavioral intention. The sense of usefulness of teaching evolution was moderate in our study, indicating that a significant proportion of the participants did not want to recognize or did not recognize its benefits. However, biology teachers must understand the importance of their subject (Nadelson and Nadelson 2010; Salman and Güven 2021). Evolution is the overarching principle for all areas of biology (Darwin 1859). Thus, it should be viewed as a tool that meaningfully connects all areas of biology and should be leveraged as such in teaching (Leopoldina 2017). To teach biology successfully, teachers should view evolution as enhancing their teaching effectiveness, productivity, and performance. Even if pre-service teachers do not appreciate its usefulness, they must understand that teaching evolution is part of their job, and ignoring it is even unlawful in some countries, such as Germany (e.g., KMK 2020).

### Behavioral intention, perceived behavioral control, and knowledge about evolution

The perceived behavioral control of the pre-service teachers was moderate to high and positively affected the behavioral intention. The results showed that most participants were confident that they would be able to teach evolution successfully, and they felt competent to conduct lessons in a planned manner. These findings are supported by Kilic (2012), who highlighted that German pre-service biology teachers estimated the ease of teaching evolution as higher than Turkish ones. Related studies also encountered contrasting results identifying limitations of perceived behavioral control, for instance, due to insufficient curriculum time, lack of materials, or the complexity of evolution (Kilic 2012; Nadelson and Nadelson 2010; Sanders and Ngxola 2009; Siani and Yarden 2022).

Our results further disclosed moderate knowledge about evolution. These results are comparable to other studies in Germany (Großschedl et al. 2014, 2018) and America (Nehm et al. 2013), which likewise revealed approximately moderate knowledge about evolution. Moreover, we confirmed that knowledge about evolution is predictive of behavioral intention to teach evolution (cf. Großschedl et al. 2014; Sickel and Friedrichsen 2013; Smith 2010). If knowledge about evolution increases, behavioral intention also increases. Problematically, despite only moderate knowledge about evolution, we found a relatively high behavioral intention to teach evolution. The pre-service teachers were already in the penultimate semester of their university education. Accordingly, they will likely be released into the teaching profession without their knowledge about evolution reflecting the current scientific understanding. Thus, despite teachers aiming to provide scientifically sound information, mediocre knowledge about evolution or mixed messages will most likely be transmitted to students. This can be particularly problematic because it can appear to students as if non-scientific information are being legitimized (Plutzer et al. 2020).

Additionally, we established that knowledge about evolution had a small positive effect on perceived behavioral control. These findings are consistent with Griffith and Brem (2004), who showed that teachers felt they could teach evolution more easily when they possessed up-to-date knowledge. Even familiarity with evolution enhances the feeling of being qualified to teach it (Nadelson and Nadelson 2010). We further detected that perceived behavioral control functioned as a mediator between knowledge about evolution and the behavioral intention to teach it. More explicitly, we ascertained that knowledge about evolution could strengthen the behavioral intention to teach evolution if perceived behavioral control was moderate. Thus, perceived behavioral control appears crucial for the intention to perform a behavior. The findings that perceived behavioral control functions as a moderator can spark future research determining whether perceived behavioral control might moderate the gap between knowledge and behavioral intention rather than the gap between knowledge and behavior (cf. Rimal 2000).

### Theoretical implications for research in teacher education

The implications of the discussed results can be summarized as follows:

First, we contributed to the current research by showing that the intention to teach evolution is likely to be shaped by the sociocultural backgrounds of pre-service teachers. This facilitates future studies that can examine the determinants in different sociocultural contexts.

Second, this study demonstrated the power of a behavioral psychology research perspective. For the first time, we combined the constructs of the theory of planned behavior with background factors in a single model and examined the various effects on the behavioral intention to teach evolution. We expanded the body of knowledge regarding variables previously empirically examined in isolation.

Third, we presented a profound and transparent research approach as we derived our research model from the validated theory of planned behavior and the guideline for constructing a questionnaire conforming with the theory (Ajzen 1991; Francis et al. 2004). Additionally, we followed common recommendations for creating a two-stage structural equation model (Gefen et al. 2011) and used well-established (e.g., Cohen 1988; Fornell and Larcker 1981) and recently developed and endorsed statistical approaches (e.g., Henseler et al. 2015). The disclosed study documents, the access to the data, and the stepwise description of the analyses allow repeating results and conducting similar studies with little economic effort. As far as replication of results is concerned, it must be explicitly ensured that the identical versions of programs and packages are used. Otherwise, the results may deviate. For future quantitative research, it would be desirable to examine other samples, such as instructors of science teaching methods or educators of other disciplines, diverse cultures, countries, and levels of expertise. Additionally, our research model could be extended to include other context-specific variables or be adapted to investigate different behavioral intentions, such as the teaching of other controversial biological topics. Besides quantitative studies, follow-up studies using a mixed-method or qualitative research approach could corroborate the reported findings and contextualize pre-service teachers’ instructional approach decisions.

Fourth, we found that the subjective norm is not only a predictor of behavioral intention but also of the attitude toward teaching evolution. This finding extends the theory of planned behavior and improves the analysis of the behavioral intention of pre-service teachers. Whether this extension of the theory holds benefits for other samples remains to be determined.

Fifth, although other disciplines have revealed that the variable perceived usefulness is an important predictor of someone’s attitude, this variable has received minimal attention in teacher education. Thus, like Salman and Güven (2021), we contributed to teacher education by highlighting the importance of the perceived usefulness for facilitating a positive attitude toward teaching evolution.

### Practical implications for teacher education and further professional development

We recommend that future studies, which analyze pre-service and in-service teachers’ intention to teach evolution, also survey the variables which we identified as important. Such a survey can reflect the status quo and provide a basis for tailoring educational interventions to the needs at hand. Since the most important determinants of behavioral intention to teach evolution are the attitude toward teaching evolution, subjective norm, and perceived behavioral control, these three should not be neglected in teacher education. Teacher education programs could promote a positive attitude toward teaching evolution by raising pre-service teachers’ awareness of their beliefs (Sanders and Ngxola 2009); intentionally addressing multifaceted attitudes toward teaching evolution; sensitively and respectfully fostering controversial discussions in a value-free space; and providing a safe space for acknowledging controversy (Griffith and Brem 2004; Hermann 2008). Furthermore, based on the subjective norm, pre-service teachers might benefit from assistance regarding conflict-prone contexts and conflict management strategies to cope, for instance, with social pressure and related challenges (Griffith and Brem 2004; Plutzer et al. 2020; Sanders and Ngxola 2009; Siani et al. 2022; Sickel and Friedrichsen 2013). Additionally, debating skills and understanding arguments for or against evolution could provide a more stable stance when feeling pressured (Asghar et al. 2010; Dotger et al. 2010; Glaze and Goldston 2015). Tactics to improve perceived behavioral control could include providing pre-service teachers with materials and strategies for teaching and enabling them to gain experience in how to teach complex topics such as evolution (Hermann 2008; Reiss 2022; Sickel and Friedrichsen 2013). It is important to note that unilateral fostering of perceived behavioral control can also lead to problems if its relationship to knowledge about evolution is neglected. An example of an unfavorable relationship would be when there is an unrealistically high perceived behavioral control and a low level of knowledge about evolution. This imbalance could lead to misconceptions being confidently passed on to students (cf. Yates and Marek 2014).

Furthermore, teacher education programs could inform pre-service teachers about religious leaders who reconcile religiosity and evolution to minimize pre-service teachers’ feelings of conflict between both approaches. This educational method could additionally involve creating awareness of a possible intuitive cognitive bias (Barnes and Brownell 2017; Hermann 2013). Individuals with a negative attitude toward teaching evolution could benefit from educational programs that discuss and reflect on multifaceted perspectives and strengthen teachers’ cultural competence (Barnes and Brownell 2017; Brem et al. 2003; Brownell et al. 2018; Meadows et al. 2000).

Additionally, we found that the pre-service teachers in our study require support to build a solid knowledge of evolution. Effective teacher education could include more intensive evolution courses (Ha et al. 2015; Nadelson and Southerland 2010; Siani and Yarden 2022). Moreover, teacher education programs could raise awareness of the usefulness of teaching evolution for pre-service teachers by enabling contact with approachable and authentic scientists (Nadelson and Hardy 2015; Tekkaya et al. 2012) and thematizing comprehensible examples from daily life science with relevance to pre-service teachers, such as the spread of viruses or vaccine development (e.g., Hillis 2007; Pobiner 2016; Smith 2010).

### Limitations

Despite the insightful findings, this study has limitations. First, the generalizability of its findings is limited (Hedges 2013). Although the findings are transferable to similar samples, they must be replicated to increase external validity.

Second, although many validity criteria for the CINS have been met, this measurement revealed limitations that should be considered when employing it. For example, limitations were evident in psychometric flaws showing low discriminability and many items with high and similar difficulty levels. Moreover, unlike open response measures, the CINS did not adequately capture individual, heterogeneous conceptions of natural selection (Nehm and Schonfeld 2008). Despite these shortcomings, the total CINS scores generally reflect reliable and valid inferences regarding participants’ understanding and are sufficient for this study (Nehm and Schonfeld 2008).

Third, we asked pre-service teachers to refer to past situations in school or place themselves in their future position as biology teachers when answering the questionnaire. We also requested that they answer the questions to the best of their ability and select the answers that best applied to them. However, in our study, we could not clarify the extent to which the surveyed pre-service teachers enacted their behavioral intention in daily school life.

Fourth, while we could explain a substantial amount of the variance in the behavioral intention to teach evolution, namely 65.4%, the flip side of the coin also showed that 34.6% of the variance remained unexplained. Therefore, it is necessary to investigate which additional variables can increase the explained variance.

## Conclusion

In summary, extant research in teacher education recognized the importance of studying variables that foster or hinder teachers’ behavioral intention to teach evolution as this can provide information about effective pre-service and in-service teacher education and prevent adverse teaching practices (e.g., Großschedl et al. 2014; Nehm et al. 2009; Pobiner 2016; Sickel and Friedrichsen 2013; Smith 2010). While researchers have identified many variables, these are often considered in isolation and lack a theoretical framework accounting for the various determinants shaping teaching intentions. Therefore, this study’s contribution was to systematically analyze variables that foster or hinder the behavioral intention to teach evolution in biology classrooms. We employed the theory of planned behavior with its associated variables behavioral intention, attitude, subjective norm, and perceived behavioral control (Ajzen 1985, 1991; Ajzen and Fishbein 2005). To adapt the theory to our study’s focus, we incorporated the background variables of personal religious faith, perceived usefulness, and knowledge about evolution. Additionally, we included socio-demographic variables in our analyses, as this helps contextualize the findings (e.g., Clément 2015; Deniz and Borgerding 2018; Goldston and Kyzer 2009; Griffith and Brem 2004; Salman and Güven 2021; Sanders and Ngxola 2009; Trani 2004). We conducted a structural equation model to test our hypotheses. The results supported all eight hypotheses of this study. Overall, our results illuminate primary determinants of behavioral intention to teach evolution and their relationships. Moreover, we have deepened the understanding of the requirements for effective teacher education and can promptly inform teacher education and further professional development.

## Supplementary Information

**Additional file 1. **This file contains the definition of teaching evolution.

**Additional file 2. **This file comprises the output of the Little’s test of missing completely at random (MCAR) and the multiple imputing by using the expectation-maximum (EM) algorithm in SPSS.

**Additional file 3. **This file encompasses the script for the analyses and the results of the article.

## Data Availability

The data supporting this study’s findings are available in the repository zenodo at https://doi.org/10.5281/zenodo.7082082.
