# Supplementary material for: Analyzing pre-service biology teachers’ intention to teach evolution using the theory of planned behavior
Source: Evolution (N Y). 2022 Nov 18;15(1):16. doi: 10.1186/s12052-022-00175-1 (PMC9673228; doi:10.1186/s12052-022-00175-1)
Supplement: Supplementary file 1 — Additional file 1. This file contains the definition of teaching evolution. [file 12052_2022_175_MOESM1_ESM.pdf]

Definition of teaching evolution

In this study, we used the following definition to clarify what we meant by "teaching evolution":

Teaching evolution describes the practice of teaching about the theory of evolution, respectively, about the scientific and intrinsically consistent description of the origin and change of biological units and biodiversity of life. The teaching of evolution summarizes continuous, natural development processes in the earth's history. The underlying evolutionary theory represents a product of time and reflects the current scientific understanding of evolution (Council of Europe, 2007; Darwin, 1859; Leopoldina, 2017; KMK, 2020).

References

Council of Europe. (2007). *Resolution 1580. The dangers of creationism in education*. https://assembly.coe.int/Documents/WorkingDocs/2007/EDOC11375.pdf

Darwin, C. (1859). *On the origin of species by means of natural selection, or the preservation of favoured races in the struggle for life*. D. Appleton.

Nationale Akademie der Wissenschaften Leopoldina. (2017). *Evolutionsbiologische Bildung in Schule und Universität [Evolutionary biology education in school and university]*. https://www.leopoldina.org/uploads/tx_leopublication/2017_Stellungnahme_Evolutionsbiologie.pdf

Secretariat of the Standing Conference of the Ministers of Education and Cultural Affairs of the Länder in the federal republic of Germany. (2020). *Bildungsstandards im Fach Biologie für die Allgemeine Hochschulreife [Educational standards in biology for the general university entrance qualification]*. Carl Link. https://www.kmk.org/fileadmin/Dateien/veroeffentlichungen_beschluesse/2020/2020_06_18-BildungsstandardsAHR_Biologie.pdf
