# Supplementary material for: Analyzing pre-service biology teachers’ intention to teach evolution using the theory of planned behavior
Source: Evolution (N Y). 2022 Nov 18;15(1):16. doi: 10.1186/s12052-022-00175-1 (PMC9673228; doi:10.1186/s12052-022-00175-1)
Supplement: Supplementary file 3 — Additional file 3. This file encompasses the script for the analyses and the results of the article. [file 12052_2022_175_MOESM3_ESM.html]

Script for the analyses of the article: Analyzing pre-service biology teachers’ intention to teach evolution using the theory of planned behavior


Code 

- Show All Code
- Hide All Code

# Script for the analyses of the article: Analyzing pre-service biology teachers’ intention to teach evolution using the theory of planned behavior

# 1 Aim

This script serves to explain and present the chosen analyses and
results of the research article. In addition, the data and syntax
excerpts can provide a first insight into a possible sequencing of the
analyses of a two-level structural equation model. To get R novices
started, this script begins with the installation of R and RStudio.

# 2 R and RStudio

## 2.1 Installing R and RStudio

1. Install the latest R
   version for your computer.
2. Download RStudio by following this Link and
   continue to the download area in the top right of the page.
3. Use the R Markdown
   guide for information on how to use R Markdown (Xie et al.,
   2018).

## 2.2 Installing and loading packages

Install and load all needed packages. You can either use the
functions `install.packages` (for installing the packages)
and `library`(for loading the packages from your library) or
you use the function `p_load`. The `p_load`
function is a wrapper for `library` and `require`.
It checks to see if a package is installed. If you have not installed
the package yet, it attempts to install the package from CRAN and/or any
other repository in the pacman repository list.

```
if(!require(pacman, quietly = TRUE)) install.packages("pacman")
p_unload("pacman", negate = TRUE)
p_load("rstudioapi")   # cite RStudio reference (Ushey et al., 2022)
```

## 2.3 Citing

Make sure to cite the applications/tools, versions, packages, and
*scripts* you use to credit developer/authors and ensure
repeatable (or even reproducible) science. Use this code to see how to
cite R `citation ()` and this `R.Version()` to
view information on your R version.

```
citation()             # cite R
## 
## To cite R in publications use:
## 
##   R Core Team (2022). R: A language and environment for statistical
##   computing. R Foundation for Statistical Computing, Vienna, Austria.
##   URL https://www.R-project.org/.
## 
## A BibTeX entry for LaTeX users is
## 
##   @Manual{,
##     title = {R: A Language and Environment for Statistical Computing},
##     author = {{R Core Team}},
##     organization = {R Foundation for Statistical Computing},
##     address = {Vienna, Austria},
##     year = {2022},
##     url = {https://www.R-project.org/},
##   }
## 
## We have invested a lot of time and effort in creating R, please cite it
## when using it for data analysis. See also 'citation("pkgname")' for
## citing R packages.
R.Version()            # see R version
## $platform
## [1] "aarch64-apple-darwin20"
## 
## $arch
## [1] "aarch64"
## 
## $os
## [1] "darwin20"
## 
## $system
## [1] "aarch64, darwin20"
## 
## $status
## [1] ""
## 
## $major
## [1] "4"
## 
## $minor
## [1] "2.0"
## 
## $year
## [1] "2022"
## 
## $month
## [1] "04"
## 
## $day
## [1] "22"
## 
## $`svn rev`
## [1] "82229"
## 
## $language
## [1] "R"
## 
## $version.string
## [1] "R version 4.2.0 (2022-04-22)"
## 
## $nickname
## [1] "Vigorous Calisthenics"
versionInfo()$citation # cite RStudio
## 
## To cite RStudio in publications use:
## 
##   RStudio Team (2022). RStudio: Integrated Development Environment for
##   R. RStudio, PBC, Boston, MA URL http://www.rstudio.com/.
## 
## A BibTeX entry for LaTeX users is
## 
##   @Manual{,
##     title = {RStudio: Integrated Development Environment for R},
##     author = {{RStudio Team}},
##     organization = {RStudio, PBC},
##     address = {Boston, MA},
##     year = {2022},
##     url = {http://www.rstudio.com/},
##   }
```

---

# 3 Preparing data set

## 3.1 Installing and loading packages

Install and load all needed packages.

```
if(!require(pacman, quietly = TRUE)) install.packages("pacman")
p_unload("pacman", negate = TRUE)
p_load("here",         # see the path of the file (Müller, 2020)
       "lattice",      # basics (Sarkar, 2008)
       "survival",     # basics (Therneau, 2022; Therneau & Grambsch, 2000)
       "Formula",      # basics (Zeileis & Croissant, 2010)
       "ggplot2",      # basics (Wickham, 2016)
       "Hmisc",        # basics (Harrell, 2022)
       "pacman",       # basics (Rinker & Kurkiewicz, 2017)
       "dplyr",        # filter variables (Wickham et al., 2022)
       "naniar",       # MCAR (Tierney et al., 2021)
       "haven")        # export sav data (Wickham et al., 2022)
```

## 3.2 Accessing the data (description)

To access the data, set a path (working directory) to a place on your
computer, where the project is located, from where you want to retrieve
documents, or where you want to save documents. For Mac-Users the
function `here` can be used:

```
here("/Users/Anonym/Folder")
## [1] "/Users/Anonym/Folder"
```

Thereafter, check whether your path is working.

```
here()
## [1] "/Users/Anonym/Folder"
```

Import the data you want to work with and assign a variable name to
it.

> *NOTE:*

> - *We only describe the processing of the entire data set
>   (`data1`) and the outlier-corrected data set
>   (`data2`) underlying this manuscript, but do not share them
>   because of the personal data they contain. However, in the following you
>   will be able to download the processed data sets (`data3` and
>   `data4`) where the personal data was deletion. You will find
>   more information in the following descriptions of this R Markdown
>   document.*

> - *You are not able to perform the respective analyses that use the
>   `data1` and `data2` (as the `data1` and
>   `data2` include personal information).*

```
data1 <- haven::read_sav("Data1.sav")
```

View the variable names and column numbers.

```
names(data1)
##  [1] "casenumber1"      "duration"         "age"              "gender"          
##  [5] "profession"       "semester"         "denomination"     "conflict"        
##  [9] "teach"            "belief"           "interest"         "knowledge_sa"    
## [13] "comfort"          "usefulness_sa"    "conscientious_sa" "sport1"          
## [17] "sport2"           "sport3"           "sport4"           "sport5"          
## [21] "sport_mv"         "BI1"              "BI2"              "BI3"             
## [25] "BI4"              "BI"               "AT1"              "AT2"             
## [29] "AT3"              "AT4"              "AT5"              "AT6"             
## [33] "AT7"              "AT8"              "AT9"              "AT"              
## [37] "SN1"              "SN2"              "SN3"              "SN4"             
## [41] "SN"               "PBC1"             "PBC2"             "PBC3"            
## [45] "PBC"              "PRF1"             "PRF2"             "PRF3"            
## [49] "PRF4"             "PRF5"             "PRF6"             "PRF7"            
## [53] "PRF8"             "PRF9"             "PRF10"            "PRF"             
## [57] "PU1"              "PU2"              "PU3"              "PU"              
## [61] "CINS1"            "CINS2"            "CINS3"            "CINS4"           
## [65] "CINS5"            "CINS6"            "CINS7"            "CINS8"           
## [69] "CINS9"            "CINS10"           "CINS11"           "CINS12"          
## [73] "CINS13"           "CINS14"           "CINS15"           "CINS16"          
## [77] "CINS17"           "CINS18"           "CINS19"           "CINS20"          
## [81] "CINS"
```

Define `–99` as missing values in the data frame.

```
data1[data1 == -99] <- NA
```

## 3.3 Excluding outliers (description)

Filter all participants that study to become a teacher for a school
form in which evolution is currently not taught (variable: profession).
Also, filter extreme outliers of the age variable (Ministry for School
and Education of North Rhine-Westphalia [MSB NRW], 2021). Calculate the
proportion of included participants.

```
data2 <- filter(data1, profession != 1, age < 33, na.rm =TRUE)
309/339     # proportion of included participants
## [1] 0.9115044
```

Define `–99` as missing values in the data frame.

```
data2[data2 == -99] <- NA
```

Create a new case number (casenumber2) on basis of the row numbers
and check whether it worked.

```
data2<- dplyr::mutate(data2, casenumber2 = row_number())
names(data2)
##  [1] "casenumber1"      "duration"         "age"              "gender"          
##  [5] "profession"       "semester"         "denomination"     "conflict"        
##  [9] "teach"            "belief"           "interest"         "knowledge_sa"    
## [13] "comfort"          "usefulness_sa"    "conscientious_sa" "sport1"          
## [17] "sport2"           "sport3"           "sport4"           "sport5"          
## [21] "sport_mv"         "BI1"              "BI2"              "BI3"             
## [25] "BI4"              "BI"               "AT1"              "AT2"             
## [29] "AT3"              "AT4"              "AT5"              "AT6"             
## [33] "AT7"              "AT8"              "AT9"              "AT"              
## [37] "SN1"              "SN2"              "SN3"              "SN4"             
## [41] "SN"               "PBC1"             "PBC2"             "PBC3"            
## [45] "PBC"              "PRF1"             "PRF2"             "PRF3"            
## [49] "PRF4"             "PRF5"             "PRF6"             "PRF7"            
## [53] "PRF8"             "PRF9"             "PRF10"            "PRF"             
## [57] "PU1"              "PU2"              "PU3"              "PU"              
## [61] "CINS1"            "CINS2"            "CINS3"            "CINS4"           
## [65] "CINS5"            "CINS6"            "CINS7"            "CINS8"           
## [69] "CINS9"            "CINS10"           "CINS11"           "CINS12"          
## [73] "CINS13"           "CINS14"           "CINS15"           "CINS16"          
## [77] "CINS17"           "CINS18"           "CINS19"           "CINS20"          
## [81] "CINS"             "casenumber2"
```

As new variables will be added in forms of a new column in the back
of the data frame, you might have to rearrange the variables as
desired.

```
data2 <- data2[, c(1, 82, 2:81)]
names(data2)
##  [1] "casenumber1"      "casenumber2"      "duration"         "age"             
##  [5] "gender"           "profession"       "semester"         "denomination"    
##  [9] "conflict"         "teach"            "belief"           "interest"        
## [13] "knowledge_sa"     "comfort"          "usefulness_sa"    "conscientious_sa"
## [17] "sport1"           "sport2"           "sport3"           "sport4"          
## [21] "sport5"           "sport_mv"         "BI1"              "BI2"             
## [25] "BI3"              "BI4"              "BI"               "AT1"             
## [29] "AT2"              "AT3"              "AT4"              "AT5"             
## [33] "AT6"              "AT7"              "AT8"              "AT9"             
## [37] "AT"               "SN1"              "SN2"              "SN3"             
## [41] "SN4"              "SN"               "PBC1"             "PBC2"            
## [45] "PBC3"             "PBC"              "PRF1"             "PRF2"            
## [49] "PRF3"             "PRF4"             "PRF5"             "PRF6"            
## [53] "PRF7"             "PRF8"             "PRF9"             "PRF10"           
## [57] "PRF"              "PU1"              "PU2"              "PU3"             
## [61] "PU"               "CINS1"            "CINS2"            "CINS3"           
## [65] "CINS4"            "CINS5"            "CINS6"            "CINS7"           
## [69] "CINS8"            "CINS9"            "CINS10"           "CINS11"          
## [73] "CINS12"           "CINS13"           "CINS14"           "CINS15"          
## [77] "CINS16"           "CINS17"           "CINS18"           "CINS19"          
## [81] "CINS20"           "CINS"
```

Save the new data frame as `Data2`.

```
write_sav(data2, "Data2.sav")
write.csv(data2,"/Users/Anonym/Folder/Data_csv/Data2.csv")
```

Delete all personal data from the outlier-corrected data set
(`data2`) and safe the data set by applying a new variable
name.

```
data3 <- subset (data2, select = -c(4:8))
names(data3)
##  [1] "casenumber1"      "casenumber2"      "duration"         "conflict"        
##  [5] "teach"            "belief"           "interest"         "knowledge_sa"    
##  [9] "comfort"          "usefulness_sa"    "conscientious_sa" "sport1"          
## [13] "sport2"           "sport3"           "sport4"           "sport5"          
## [17] "sport_mv"         "BI1"              "BI2"              "BI3"             
## [21] "BI4"              "BI"               "AT1"              "AT2"             
## [25] "AT3"              "AT4"              "AT5"              "AT6"             
## [29] "AT7"              "AT8"              "AT9"              "AT"              
## [33] "SN1"              "SN2"              "SN3"              "SN4"             
## [37] "SN"               "PBC1"             "PBC2"             "PBC3"            
## [41] "PBC"              "PRF1"             "PRF2"             "PRF3"            
## [45] "PRF4"             "PRF5"             "PRF6"             "PRF7"            
## [49] "PRF8"             "PRF9"             "PRF10"            "PRF"             
## [53] "PU1"              "PU2"              "PU3"              "PU"              
## [57] "CINS1"            "CINS2"            "CINS3"            "CINS4"           
## [61] "CINS5"            "CINS6"            "CINS7"            "CINS8"           
## [65] "CINS9"            "CINS10"           "CINS11"           "CINS12"          
## [69] "CINS13"           "CINS14"           "CINS15"           "CINS16"          
## [73] "CINS17"           "CINS18"           "CINS19"           "CINS20"          
## [77] "CINS"
```

Save the new data frame as `Data3` in your working
directory. You will need this file for later imputation of the missing
variables.

```
write_sav(data3, "Data3.sav")
```

## 3.4 Accessing the data

This is the point, where **you can start accessing the
data**. You can find the data here. If not done
before, set a path to a place on your computer, where the project is
located, from where you want to retrieve documents, or where you want to
save documents. For Mac-Users the function `here` can be
used:

```
here("/Users/Anonym/Folder")
## [1] "/Users/Anonym/Folder"
```

Thereafter, check whether your path is working.

```
here()
## [1] "/Users/Anonym/Folder"
```

Import the data you want to work with and assign a variable name to
it.

```
data3 <- haven::read_sav("Data3.sav")
```

View the variable names and column numbers.

```
names(data3)
##  [1] "casenumber1"      "casenumber2"      "duration"         "conflict"        
##  [5] "teach"            "belief"           "interest"         "knowledge_sa"    
##  [9] "comfort"          "usefulness_sa"    "conscientious_sa" "sport1"          
## [13] "sport2"           "sport3"           "sport4"           "sport5"          
## [17] "sport_mv"         "BI1"              "BI2"              "BI3"             
## [21] "BI4"              "BI"               "AT1"              "AT2"             
## [25] "AT3"              "AT4"              "AT5"              "AT6"             
## [29] "AT7"              "AT8"              "AT9"              "AT"              
## [33] "SN1"              "SN2"              "SN3"              "SN4"             
## [37] "SN"               "PBC1"             "PBC2"             "PBC3"            
## [41] "PBC"              "PRF1"             "PRF2"             "PRF3"            
## [45] "PRF4"             "PRF5"             "PRF6"             "PRF7"            
## [49] "PRF8"             "PRF9"             "PRF10"            "PRF"             
## [53] "PU1"              "PU2"              "PU3"              "PU"              
## [57] "CINS1"            "CINS2"            "CINS3"            "CINS4"           
## [61] "CINS5"            "CINS6"            "CINS7"            "CINS8"           
## [65] "CINS9"            "CINS10"           "CINS11"           "CINS12"          
## [69] "CINS13"           "CINS14"           "CINS15"           "CINS16"          
## [73] "CINS17"           "CINS18"           "CINS19"           "CINS20"          
## [77] "CINS"
```

## 3.5 Identifying missing values

It is desirable that at least the variables that will be used for
inferential statistical procedures are complete.Start with defining a
new data frame (e.g., `data_MCAR`). Afterwards, search the
data for missing values,´identify missing variables and classify the
nature of it.

```
data_MCAR <- data3[, c(12:77)]
names(data_MCAR)
##  [1] "sport1"   "sport2"   "sport3"   "sport4"   "sport5"   "sport_mv"
##  [7] "BI1"      "BI2"      "BI3"      "BI4"      "BI"       "AT1"     
## [13] "AT2"      "AT3"      "AT4"      "AT5"      "AT6"      "AT7"     
## [19] "AT8"      "AT9"      "AT"       "SN1"      "SN2"      "SN3"     
## [25] "SN4"      "SN"       "PBC1"     "PBC2"     "PBC3"     "PBC"     
## [31] "PRF1"     "PRF2"     "PRF3"     "PRF4"     "PRF5"     "PRF6"    
## [37] "PRF7"     "PRF8"     "PRF9"     "PRF10"    "PRF"      "PU1"     
## [43] "PU2"      "PU3"      "PU"       "CINS1"    "CINS2"    "CINS3"   
## [49] "CINS4"    "CINS5"    "CINS6"    "CINS7"    "CINS8"    "CINS9"   
## [55] "CINS10"   "CINS11"   "CINS12"   "CINS13"   "CINS14"   "CINS15"  
## [61] "CINS16"   "CINS17"   "CINS18"   "CINS19"   "CINS20"   "CINS"
```

Define `–99` as missing values in the data frame.
Thereafter, check the parameters of missing values in your data to be
able to assess their occurrence.

```
data_MCAR[data_MCAR == -99] <- NA
```

```
n_miss(data_MCAR)             # number of missing values/observations
## [1] 8
n_complete(data_MCAR)         # number of complete values/observations
## [1] 20386
pct_miss(data_MCAR)           # percentage of missing values/observations
## [1] 0.03922722
pct_complete(data_MCAR)       # percentage of complete values/observations
## [1] 99.96077
prop_miss(data_MCAR)          # percentage of missing values/observations
## [1] 0.0003922722
prop_complete(data_MCAR)      # percentage of complete values/observation
## [1] 0.9996077

miss_var_summary(data_MCAR)   # summary of percentage of missing values/observations per item
```

```
miss_case_summary(data_MCAR)  # summary of missing values/observation per cases
```

> The test indicates that only a small amount of data was missing
> ( < 2% per item).

## 3.6 Imputing missing values (exkurse to the tool IBM SPSS Statistics)

```
After detecting that a small amount of the data is missing, perform a Little´s missing completely at random (MCAR) test. This is important to classify the nature of missing data (Little, 1988) and for adequate handling of the data, for example when deciding on estimators for the structural equation model (SEM; Enders, 2006, 2013). Analyse the variables that will later be fed into the SEM.

An simple way to perform the test is offered by *IBM SPSS Statistics* (International Business Machines Corporation[IBM], 2021). Therefore, we make a short excursion into IBM SPSS Statistics (version 28.0). This tool supports testing and directly imputing MCAR data by using the expectation-maximum (EM) algorithm with multiple imputations. You can use the following *syntax*:
```

> \* Open the data “Data3”.  
> GET  
> FILE=‘/Users/Anonym/Folder/Data3.sav’.  
> DATASET NAME DataSet3 WINDOW=FRONT.

> \* Perform a Little´s missing completely at random (MCAR) test.  
> MVA VARIABLES=  
> casenumber2 sport1 sport2 sport3 sport4 sport5 sport\_mv  
> BI1 BI2 BI3 BI4 BI  
> AT1 AT2 AT3 AT4 AT5 AT6 AT7 AT8 AT9 AT  
> SN1 SN2 SN3 SN4 SN  
> PBC1 PBC2 PBC3 PBC  
> PRF1 PRF2 PRF3 PRF4 PRF5 PRF6 PRF7 PRF8 PRF9 PRF10 PRF  
> PU1 PU2 PU3 PU  
> CINS1 CINS2 CINS3 CINS4 CINS5 CINS6 CINS7 CINS8 CINS9 CINS10 CINS11
> CINS12  
> CINS13 CINS14 CINS15 CINS16 CINS17 CINS18 CINS19 CINS20 CINS  
> /EM(TOLERANCE=0.001 CONVERGENCE=0.0001 ITERATIONS=25).

> \* Imputation of missings using EM. Note that by doing so SPSS creates
> another data frame that uses only the variables of the following syntax.
> Include the case number so that your key variable remains.  
> DATASET DECLARE data4.  
> MVA VARIABLES=casenumber2 sport1 sport2 sport3 sport4 sport5
> sport\_mv  
> BI1 BI2 BI3 BI4 BI  
> AT1 AT2 AT3 AT4 AT5 AT6 AT7 AT8 AT9 AT  
> SN1 SN2 SN3 SN4 SN  
> PBC1 PBC2 PBC3 PBC  
> PRF1 PRF2 PRF3 PRF4 PRF5 PRF6 PRF7 PRF8 PRF9 PRF10 PRF  
> PU1 PU2 PU3 PU  
> CINS1 CINS2 CINS3 CINS4 CINS5 CINS6 CINS7 CINS8 CINS9 CINS10 CINS11
> CINS12  
> CINS13 CINS14 CINS15 CINS16 CINS17 CINS18 CINS19 CINS20 CINS  
> /EM(TOLERANCE=0.001 CONVERGENCE=0.0001 ITERATIONS=25 OUTFILE=data4).

> DATASET ACTIVATE data4.  
> \* Apply another MCAR test to check whether the imputation worked.  
> MVA VARIABLES=  
> casenumber2 sport1 sport2 sport3 sport4 sport5 sport\_mv  
> BI1 BI2 BI3 BI4 BI  
> AT1 AT2 AT3 AT4 AT5 AT6 AT7 AT8 AT9 AT  
> SN1 SN2 SN3 SN4 SN  
> PBC1 PBC2 PBC3 PBC  
> PRF1 PRF2 PRF3 PRF4 PRF5 PRF6 PRF7 PRF8 PRF9 PRF10 PRF  
> PU1 PU2 PU3 PU  
> CINS1 CINS2 CINS3 CINS4 CINS5 CINS6 CINS7 CINS8 CINS9 CINS10 CINS11
> CINS12  
> CINS13 CINS14 CINS15 CINS16 CINS17 CINS18 CINS19 CINS20 CINS  
> /EM(TOLERANCE=0.001 CONVERGENCE=0.0001 ITERATIONS=25).

> \* Since values of the following scales were imputed for individual
> items, the scale values are adjusted.  
> Compute SN = MEAN(SN1, SN2, SN3, SN4).  
> Execute.

> Compute sport\_mv = MEAN(sport1, sport2, sport3, sport4,
> sport5).  
> Execute.

> Compute CINS = SUM(CINS1, CINS2, CINS3, CINS4, CINS5, CINS6, CINS7,
> CINS8, CINS9,  
> CINS10, CINS11, CINS12, CINS13, CINS14, CINS15, CINS16, CINS17, CINS18,
> CINS19, CINS20).  
> Execute.

> SAVE OUTFILE=‘/Users/Anonym/Folder/data4.sav’  
> /COMPRESSED.

```
data4 <- haven::read_sav("data4.sav")
```

View the variable names and column numbers.

```
names(data4)
##  [1] "casenumber2" "sport1"      "sport2"      "sport3"      "sport4"     
##  [6] "sport5"      "sport_mv"    "BI1"         "BI2"         "BI3"        
## [11] "BI4"         "BI"          "AT1"         "AT2"         "AT3"        
## [16] "AT4"         "AT5"         "AT6"         "AT7"         "AT8"        
## [21] "AT9"         "AT"          "SN1"         "SN2"         "SN3"        
## [26] "SN4"         "SN"          "PBC1"        "PBC2"        "PBC3"       
## [31] "PBC"         "PRF1"        "PRF2"        "PRF3"        "PRF4"       
## [36] "PRF5"        "PRF6"        "PRF7"        "PRF8"        "PRF9"       
## [41] "PRF10"       "PRF"         "PU1"         "PU2"         "PU3"        
## [46] "PU"          "CINS1"       "CINS2"       "CINS3"       "CINS4"      
## [51] "CINS5"       "CINS6"       "CINS7"       "CINS8"       "CINS9"      
## [56] "CINS10"      "CINS11"      "CINS12"      "CINS13"      "CINS14"     
## [61] "CINS15"      "CINS16"      "CINS17"      "CINS18"      "CINS19"     
## [66] "CINS20"      "CINS"
```

Define `–99` as missing values in the data frame.

```
data4[data4 == -99] <- NA
```

## 3.7 Summary

To ensure reuse, the data can also be saved as `.csv`, as
this format is not encrypted, uncompressed, not proprietary/patent and
uses an open, documented standard.

```
write.csv(data3,"/Users/Anonym/Folder/Data_csv/Data3.csv")
write.csv(data4,"/Users/Anonym/Folder/Data_csv/data4.csv")
```

We conclude with 4 data frames:

> Data1 = original data (*N* = 339); *not
> accessible*  
> Data2 = data with removed outliers; needed for descriptive statistics
> (*N* = 309); *not accessible*  
> Data3 = data with removed outliers and deleted personal data (*N*
> = 309); *accessible*  
> **Data4** = short data with removed outliers, deleted
> personal data, and imputed variables **needed for further
> analyses** (e.g., SEM; *N* = 309); *accessible*

---

# 4 Descriptive statistics

We continue in RStudio. Summaries variables that are needed for
further descriptive statistics (DS) in another data frame and view the
data frame.

```
names(data2)
##  [1] "casenumber1"      "casenumber2"      "duration"         "age"             
##  [5] "gender"           "profession"       "semester"         "denomination"    
##  [9] "conflict"         "teach"            "belief"           "interest"        
## [13] "knowledge_sa"     "comfort"          "usefulness_sa"    "conscientious_sa"
## [17] "sport1"           "sport2"           "sport3"           "sport4"          
## [21] "sport5"           "sport_mv"         "BI1"              "BI2"             
## [25] "BI3"              "BI4"              "BI"               "AT1"             
## [29] "AT2"              "AT3"              "AT4"              "AT5"             
## [33] "AT6"              "AT7"              "AT8"              "AT9"             
## [37] "AT"               "SN1"              "SN2"              "SN3"             
## [41] "SN4"              "SN"               "PBC1"             "PBC2"            
## [45] "PBC3"             "PBC"              "PRF1"             "PRF2"            
## [49] "PRF3"             "PRF4"             "PRF5"             "PRF6"            
## [53] "PRF7"             "PRF8"             "PRF9"             "PRF10"           
## [57] "PRF"              "PU1"              "PU2"              "PU3"             
## [61] "PU"               "CINS1"            "CINS2"            "CINS3"           
## [65] "CINS4"            "CINS5"            "CINS6"            "CINS7"           
## [69] "CINS8"            "CINS9"            "CINS10"           "CINS11"          
## [73] "CINS12"           "CINS13"           "CINS14"           "CINS15"          
## [77] "CINS16"           "CINS17"           "CINS18"           "CINS19"          
## [81] "CINS20"           "CINS"
```

```
DS     <- data2[, c(3:16)]         # all variables needed for descriptive statistics
DS_INT <- data2[, c(3,4,7,12:16)]  # interval scaled variables (M, SD, etc.)
DS_NOM <- data2[, c(5,6,8:11)]     # nominal scaled variables (frequencies, percentages)
```

Display the descriptive statistics for interval scaled variables.

```
summary(DS_INT)                    # descriptive statistics
##     duration            age        semester      interest      knowledge_sa  
##  Min.   :    746   Min.   :20   Min.   :1.0   Min.   :2.000   Min.   :1.000  
##  1st Qu.:   2336   1st Qu.:24   1st Qu.:2.0   1st Qu.:6.000   1st Qu.:4.000  
##  Median :   3517   Median :25   Median :3.0   Median :6.000   Median :5.000  
##  Mean   :  23813   Mean   :25   Mean   :2.8   Mean   :6.282   Mean   :4.657  
##  3rd Qu.:   5030   3rd Qu.:26   3rd Qu.:3.0   3rd Qu.:7.000   3rd Qu.:5.000  
##  Max.   :1378945   Max.   :32   Max.   :6.0   Max.   :7.000   Max.   :7.000  
##                                 NA's   :4                                    
##     comfort     usefulness_sa   conscientious_sa
##  Min.   :1.00   Min.   :1.000   Min.   :5.000   
##  1st Qu.:4.00   1st Qu.:4.000   1st Qu.:6.000   
##  Median :5.00   Median :5.000   Median :7.000   
##  Mean   :5.12   Mean   :4.964   Mean   :6.413   
##  3rd Qu.:6.00   3rd Qu.:6.000   3rd Qu.:7.000   
##  Max.   :7.00   Max.   :7.000   Max.   :7.000   
##                                 NA's   :16
```

Adjust the variable duration. The numbers appears to be very high.
Look for outliers and adjust the scaling. Scale the values that are
currently in *seconds* format to *minutes* format
(conversion from seconds to minutes:
`y minutes = x seconds / 60` ).

```
duration_out_rm <- data2$duration[!data2$duration %in% boxplot.stats(data2$duration)$out]

duration_min <- duration_out_rm/60
summary(duration_min)
##    Min. 1st Qu.  Median    Mean 3rd Qu.    Max. 
##   12.43   37.00   51.45   56.15   70.52  128.03
```

```
sd(duration_min, na.rm=TRUE)
## [1] 23.49955
sd(data2$age)
## [1] 2.120552
sd(data2$semester, na.rm = TRUE)
## [1] 0.9780485
sd(data2$interest)
## [1] 0.8188225
sd(data2$knowledge_sa)
## [1] 1.208248
sd(data2$comfort)
## [1] 1.297562
sd(data2$usefulness_sa)
## [1] 1.375349
sd(data2$conscientious_sa, na.rm = TRUE)
## [1] 0.680025
```

Display the descriptive statistics for nominal scaled variables. You
can use the function `tabyl` or the function
`questionr::freq`. We used the later one. It is similar to
the output of IBM SPSS Statistics.

```
questionr::freq(DS$gender, cum = TRUE, total = TRUE)        # category description, frequency, (valid,cumulative) percentage
```

```
questionr::freq(DS$denomination, cum = TRUE, total = TRUE)  # category description, frequency, (valid,cumulative) percentage
```

```
questionr::freq(DS$conflict, cum = TRUE, total = TRUE)      # category description, frequency, (valid,cumulative) percentage
```

```
questionr::freq(DS$teach, cum = TRUE, total = TRUE)         # category description, frequency, (valid,cumulative) percentage
```

```
questionr::freq(DS$belief, cum = TRUE, total = TRUE)        # category description, frequency, (valid,cumulative) percentage
```

```
questionr::freq(DS$profession, cum = TRUE, total = TRUE)    # category description, frequency, (valid,cumulative) percentage
```

---

# 5 Preliminary analysis

Check the preconditions of the data (Fox, 2019; Korkmaz et al., 2014;
Neter et al., 1990).

## 5.1 Installing and loading packages

Install and load all needed packages.

```
p_load("psych",                 # describe, reliability (Revelle, 2022)
       "car",                   # vif (John & Sanford, 2019)
       "janitor",               # tabyl, frequencies, percentages (Firke, 2021)
       "questionr",             # tabyl, frequencies, percentages, similar to IBM SPSS Statistics output (Barnier et al., 2022)
       "MVN",                   # test for multivariate normal distribution, madiaTest (Korkmaz etal., 2014)
       "lmtest",                # multiple regression, Durbin Watson test (Achim Zeileis & Torsten Hothorn, 2002)
       "carData",               # is needed to load the package "car" (Fox et al., 2022)
       "corrplot",              # correlation plot (Wei & Simko, 2021)
       "apaTables",             # visualization of the correlation as APA table (Stanley, 2021)
       "PerformanceAnalytics",  # visualization of the correlation (Peterson & Carl, 2020)
       "RColorBrewer",          # changing colors of the colorful correlation plot (Neuwirth, 2022)
       "lavaan")                # description of the CINS for reliability (Rosseel, 2012)
```

## 5.2 Preparing variables for analyses

View data set to identify the variables that you need for further
analyses.

```
names(data4)
##  [1] "casenumber2" "sport1"      "sport2"      "sport3"      "sport4"     
##  [6] "sport5"      "sport_mv"    "BI1"         "BI2"         "BI3"        
## [11] "BI4"         "BI"          "AT1"         "AT2"         "AT3"        
## [16] "AT4"         "AT5"         "AT6"         "AT7"         "AT8"        
## [21] "AT9"         "AT"          "SN1"         "SN2"         "SN3"        
## [26] "SN4"         "SN"          "PBC1"        "PBC2"        "PBC3"       
## [31] "PBC"         "PRF1"        "PRF2"        "PRF3"        "PRF4"       
## [36] "PRF5"        "PRF6"        "PRF7"        "PRF8"        "PRF9"       
## [41] "PRF10"       "PRF"         "PU1"         "PU2"         "PU3"        
## [46] "PU"          "CINS1"       "CINS2"       "CINS3"       "CINS4"      
## [51] "CINS5"       "CINS6"       "CINS7"       "CINS8"       "CINS9"      
## [56] "CINS10"      "CINS11"      "CINS12"      "CINS13"      "CINS14"     
## [61] "CINS15"      "CINS16"      "CINS17"      "CINS18"      "CINS19"     
## [66] "CINS20"      "CINS"
```

Summaries variables that are needed for further analyses in another
data frame and view the data frame.

```
variables <- data4[, c(12,22,27,31,42,46,67,7)]
```

Show names of the variables.

```
names(variables)
## [1] "BI"       "AT"       "SN"       "PBC"      "PRF"      "PU"       "CINS"    
## [8] "sport_mv"
```

## 5.3 Conducting preliminary analyses

### 5.3.1 Linear relationships between the variables

Test whether *linear relationships* exist between the
variables.

```
pairs(variables, panel=panel.smooth)
```

> The relations between the variables are approximately linear.

### 5.3.2 Normality of the distribution

Test *univariate and multivariate normal distribution*.

```
## $multivariateNormality
##              Test        Statistic              p value Result
## 1 Mardia Skewness 423.511636847357 1.81400667290854e-35     NO
## 2 Mardia Kurtosis  5.7841998611047 7.28582794096155e-09     NO
## 3             MVN             <NA>                 <NA>     NO
## 
## $univariateNormality
##           Test  Variable Statistic   p value Normality
## 1 Shapiro-Wilk    BI        0.8888  <0.001      NO    
## 2 Shapiro-Wilk    AT        0.9007  <0.001      NO    
## 3 Shapiro-Wilk    SN        0.8657  <0.001      NO    
## 4 Shapiro-Wilk    PBC       0.9741  <0.001      NO    
## 5 Shapiro-Wilk    PRF       0.9251  <0.001      NO    
## 6 Shapiro-Wilk    PU        0.9311  <0.001      NO    
## 7 Shapiro-Wilk   CINS       0.9577  <0.001      NO    
## 8 Shapiro-Wilk sport_mv     0.8991  <0.001      NO    
## 
## $Descriptives
##            n     Mean   Std.Dev   Median      Min Max     25th     75th
## BI       309 6.027508 0.9128256 6.250000 2.000000   7 5.500000 6.750000
## AT       309 6.134484 0.7638237 6.333333 3.222222   7 5.777778 6.777778
## SN       309 5.952265 0.9987091 6.250000 3.250000   7 5.500000 6.750000
## PBC      309 4.980583 1.0654876 5.000000 1.333333   7 4.000000 5.666667
## PRF      309 3.469644 1.6793649 3.220000 1.400000   7 1.820000 4.620000
## PU       309 4.308522 1.2098215 4.333333 1.000000   7 4.000000 5.000000
## CINS     309 4.629288 1.2835277 4.900000 1.750000   7 3.500000 5.600000
## sport_mv 309 5.662783 1.1806087 6.000000 1.200000   7 5.000000 6.600000
##                Skew   Kurtosis
## BI       -1.1688622  1.4988175
## AT       -1.1396742  1.2038620
## SN       -0.9028287 -0.3813557
## PBC      -0.1155383 -0.4356657
## PRF       0.4596648 -0.8664683
## PU       -0.4702225  1.0224327
## CINS     -0.3189503 -0.9066464
## sport_mv -1.0998350  0.8449813
```

> Not all variables are normally distributed.

### 5.3.3 Multicollinearity and autocorrelation

Define the regression models underlying our structural equation model
to check whether the data show *multicollinearity* and
*autocorrelation*.

Check the multicollinearity by means of the *variance inflation
factor (VIF)*.

```
##       AT       SN      PBC     CINS 
## 1.553946 1.663776 1.182521 1.130854
##      PRF       PU       SN 
## 1.063210 1.072211 1.128122
```

> The data show no thread to multicollinearity as the VIF scores were
> below the recommended threshold of < 10 (Kline, 2015; Neter et al.,
> 1990).

Check the autocorrelation by means of the *Durbin-Watson
test*.

```
## 
##  Durbin-Watson test
## 
## data:  V1
## DW = 2.072, p-value = 0.7355
## alternative hypothesis: true autocorrelation is greater than 0
## 
##  Durbin-Watson test
## 
## data:  V2
## DW = 1.7385, p-value = 0.01027
## alternative hypothesis: true autocorrelation is greater than 0
## 
##  Durbin-Watson test
## 
## data:  V3
## DW = 2.1002, p-value = 0.8129
## alternative hypothesis: true autocorrelation is greater than 0
```

> The data show no autocorrelation as the results of the Durbin Watson
> tests were close to the recommended value of 2 (Kline, 2015; Neter et
> al., 1990).

### 5.3.4 Checking the correlations

Build the correlation as matrix, save in the variable *res*,
and visualize it.

```
##             BI    AT    SN  PBC   PRF    PU  CINS sport_mv
## BI        1.00  0.67  0.60 0.44 -0.17  0.31  0.37     0.13
## AT        0.67  1.00  0.58 0.32 -0.28  0.36  0.25     0.10
## SN        0.60  0.58  1.00 0.36 -0.24  0.26  0.32     0.09
## PBC       0.44  0.32  0.36 1.00  0.00  0.20  0.20     0.10
## PRF      -0.17 -0.28 -0.24 0.00  1.00 -0.10 -0.17     0.02
## PU        0.31  0.36  0.26 0.20 -0.10  1.00  0.11     0.14
## CINS      0.37  0.25  0.32 0.20 -0.17  0.11  1.00     0.10
## sport_mv  0.13  0.10  0.09 0.10  0.02  0.14  0.10     1.00
## 
## n= 309 
## 
## 
## P
##          BI     AT     SN     PBC    PRF    PU     CINS   sport_mv
## BI              0.0000 0.0000 0.0000 0.0034 0.0000 0.0000 0.0192  
## AT       0.0000        0.0000 0.0000 0.0000 0.0000 0.0000 0.0714  
## SN       0.0000 0.0000        0.0000 0.0000 0.0000 0.0000 0.1169  
## PBC      0.0000 0.0000 0.0000        0.9845 0.0004 0.0006 0.0749  
## PRF      0.0034 0.0000 0.0000 0.9845        0.0934 0.0024 0.6916  
## PU       0.0000 0.0000 0.0000 0.0004 0.0934        0.0605 0.0122  
## CINS     0.0000 0.0000 0.0000 0.0006 0.0024 0.0605        0.0947  
## sport_mv 0.0192 0.0714 0.1169 0.0749 0.6916 0.0122 0.0947
```

If you need more than 2 decimal places, extract the correlation
coefficients.

```
##                  BI         AT         SN         PBC          PRF         PU
## BI        1.0000000  0.6694950  0.6028784 0.442585003 -0.165945830  0.3122549
## AT        0.6694950  1.0000000  0.5823527 0.321927928 -0.275724230  0.3629062
## SN        0.6028784  0.5823527  1.0000000 0.360939666 -0.241346675  0.2572039
## PBC       0.4425850  0.3219279  0.3609397 1.000000000  0.001109015  0.2011225
## PRF      -0.1659458 -0.2757242 -0.2413467 0.001109015  1.000000000 -0.0956034
## PU        0.3122549  0.3629062  0.2572039 0.201122495 -0.095603398  1.0000000
## CINS      0.3693593  0.2498848  0.3233537 0.195012855 -0.171882349  0.1068938
## sport_mv  0.1332102  0.1026987  0.0893798 0.101460510  0.022652549  0.1424816
##                 CINS   sport_mv
## BI        0.36935934 0.13321021
## AT        0.24988481 0.10269869
## SN        0.32335370 0.08937980
## PBC       0.19501286 0.10146051
## PRF      -0.17188235 0.02265255
## PU        0.10689377 0.14248157
## CINS      1.00000000 0.09524168
## sport_mv  0.09524168 1.00000000
## NULL
```

You can also visualize the correlation in APA style. This style has
the advantage that the mean values (*M*) and standard deviations
(*SD*) are displayed automatically.

```
## 
## 
## Table 1 
## 
## Means, standard deviations, and correlations with confidence intervals
##  
## 
##   Variable    M    SD   1            2            3            4          
##   1. BI       6.03 0.91                                                   
##                                                                           
##   2. AT       6.13 0.76 .67**                                             
##                         [.60, .73]                                        
##                                                                           
##   3. SN       5.95 1.00 .60**        .58**                                
##                         [.53, .67]   [.50, .65]                           
##                                                                           
##   4. PBC      4.98 1.07 .44**        .32**        .36**                   
##                         [.35, .53]   [.22, .42]   [.26, .45]              
##                                                                           
##   5. PRF      3.47 1.68 -.17**       -.28**       -.24**       .00        
##                         [-.27, -.06] [-.38, -.17] [-.34, -.13] [-.11, .11]
##                                                                           
##   6. PU       4.31 1.21 .31**        .36**        .26**        .20**      
##                         [.21, .41]   [.26, .46]   [.15, .36]   [.09, .31] 
##                                                                           
##   7. CINS     4.63 1.28 .37**        .25**        .32**        .20**      
##                         [.27, .46]   [.14, .35]   [.22, .42]   [.09, .30] 
##                                                                           
##   8. sport_mv 5.66 1.18 .13*         .10          .09          .10        
##                         [.02, .24]   [-.01, .21]  [-.02, .20]  [-.01, .21]
##                                                                           
##   5            6           7          
##                                       
##                                       
##                                       
##                                       
##                                       
##                                       
##                                       
##                                       
##                                       
##                                       
##                                       
##                                       
##                                       
##                                       
##   -.10                                
##   [-.20, .02]                         
##                                       
##   -.17**       .11                    
##   [-.28, -.06] [-.00, .22]            
##                                       
##   .02          .14*        .10        
##   [-.09, .13]  [.03, .25]  [-.02, .20]
##                                       
## 
## Note. M and SD are used to represent mean and standard deviation, respectively.
## Values in square brackets indicate the 95% confidence interval.
## The confidence interval is a plausible range of population correlations 
## that could have caused the sample correlation (Cumming, 2014).
##  * indicates p < .05. ** indicates p < .01.
##
```

NOTE: All variables were scaled to a 7-point Likert scale. However,
the CINS is regularly on a 0–20 point scale. Therefore, we need to
convert the mean and standard deviation back to the real scale before
interpreting. This means that we have to apply the following formula to
the *M* and the *SD*: `x / 7 * 20`.

```
      4.63/7*20
## [1] 13.22857
      1.28/7*20
## [1] 3.657143
```

Before the validation of the measurement model, we tested the
reliability of the variable CINS. Due to its nature the CINS was not
required for validating the measurement model. It consists of 20
dichotomously rated items and is thus included as sum score in the later
SEM.

Calculate the Cronbach´s \(a\). It
should exceed the threshold of \(a\) > .7 (Fornell & Larcker, 1981;
Kline, 2015). For the calculated values of *M* and *SD*
see above.

```
## Some items ( CINS4 ) were negatively correlated with the total scale and 
## probably should be reversed.  
## To do this, run the function again with the 'check.keys=TRUE' option
## 
## Reliability analysis   
## Call: alpha(x = subset(data4, select = c(CINS1, CINS2, CINS3, CINS4, 
##     CINS5, CINS6, CINS7, CINS8, CINS9, CINS10, CINS11, CINS12, 
##     CINS13, CINS14, CINS15, CINS16, CINS17, CINS18, CINS19, CINS20)))
## 
##   raw_alpha std.alpha G6(smc) average_r S/N   ase mean    sd median_r
##       0.73      0.73    0.76      0.12 2.7 0.021 0.23 0.064     0.13
## 
##     95% confidence boundaries 
##          lower alpha upper
## Feldt     0.69  0.73  0.78
## Duhachek  0.69  0.73  0.78
## 
##  Reliability if an item is dropped:
##        raw_alpha std.alpha G6(smc) average_r S/N alpha se  var.r med.r
## CINS1       0.73      0.73    0.76      0.12 2.7    0.021 0.0148  0.13
## CINS2       0.73      0.72    0.75      0.12 2.6    0.022 0.0155  0.13
## CINS3       0.73      0.73    0.76      0.12 2.7    0.022 0.0153  0.13
## CINS4       0.77      0.77    0.78      0.15 3.3    0.019 0.0083  0.14
## CINS5       0.72      0.72    0.75      0.12 2.5    0.022 0.0154  0.12
## CINS6       0.71      0.70    0.73      0.11 2.4    0.024 0.0135  0.12
## CINS7       0.72      0.72    0.75      0.12 2.6    0.022 0.0148  0.13
## CINS8       0.71      0.71    0.74      0.11 2.4    0.023 0.0146  0.12
## CINS9       0.73      0.73    0.76      0.12 2.7    0.022 0.0157  0.13
## CINS10      0.73      0.72    0.75      0.12 2.6    0.022 0.0153  0.13
## CINS11      0.72      0.72    0.75      0.12 2.5    0.022 0.0152  0.12
## CINS12      0.73      0.72    0.75      0.12 2.6    0.022 0.0156  0.13
## CINS13      0.72      0.71    0.74      0.12 2.5    0.023 0.0146  0.12
## CINS14      0.72      0.72    0.75      0.12 2.6    0.022 0.0156  0.13
## CINS15      0.71      0.71    0.74      0.11 2.4    0.023 0.0146  0.12
## CINS16      0.73      0.73    0.75      0.12 2.6    0.022 0.0156  0.13
## CINS17      0.72      0.71    0.74      0.12 2.5    0.023 0.0144  0.12
## CINS18      0.72      0.72    0.75      0.12 2.5    0.022 0.0151  0.12
## CINS19      0.71      0.70    0.73      0.11 2.4    0.024 0.0134  0.12
## CINS20      0.71      0.70    0.74      0.11 2.4    0.023 0.0138  0.12
## 
##  Item statistics 
##          n raw.r std.r r.cor r.drop  mean   sd
## CINS1  309  0.30  0.29  0.22   0.18 0.241 0.16
## CINS2  309  0.36  0.37  0.31   0.26 0.271 0.15
## CINS3  309  0.29  0.33  0.25   0.21 0.313 0.11
## CINS4  309 -0.18 -0.17 -0.32  -0.29 0.095 0.16
## CINS5  309  0.44  0.44  0.39   0.34 0.258 0.15
## CINS6  309  0.58  0.58  0.57   0.49 0.223 0.17
## CINS7  309  0.43  0.42  0.37   0.32 0.249 0.16
## CINS8  309  0.52  0.51  0.48   0.41 0.211 0.17
## CINS9  309  0.34  0.33  0.25   0.22 0.123 0.17
## CINS10 309  0.38  0.38  0.32   0.27 0.240 0.16
## CINS11 309  0.43  0.43  0.39   0.32 0.258 0.15
## CINS12 309  0.37  0.38  0.32   0.26 0.234 0.16
## CINS13 309  0.49  0.48  0.44   0.37 0.198 0.17
## CINS14 309  0.40  0.40  0.33   0.29 0.246 0.16
## CINS15 309  0.52  0.53  0.50   0.43 0.272 0.15
## CINS16 309  0.31  0.34  0.27   0.22 0.304 0.12
## CINS17 309  0.47  0.46  0.42   0.36 0.186 0.17
## CINS18 309  0.45  0.44  0.39   0.34 0.240 0.16
## CINS19 309  0.59  0.59  0.58   0.50 0.244 0.16
## CINS20 309  0.57  0.56  0.55   0.47 0.223 0.17
## 
## Non missing response frequency for each item
##           0 2.00530228952611e-05 3.7625904318439e-05 0.35 miss
## CINS1  0.31                    0                   0 0.69    0
## CINS2  0.23                    0                   0 0.77    0
## CINS3  0.11                    0                   0 0.89    0
## CINS4  0.72                    0                   0 0.27    0
## CINS5  0.26                    0                   0 0.74    0
## CINS6  0.36                    0                   0 0.64    0
## CINS7  0.29                    0                   0 0.71    0
## CINS8  0.40                    0                   0 0.60    0
## CINS9  0.65                    0                   0 0.35    0
## CINS10 0.31                    0                   0 0.69    0
## CINS11 0.26                    0                   0 0.74    0
## CINS12 0.33                    0                   0 0.67    0
## CINS13 0.43                    0                   0 0.57    0
## CINS14 0.30                    0                   0 0.70    0
## CINS15 0.22                    0                   0 0.78    0
## CINS16 0.13                    0                   0 0.87    0
## CINS17 0.47                    0                   0 0.53    0
## CINS18 0.31                    0                   0 0.69    0
## CINS19 0.30                    0                   0 0.70    0
## CINS20 0.36                    0                   0 0.64    0
```

> Cronbach’s \(a\) = .73,
> *M* = 13.23, *SD* = 3.66.

---

# 6 Measurement model and validation

## 6.1 Installing and loading packages

Install and load all needed packages.

```
p_load("GPArotation",   # reliability (Bernaards et al., 2005)
       "semTools",      # reliability (Jorgensen et al., 2022)
       "EFAtools")      # Harman’s single-factor analysis ( Steiner & Grieder, 2020)
```

## 6.2 Analyzing the probability of common method bias

Perform Harman’s single-factor to reveal the probability of common
method bias in the conceptualized measurement model (Podsakoff et al.,
2003).

```
## ℹ 'x' was not a correlation matrix. Correlations are found from entered raw data.
## 
## EFA performed with type = 'EFAtools', method = 'PAF', and rotation = 'none'.
## 
## ── Unrotated Loadings ──────────────────────────────────────────────────────────
## 
##       F1  
## BI    .832
## AT    .809
## SN    .731
## PBC   .462
## PRF  -.255
## PU    .398
## 
## ── Variances Accounted for ─────────────────────────────────────────────────────
## 
##               F1  
## SS loadings   2.318
## Prop Tot Var  0.386
## 
## ── Model Fit ───────────────────────────────────────────────────────────────────
## 
## CAF: .48
## df:   9
```

> The maximum variance of the model that is explained by one variable
> is only **38.6%** (< 50%) indicating no major threat to
> common method bias.

Additionally, refer back to the correlation matrix `res`
and the applied marker variable `sport_mv`.

```
##             BI    AT    SN  PBC   PRF    PU  CINS sport_mv
## BI        1.00  0.67  0.60 0.44 -0.17  0.31  0.37     0.13
## AT        0.67  1.00  0.58 0.32 -0.28  0.36  0.25     0.10
## SN        0.60  0.58  1.00 0.36 -0.24  0.26  0.32     0.09
## PBC       0.44  0.32  0.36 1.00  0.00  0.20  0.20     0.10
## PRF      -0.17 -0.28 -0.24 0.00  1.00 -0.10 -0.17     0.02
## PU        0.31  0.36  0.26 0.20 -0.10  1.00  0.11     0.14
## CINS      0.37  0.25  0.32 0.20 -0.17  0.11  1.00     0.10
## sport_mv  0.13  0.10  0.09 0.10  0.02  0.14  0.10     1.00
## 
## n= 309 
## 
## 
## P
##          BI     AT     SN     PBC    PRF    PU     CINS   sport_mv
## BI              0.0000 0.0000 0.0000 0.0034 0.0000 0.0000 0.0192  
## AT       0.0000        0.0000 0.0000 0.0000 0.0000 0.0000 0.0714  
## SN       0.0000 0.0000        0.0000 0.0000 0.0000 0.0000 0.1169  
## PBC      0.0000 0.0000 0.0000        0.9845 0.0004 0.0006 0.0749  
## PRF      0.0034 0.0000 0.0000 0.9845        0.0934 0.0024 0.6916  
## PU       0.0000 0.0000 0.0000 0.0004 0.0934        0.0605 0.0122  
## CINS     0.0000 0.0000 0.0000 0.0006 0.0024 0.0605        0.0947  
## sport_mv 0.0192 0.0714 0.1169 0.0749 0.6916 0.0122 0.0947
```

> The correlation shows no to minor correlations between the marker
> variable and the other variables, strengthening the case against common
> method bias (Semin et al., 2005; Simmering et al., 2014).

## 6.3 Defining the measurement model

Define the measurement model (MM). Assign a new name to the latent
variables, so that you have unique variable names. If the latent
variables have the same name as the variables in your data an error
messages might arise (e.g., suffix *MM*).

---

Before running the confirmatory factor analysis for the defined
measurement model, decide which estimator is most appropriate one for
the nature of the data (e.g., non-normally distributed). Different
estimators are presented in the package description for lavaan.

Many estimators have *robust* variants. We chose the robust
variant of the maximum likelihood estimator `MLM` (Kamel
& Guillaume, 2019; Rosseel, 2012, Satorra & Bentler, 2001).

`"MLM"` = “maximum likelihood estimation with robust
standard errors and a Satorra-Bentler scaled test statistic. For
complete data only.” (see https://lavaan.ugent.be/tutorial/est.html)

[However, if you did not impute the missings and values are
*missing at random (MAR)* or *missing completely at random
(MCAR)*, and cases with incomplete data are still to be included in
the analyses, then you could, for example, use the`MLR`
estimator and use the “full information maximum likelihood” (FIML)
method to estimate your model (e.g., Arbuckle et al., 1996; Enders,
2013; Grund, 2017; Rosseel, 2012, Yuan & Bentler, 2000). To do so
use the command `missing = "FIML"`.]

---

Run the confirmatory analysis.

Save the summary of the model fit in a new variable.

## 6.4 Model fit and loadings

View the summary of model fit and loadings (\(\lambda\)) and save the indices in a new
variable. The loadings for each item should be at least \(\lambda\) > .5 to signify indicator
reliability (Chin, 1998; Hair et al., 2010).

```
## lavaan 0.6-11 ended normally after 68 iterations
## 
##   Estimator                                         ML
##   Optimization method                           NLMINB
##   Number of model parameters                        81
##                                                       
##   Number of observations                           309
##                                                       
## Model Test User Model:
##                                               Standard      Robust
##   Test Statistic                              1049.690     902.003
##   Degrees of freedom                               480         480
##   P-value (Chi-square)                           0.000       0.000
##   Scaling correction factor                                  1.164
##        Satorra-Bentler correction                                 
## 
## Model Test Baseline Model:
## 
##   Test statistic                              8070.116    6208.245
##   Degrees of freedom                               528         528
##   P-value                                        0.000       0.000
##   Scaling correction factor                                  1.300
## 
## User Model versus Baseline Model:
## 
##   Comparative Fit Index (CFI)                    0.924       0.926
##   Tucker-Lewis Index (TLI)                       0.917       0.918
##                                                                   
##   Robust Comparative Fit Index (CFI)                         0.933
##   Robust Tucker-Lewis Index (TLI)                            0.927
## 
## Loglikelihood and Information Criteria:
## 
##   Loglikelihood user model (H0)             -13833.416  -13833.416
##   Loglikelihood unrestricted model (H1)             NA          NA
##                                                                   
##   Akaike (AIC)                               27828.831   27828.831
##   Bayesian (BIC)                             28131.232   28131.232
##   Sample-size adjusted Bayesian (BIC)        27874.332   27874.332
## 
## Root Mean Square Error of Approximation:
## 
##   RMSEA                                          0.062       0.053
##   90 Percent confidence interval - lower         0.057       0.048
##   90 Percent confidence interval - upper         0.067       0.058
##   P-value RMSEA <= 0.05                          0.000       0.133
##                                                                   
##   Robust RMSEA                                               0.058
##   90 Percent confidence interval - lower                     0.052
##   90 Percent confidence interval - upper                     0.063
## 
## Standardized Root Mean Square Residual:
## 
##   SRMR                                           0.054       0.054
## 
## Parameter Estimates:
## 
##   Standard errors                           Robust.sem
##   Information                                 Expected
##   Information saturated (h1) model          Structured
## 
## Latent Variables:
##                    Estimate  Std.Err  z-value  P(>|z|) ci.lower ci.upper
##   BI_MM =~                                                              
##     BI1               1.000                               1.000    1.000
##     BI2               1.191    0.075   15.908    0.000    1.044    1.337
##     BI3               0.825    0.103    8.032    0.000    0.624    1.027
##     BI4               0.562    0.084    6.675    0.000    0.397    0.726
##   AT_MM =~                                                              
##     AT1               1.000                               1.000    1.000
##     AT2               0.910    0.063   14.403    0.000    0.787    1.034
##     AT3               0.862    0.120    7.162    0.000    0.626    1.097
##     AT4               1.092    0.081   13.437    0.000    0.932    1.251
##     AT5               1.104    0.103   10.675    0.000    0.902    1.307
##     AT6               1.134    0.101   11.179    0.000    0.935    1.333
##     AT7               1.272    0.110   11.579    0.000    1.057    1.488
##     AT8               1.208    0.087   13.874    0.000    1.037    1.378
##     AT9               1.167    0.089   13.179    0.000    0.993    1.340
##   SN_MM =~                                                              
##     SN1               1.000                               1.000    1.000
##     SN2               0.920    0.053   17.501    0.000    0.817    1.023
##     SN3               0.650    0.068    9.591    0.000    0.517    0.782
##     SN4               0.710    0.051   13.816    0.000    0.609    0.810
##   PBC_MM =~                                                             
##     PBC1              1.000                               1.000    1.000
##     PBC2              0.846    0.117    7.254    0.000    0.618    1.075
##     PBC3              0.939    0.095    9.897    0.000    0.753    1.125
##   PRF_MM =~                                                             
##     PRF1              1.000                               1.000    1.000
##     PRF2              0.981    0.028   34.944    0.000    0.926    1.036
##     PRF3              0.846    0.033   25.677    0.000    0.782    0.911
##     PRF4              0.925    0.030   31.143    0.000    0.866    0.983
##     PRF5              0.771    0.041   19.026    0.000    0.692    0.851
##     PRF6              0.676    0.046   14.777    0.000    0.587    0.766
##     PRF7              0.876    0.035   24.885    0.000    0.807    0.945
##     PRF8              0.838    0.040   21.166    0.000    0.760    0.915
##     PRF9              0.891    0.035   25.334    0.000    0.822    0.960
##     PRF10             0.884    0.038   23.416    0.000    0.810    0.958
##   PU_MM =~                                                              
##     PU1               1.000                               1.000    1.000
##     PU2               0.888    0.043   20.426    0.000    0.802    0.973
##     PU3               0.912    0.040   22.818    0.000    0.833    0.990
##    Std.lv  Std.all
##                   
##     0.924    0.860
##     1.100    0.787
##     0.762    0.639
##     0.519    0.605
##                   
##     0.670    0.790
##     0.610    0.725
##     0.577    0.514
##     0.732    0.773
##     0.740    0.638
##     0.760    0.810
##     0.853    0.791
##     0.809    0.867
##     0.782    0.767
##                   
##     1.120    0.905
##     1.030    0.856
##     0.727    0.587
##     0.795    0.661
##                   
##     1.013    0.883
##     0.857    0.583
##     0.951    0.766
##                   
##     1.889    0.895
##     1.852    0.917
##     1.598    0.869
##     1.746    0.873
##     1.456    0.784
##     1.278    0.630
##     1.655    0.846
##     1.582    0.878
##     1.682    0.803
##     1.669    0.883
##                   
##     1.242    0.934
##     1.102    0.841
##     1.132    0.896
## 
## Covariances:
##                    Estimate  Std.Err  z-value  P(>|z|) ci.lower ci.upper
##   BI_MM ~~                                                              
##     AT_MM             0.479    0.089    5.382    0.000    0.304    0.653
##     SN_MM             0.698    0.093    7.536    0.000    0.517    0.880
##     PBC_MM            0.408    0.064    6.360    0.000    0.282    0.533
##     PRF_MM           -0.359    0.128   -2.811    0.005   -0.609   -0.109
##     PU_MM             0.422    0.096    4.415    0.000    0.235    0.609
##   AT_MM ~~                                                              
##     SN_MM             0.491    0.076    6.439    0.000    0.342    0.641
##     PBC_MM            0.195    0.044    4.482    0.000    0.110    0.281
##     PRF_MM           -0.376    0.098   -3.833    0.000   -0.568   -0.184
##     PU_MM             0.305    0.072    4.231    0.000    0.164    0.447
##   SN_MM ~~                                                              
##     PBC_MM            0.409    0.078    5.221    0.000    0.255    0.562
##     PRF_MM           -0.594    0.142   -4.193    0.000   -0.872   -0.316
##     PU_MM             0.400    0.096    4.144    0.000    0.211    0.589
##   PBC_MM ~~                                                             
##     PRF_MM            0.019    0.122    0.155    0.877   -0.219    0.257
##     PU_MM             0.288    0.092    3.123    0.002    0.107    0.469
##   PRF_MM ~~                                                             
##     PU_MM            -0.218    0.160   -1.358    0.174   -0.531    0.096
##    Std.lv  Std.all
##                   
##     0.774    0.774
##     0.675    0.675
##     0.436    0.436
##    -0.206   -0.206
##     0.368    0.368
##                   
##     0.655    0.655
##     0.288    0.288
##    -0.297   -0.297
##     0.367    0.367
##                   
##     0.360    0.360
##    -0.281   -0.281
##     0.287    0.287
##                   
##     0.010    0.010
##     0.229    0.229
##                   
##    -0.093   -0.093
## 
## Variances:
##                    Estimate  Std.Err  z-value  P(>|z|) ci.lower ci.upper
##    .BI1               0.300    0.050    6.052    0.000    0.203    0.397
##    .BI2               0.743    0.098    7.573    0.000    0.551    0.936
##    .BI3               0.842    0.128    6.586    0.000    0.591    1.092
##    .BI4               0.466    0.066    7.083    0.000    0.337    0.595
##    .AT1               0.271    0.035    7.843    0.000    0.203    0.339
##    .AT2               0.336    0.047    7.083    0.000    0.243    0.429
##    .AT3               0.930    0.091   10.183    0.000    0.751    1.110
##    .AT4               0.360    0.052    6.973    0.000    0.259    0.461
##    .AT5               0.798    0.067   11.921    0.000    0.667    0.929
##    .AT6               0.303    0.041    7.310    0.000    0.222    0.384
##    .AT7               0.436    0.066    6.642    0.000    0.307    0.565
##    .AT8               0.216    0.039    5.548    0.000    0.140    0.292
##    .AT9               0.427    0.052    8.154    0.000    0.324    0.530
##    .SN1               0.276    0.051    5.373    0.000    0.176    0.377
##    .SN2               0.388    0.086    4.504    0.000    0.219    0.557
##    .SN3               1.008    0.173    5.824    0.000    0.669    1.347
##    .SN4               0.813    0.105    7.715    0.000    0.606    1.019
##    .PBC1              0.289    0.083    3.487    0.000    0.127    0.452
##    .PBC2              1.430    0.170    8.418    0.000    1.097    1.763
##    .PBC3              0.636    0.129    4.926    0.000    0.383    0.889
##    .PRF1              0.885    0.124    7.147    0.000    0.642    1.127
##    .PRF2              0.649    0.122    5.336    0.000    0.411    0.887
##    .PRF3              0.825    0.103    8.048    0.000    0.624    1.026
##    .PRF4              0.952    0.121    7.899    0.000    0.716    1.189
##    .PRF5              1.331    0.172    7.748    0.000    0.994    1.668
##    .PRF6              2.477    0.202   12.242    0.000    2.080    2.873
##    .PRF7              1.084    0.124    8.728    0.000    0.841    1.328
##    .PRF8              0.744    0.087    8.579    0.000    0.574    0.913
##    .PRF9              1.560    0.179    8.710    0.000    1.209    1.911
##    .PRF10             0.791    0.113    7.020    0.000    0.570    1.011
##    .PU1               0.225    0.060    3.745    0.000    0.107    0.343
##    .PU2               0.503    0.060    8.335    0.000    0.385    0.621
##    .PU3               0.316    0.070    4.531    0.000    0.179    0.453
##     BI_MM             0.853    0.132    6.447    0.000    0.594    1.112
##     AT_MM             0.449    0.090    4.997    0.000    0.273    0.625
##     SN_MM             1.254    0.109   11.535    0.000    1.041    1.467
##     PBC_MM            1.025    0.130    7.901    0.000    0.771    1.280
##     PRF_MM            3.567    0.215   16.617    0.000    3.146    3.988
##     PU_MM             1.542    0.170    9.065    0.000    1.209    1.876
##    Std.lv  Std.all
##     0.300    0.260
##     0.743    0.381
##     0.842    0.592
##     0.466    0.634
##     0.271    0.376
##     0.336    0.474
##     0.930    0.736
##     0.360    0.402
##     0.798    0.593
##     0.303    0.344
##     0.436    0.375
##     0.216    0.248
##     0.427    0.411
##     0.276    0.181
##     0.388    0.268
##     1.008    0.656
##     0.813    0.563
##     0.289    0.220
##     1.430    0.661
##     0.636    0.413
##     0.885    0.199
##     0.649    0.159
##     0.825    0.244
##     0.952    0.238
##     1.331    0.386
##     2.477    0.603
##     1.084    0.284
##     0.744    0.229
##     1.560    0.355
##     0.791    0.221
##     0.225    0.127
##     0.503    0.293
##     0.316    0.198
##     1.000    1.000
##     1.000    1.000
##     1.000    1.000
##     1.000    1.000
##     1.000    1.000
##     1.000    1.000
## 
## R-Square:
##                    Estimate
##     BI1               0.740
##     BI2               0.619
##     BI3               0.408
##     BI4               0.366
##     AT1               0.624
##     AT2               0.526
##     AT3               0.264
##     AT4               0.598
##     AT5               0.407
##     AT6               0.656
##     AT7               0.625
##     AT8               0.752
##     AT9               0.589
##     SN1               0.819
##     SN2               0.732
##     SN3               0.344
##     SN4               0.437
##     PBC1              0.780
##     PBC2              0.339
##     PBC3              0.587
##     PRF1              0.801
##     PRF2              0.841
##     PRF3              0.756
##     PRF4              0.762
##     PRF5              0.614
##     PRF6              0.397
##     PRF7              0.716
##     PRF8              0.771
##     PRF9              0.645
##     PRF10             0.779
##     PU1               0.873
##     PU2               0.707
##     PU3               0.802
```

## 6.5 Cronbach´s alpha (\(a\)) and average variance extracted (AVE)

Calculate the Cronbach´s \(a\). It
should exceed the threshold of \(a\)
> .7. The AVE should not fall below the recommended threshold of AVE
> .5 (Fornell & Larcker, 1981; Kline, 2015).

```
##            BI_MM     AT_MM     SN_MM    PBC_MM    PRF_MM     PU_MM
## alpha  0.8051802 0.9127886 0.8337482 0.7606097 0.9588949 0.9192496
## omega  0.8227793 0.9128164 0.8443414 0.7715891 0.9597256 0.9204859
## omega2 0.8227793 0.9128164 0.8443414 0.7715891 0.9597256 0.9204859
## omega3 0.8215686 0.9061427 0.8474660 0.7813069 0.9577004 0.9204941
## avevar 0.5532610 0.5411900 0.5830279 0.5307874 0.7066351 0.7946245
```

## 6.6 Composite reliability (CR)

Define the function for CR and calculate it (Google Groups,
2017).

Calculate the CR. It should exceed the recommended threshold of CR
> .7 (Fornell & Larcker, 1981; Kline, 2015).

```
##     BI_MM     AT_MM     SN_MM    PBC_MM    PRF_MM     PU_MM 
## 0.8227793 0.9128164 0.8443414 0.7715891 0.9597256 0.9204859
```

## 6.7 Fornell-Larcker criterion (square root of the AVE and correlations)

Define the function for the square root of the AVE and calculate
it.  
Discriminant validity exists when the square root of the AVE of a latent
construct (diagonal) exceeds its correlation with the other latent
constructs within the model, as this indicates that the modeled
constructs can be reliably separated (Fornell & Larcker, 1981).

Order of square root of the AVE  
1. BI  
2. AT  
3. SN  
4. PBC  
5. PRF  
6. PU

```
## [1] 0.7438152
## [1] 0.7356562
## [1] 0.7635626
## [1] 0.7285516
## [1] 0.8406159
## [1] 0.8914171
```

```
##             BI    AT    SN  PBC   PRF    PU  CINS sport_mv
## BI        1.00  0.67  0.60 0.44 -0.17  0.31  0.37     0.13
## AT        0.67  1.00  0.58 0.32 -0.28  0.36  0.25     0.10
## SN        0.60  0.58  1.00 0.36 -0.24  0.26  0.32     0.09
## PBC       0.44  0.32  0.36 1.00  0.00  0.20  0.20     0.10
## PRF      -0.17 -0.28 -0.24 0.00  1.00 -0.10 -0.17     0.02
## PU        0.31  0.36  0.26 0.20 -0.10  1.00  0.11     0.14
## CINS      0.37  0.25  0.32 0.20 -0.17  0.11  1.00     0.10
## sport_mv  0.13  0.10  0.09 0.10  0.02  0.14  0.10     1.00
## 
## n= 309 
## 
## 
## P
##          BI     AT     SN     PBC    PRF    PU     CINS   sport_mv
## BI              0.0000 0.0000 0.0000 0.0034 0.0000 0.0000 0.0192  
## AT       0.0000        0.0000 0.0000 0.0000 0.0000 0.0000 0.0714  
## SN       0.0000 0.0000        0.0000 0.0000 0.0000 0.0000 0.1169  
## PBC      0.0000 0.0000 0.0000        0.9845 0.0004 0.0006 0.0749  
## PRF      0.0034 0.0000 0.0000 0.9845        0.0934 0.0024 0.6916  
## PU       0.0000 0.0000 0.0000 0.0004 0.0934        0.0605 0.0122  
## CINS     0.0000 0.0000 0.0000 0.0006 0.0024 0.0605        0.0947  
## sport_mv 0.0192 0.0714 0.1169 0.0749 0.6916 0.0122 0.0947
```

## 6.8 Heterotrait-monotrait (HTMT)

Calculate the HTMT. The values of the HTMT should not exceed the
threshold of HTMT < .85 (Henseler et al., 2015).

```
##        BI_MM AT_MM SN_MM PBC_MM PRF_MM PU_MM
## BI_MM  1.000                                
## AT_MM  0.776 1.000                          
## SN_MM  0.738 0.662 1.000                    
## PBC_MM 0.544 0.361 0.437 1.000              
## PRF_MM 0.158 0.284 0.254 0.065  1.000       
## PU_MM  0.333 0.391 0.279 0.237  0.072  1.000
```

---

# 7 Structural equation model (SEM)

## 7.1 Defining the SEM

Define the SEM.

```
## lavaan 0.6-11 ended normally after 56 iterations
## 
##   Estimator                                         ML
##   Optimization method                           NLMINB
##   Number of model parameters                        81
##                                                       
##   Number of observations                           309
##                                                       
## Model Test User Model:
##                                               Standard      Robust
##   Test Statistic                              1132.777     980.724
##   Degrees of freedom                               514         514
##   P-value (Chi-square)                           0.000       0.000
##   Scaling correction factor                                  1.155
##        Satorra-Bentler correction                                 
## 
## Model Test Baseline Model:
## 
##   Test statistic                              8161.167    6359.664
##   Degrees of freedom                               561         561
##   P-value                                        0.000       0.000
##   Scaling correction factor                                  1.283
## 
## User Model versus Baseline Model:
## 
##   Comparative Fit Index (CFI)                    0.919       0.920
##   Tucker-Lewis Index (TLI)                       0.911       0.912
##                                                                   
##   Robust Comparative Fit Index (CFI)                         0.928
##   Robust Tucker-Lewis Index (TLI)                            0.921
## 
## Loglikelihood and Information Criteria:
## 
##   Loglikelihood user model (H0)             -14344.515  -14344.515
##   Loglikelihood unrestricted model (H1)             NA          NA
##                                                                   
##   Akaike (AIC)                               28851.030   28851.030
##   Bayesian (BIC)                             29153.430   29153.430
##   Sample-size adjusted Bayesian (BIC)        28896.530   28896.530
## 
## Root Mean Square Error of Approximation:
## 
##   RMSEA                                          0.062       0.054
##   90 Percent confidence interval - lower         0.058       0.049
##   90 Percent confidence interval - upper         0.067       0.059
##   P-value RMSEA <= 0.05                          0.000       0.074
##                                                                   
##   Robust RMSEA                                               0.058
##   90 Percent confidence interval - lower                     0.053
##   90 Percent confidence interval - upper                     0.064
## 
## Standardized Root Mean Square Residual:
## 
##   SRMR                                           0.077       0.077
## 
## Parameter Estimates:
## 
##   Standard errors                           Robust.sem
##   Information                                 Expected
##   Information saturated (h1) model          Structured
## 
## Latent Variables:
##                    Estimate  Std.Err  z-value  P(>|z|) ci.lower ci.upper
##   BI_MM =~                                                              
##     BI1               1.000                               1.000    1.000
##     BI2               1.191    0.081   14.731    0.000    1.033    1.350
##     BI3               0.845    0.110    7.665    0.000    0.629    1.061
##     BI4               0.578    0.091    6.344    0.000    0.400    0.757
##   AT_MM =~                                                              
##     AT1               1.000                               1.000    1.000
##     AT2               0.910    0.063   14.367    0.000    0.786    1.034
##     AT3               0.862    0.121    7.139    0.000    0.625    1.098
##     AT4               1.091    0.081   13.438    0.000    0.931    1.250
##     AT5               1.104    0.103   10.668    0.000    0.901    1.307
##     AT6               1.134    0.102   11.165    0.000    0.935    1.332
##     AT7               1.272    0.110   11.563    0.000    1.056    1.487
##     AT8               1.207    0.087   13.871    0.000    1.036    1.377
##     AT9               1.168    0.089   13.168    0.000    0.994    1.342
##   SN_MM =~                                                              
##     SN1               1.000                               1.000    1.000
##     SN2               0.915    0.054   16.999    0.000    0.809    1.020
##     SN3               0.651    0.068    9.630    0.000    0.519    0.784
##     SN4               0.708    0.052   13.728    0.000    0.607    0.809
##   PBC_MM =~                                                             
##     PBC1              1.000                               1.000    1.000
##     PBC2              0.782    0.114    6.868    0.000    0.559    1.006
##     PBC3              0.866    0.094    9.174    0.000    0.681    1.051
##   PRF_MM =~                                                             
##     PRF1              1.000                               1.000    1.000
##     PRF2              0.980    0.028   34.884    0.000    0.925    1.036
##     PRF3              0.846    0.033   25.660    0.000    0.782    0.911
##     PRF4              0.925    0.030   31.110    0.000    0.866    0.983
##     PRF5              0.771    0.041   19.011    0.000    0.692    0.850
##     PRF6              0.677    0.046   14.769    0.000    0.587    0.767
##     PRF7              0.876    0.035   24.879    0.000    0.807    0.946
##     PRF8              0.838    0.040   21.140    0.000    0.760    0.915
##     PRF9              0.891    0.035   25.325    0.000    0.822    0.960
##     PRF10             0.884    0.038   23.382    0.000    0.810    0.958
##   PU_MM =~                                                              
##     PU1               1.000                               1.000    1.000
##     PU2               0.888    0.044   20.377    0.000    0.802    0.973
##     PU3               0.912    0.041   22.402    0.000    0.832    0.991
##   CINS_MM =~                                                            
##     CINS              1.000                               1.000    1.000
##    Std.lv  Std.all
##                   
##     0.886    0.845
##     1.055    0.770
##     0.749    0.636
##     0.512    0.605
##                   
##     0.670    0.790
##     0.610    0.725
##     0.578    0.514
##     0.731    0.773
##     0.740    0.638
##     0.760    0.810
##     0.852    0.790
##     0.809    0.867
##     0.783    0.769
##                   
##     1.122    0.907
##     1.026    0.852
##     0.731    0.589
##     0.794    0.661
##                   
##     1.059    0.923
##     0.828    0.563
##     0.917    0.739
##                   
##     1.889    0.895
##     1.852    0.917
##     1.598    0.870
##     1.746    0.873
##     1.456    0.784
##     1.278    0.631
##     1.655    0.847
##     1.582    0.878
##     1.682    0.803
##     1.669    0.882
##                   
##     1.242    0.934
##     1.103    0.841
##     1.132    0.896
##                   
##     1.281    1.000
## 
## Regressions:
##                    Estimate  Std.Err  z-value  P(>|z|) ci.lower ci.upper
##   BI_MM ~                                                               
##     AT_MM     (a1)    0.757    0.099    7.610    0.000    0.562    0.951
##     SN_MM     (a2)    0.179    0.057    3.109    0.002    0.066    0.291
##     PBC_MM    (a3)    0.157    0.041    3.804    0.000    0.076    0.238
##     CINS_MM   (b5)    0.091    0.033    2.740    0.006    0.026    0.156
##   AT_MM ~                                                               
##     SN_MM     (b1)    0.339    0.045    7.524    0.000    0.250    0.427
##     PRF_MM    (b2)   -0.042    0.019   -2.175    0.030   -0.080   -0.004
##     PU_MM     (b3)    0.106    0.031    3.469    0.001    0.046    0.166
##   PBC_MM ~                                                              
##     CINS_MM   (b4)    0.153    0.049    3.125    0.002    0.057    0.248
##    Std.lv  Std.all
##                   
##     0.573    0.573
##     0.226    0.226
##     0.188    0.188
##     0.132    0.132
##                   
##     0.567    0.567
##    -0.118   -0.118
##     0.196    0.196
##                   
##     0.185    0.185
## 
## Covariances:
##                    Estimate  Std.Err  z-value  P(>|z|) ci.lower ci.upper
##   SN_MM ~~                                                              
##     PRF_MM           -0.589    0.142   -4.150    0.000   -0.868   -0.311
##     PU_MM             0.404    0.097    4.166    0.000    0.214    0.594
##     CINS_MM           0.455    0.089    5.094    0.000    0.280    0.630
##   PRF_MM ~~                                                             
##     PU_MM            -0.218    0.160   -1.359    0.174   -0.532    0.096
##     CINS_MM          -0.390    0.135   -2.886    0.004   -0.655   -0.125
##   PU_MM ~~                                                              
##     CINS_MM           0.174    0.091    1.915    0.056   -0.004    0.352
##    Std.lv  Std.all
##                   
##    -0.278   -0.278
##     0.290    0.290
##     0.316    0.316
##                   
##    -0.093   -0.093
##    -0.161   -0.161
##                   
##     0.109    0.109
## 
## Variances:
##                    Estimate  Std.Err  z-value  P(>|z|) ci.lower ci.upper
##    .BI1               0.315    0.051    6.224    0.000    0.216    0.414
##    .BI2               0.763    0.098    7.808    0.000    0.572    0.955
##    .BI3               0.825    0.126    6.553    0.000    0.578    1.071
##    .BI4               0.455    0.065    7.053    0.000    0.329    0.582
##    .AT1               0.271    0.035    7.822    0.000    0.203    0.338
##    .AT2               0.336    0.047    7.081    0.000    0.243    0.429
##    .AT3               0.930    0.091   10.213    0.000    0.752    1.109
##    .AT4               0.361    0.052    6.955    0.000    0.259    0.462
##    .AT5               0.798    0.067   11.925    0.000    0.667    0.929
##    .AT6               0.303    0.041    7.304    0.000    0.222    0.384
##    .AT7               0.436    0.066    6.644    0.000    0.308    0.565
##    .AT8               0.217    0.039    5.538    0.000    0.140    0.293
##    .AT9               0.425    0.052    8.122    0.000    0.322    0.528
##    .SN1               0.272    0.053    5.176    0.000    0.169    0.375
##    .SN2               0.396    0.088    4.520    0.000    0.224    0.567
##    .SN3               1.003    0.172    5.848    0.000    0.667    1.340
##    .SN4               0.814    0.106    7.685    0.000    0.606    1.021
##    .PBC1              0.194    0.092    2.105    0.035    0.013    0.374
##    .PBC2              1.478    0.167    8.846    0.000    1.151    1.806
##    .PBC3              0.701    0.130    5.404    0.000    0.446    0.955
##    .PRF1              0.884    0.125    7.098    0.000    0.640    1.129
##    .PRF2              0.650    0.122    5.334    0.000    0.411    0.888
##    .PRF3              0.824    0.102    8.056    0.000    0.624    1.025
##    .PRF4              0.952    0.120    7.905    0.000    0.716    1.188
##    .PRF5              1.332    0.172    7.754    0.000    0.995    1.668
##    .PRF6              2.475    0.202   12.230    0.000    2.079    2.872
##    .PRF7              1.082    0.124    8.721    0.000    0.839    1.325
##    .PRF8              0.745    0.087    8.595    0.000    0.575    0.915
##    .PRF9              1.560    0.179    8.730    0.000    1.210    1.911
##    .PRF10             0.791    0.113    7.021    0.000    0.570    1.012
##    .PU1               0.225    0.061    3.714    0.000    0.106    0.344
##    .PU2               0.502    0.060    8.316    0.000    0.384    0.620
##    .PU3               0.316    0.070    4.500    0.000    0.179    0.454
##    .CINS              0.000                               0.000    0.000
##    .BI_MM             0.254    0.053    4.781    0.000    0.150    0.358
##    .AT_MM             0.234    0.041    5.637    0.000    0.153    0.315
##     SN_MM             1.258    0.111   11.351    0.000    1.041    1.476
##    .PBC_MM            1.083    0.137    7.902    0.000    0.814    1.351
##     PRF_MM            3.567    0.215   16.588    0.000    3.146    3.989
##     PU_MM             1.542    0.171    9.040    0.000    1.208    1.877
##     CINS_MM           1.642    0.098   16.707    0.000    1.449    1.835
##    Std.lv  Std.all
##     0.315    0.286
##     0.763    0.407
##     0.825    0.595
##     0.455    0.635
##     0.271    0.376
##     0.336    0.475
##     0.930    0.736
##     0.361    0.403
##     0.798    0.593
##     0.303    0.344
##     0.436    0.375
##     0.217    0.249
##     0.425    0.409
##     0.272    0.178
##     0.396    0.273
##     1.003    0.653
##     0.814    0.563
##     0.194    0.147
##     1.478    0.683
##     0.701    0.455
##     0.884    0.199
##     0.650    0.159
##     0.824    0.244
##     0.952    0.238
##     1.332    0.386
##     2.475    0.602
##     1.082    0.283
##     0.745    0.229
##     1.560    0.355
##     0.791    0.221
##     0.225    0.127
##     0.502    0.292
##     0.316    0.198
##     0.000    0.000
##     0.324    0.324
##     0.521    0.521
##     1.000    1.000
##     0.966    0.966
##     1.000    1.000
##     1.000    1.000
##     1.000    1.000
## 
## R-Square:
##                    Estimate
##     BI1               0.714
##     BI2               0.593
##     BI3               0.405
##     BI4               0.365
##     AT1               0.624
##     AT2               0.525
##     AT3               0.264
##     AT4               0.597
##     AT5               0.407
##     AT6               0.656
##     AT7               0.625
##     AT8               0.751
##     AT9               0.591
##     SN1               0.822
##     SN2               0.727
##     SN3               0.347
##     SN4               0.437
##     PBC1              0.853
##     PBC2              0.317
##     PBC3              0.545
##     PRF1              0.801
##     PRF2              0.841
##     PRF3              0.756
##     PRF4              0.762
##     PRF5              0.614
##     PRF6              0.398
##     PRF7              0.717
##     PRF8              0.771
##     PRF9              0.645
##     PRF10             0.779
##     PU1               0.873
##     PU2               0.708
##     PU3               0.802
##     CINS              1.000
##     BI_MM             0.676
##     AT_MM             0.479
##     PBC_MM            0.034
## 
## Defined Parameters:
##                    Estimate  Std.Err  z-value  P(>|z|) ci.lower ci.upper
##     a1b1              0.256    0.043    5.999    0.000    0.173    0.340
##     a1b2             -0.032    0.015   -2.109    0.035   -0.061   -0.002
##     a1b3              0.080    0.026    3.076    0.002    0.029    0.131
##     a3b4              0.024    0.010    2.428    0.015    0.005    0.043
##     total1            0.435    0.057    7.610    0.000    0.323    0.547
##     total2            0.115    0.032    3.549    0.000    0.051    0.178
##    Std.lv  Std.all
##     0.324    0.324
##    -0.067   -0.067
##     0.112    0.112
##     0.035    0.035
##     0.551    0.551
##     0.166    0.166
```

## 7.2 Modifying indicies (facultative)

(Deleting items and) including paths (e.g., covariances) can improve
the overall fit indices.

Important: Do not changed correlation paths between items of
different constructs to avoid over-fitting and violations of content
validity (Gefen et al., 2011)

One option is th check whether you can improve your model using the
`modindices` function. If you decide to add paths to your
model, change one relation at a time and run the model and the analysis
of modification before adding the next.

```
modindices(fitSEM0, sort = TRUE, maximum.number = 20)
```

Define your final SEM.

Run the analysis of the SEM. View the summary of model fit and save
it in a new variable. Analyse the following indixes:  
- the central \(X\)2
distribution (for more details see Gefen, 2011; Jöreskog, 1969)  
- root mean square error of approximation (RMSEA; for more details see
Browne & Cudeck, 1992; Hu & Bentler, 1999; Kamel &
Guillaume, 2019)  
- standardized root mean square residual (SRMR; for more details see
Browne & Cudeck, 1992; Hu & Bentler, 1999; Kamel &
Guillaume, 2019)  
- comparative fit index (CFI; for more details see Kamel &
Guillaume, 2019; Marsh et al., 2004)  
- Tucker-Lewis index (TLI; for more details see Kamel & Guillaume,
2019; Marsh et al., 2004)

```
## lavaan 0.6-11 ended normally after 59 iterations
## 
##   Estimator                                         ML
##   Optimization method                           NLMINB
##   Number of model parameters                        87
##                                                       
##   Number of observations                           309
##                                                       
## Model Test User Model:
##                                               Standard      Robust
##   Test Statistic                               942.948     820.630
##   Degrees of freedom                               508         508
##   P-value (Chi-square)                           0.000       0.000
##   Scaling correction factor                                  1.149
##        Satorra-Bentler correction                                 
## 
## Model Test Baseline Model:
## 
##   Test statistic                              8161.167    6359.664
##   Degrees of freedom                               561         561
##   P-value                                        0.000       0.000
##   Scaling correction factor                                  1.283
## 
## User Model versus Baseline Model:
## 
##   Comparative Fit Index (CFI)                    0.943       0.946
##   Tucker-Lewis Index (TLI)                       0.937       0.940
##                                                                   
##   Robust Comparative Fit Index (CFI)                         0.952
##   Robust Tucker-Lewis Index (TLI)                            0.947
## 
## Loglikelihood and Information Criteria:
## 
##   Loglikelihood user model (H0)             -14249.600  -14249.600
##   Loglikelihood unrestricted model (H1)             NA          NA
##                                                                   
##   Akaike (AIC)                               28673.201   28673.201
##   Bayesian (BIC)                             28998.001   28998.001
##   Sample-size adjusted Bayesian (BIC)        28722.072   28722.072
## 
## Root Mean Square Error of Approximation:
## 
##   RMSEA                                          0.053       0.045
##   90 Percent confidence interval - lower         0.047       0.039
##   90 Percent confidence interval - upper         0.058       0.050
##   P-value RMSEA <= 0.05                          0.200       0.957
##                                                                   
##   Robust RMSEA                                               0.048
##   90 Percent confidence interval - lower                     0.042
##   90 Percent confidence interval - upper                     0.054
## 
## Standardized Root Mean Square Residual:
## 
##   SRMR                                           0.077       0.077
## 
## Parameter Estimates:
## 
##   Standard errors                           Robust.sem
##   Information                                 Expected
##   Information saturated (h1) model          Structured
## 
## Latent Variables:
##                    Estimate  Std.Err  z-value  P(>|z|) ci.lower ci.upper
##   BI_MM =~                                                              
##     BI1               1.000                               1.000    1.000
##     BI2               1.187    0.081   14.624    0.000    1.028    1.346
##     BI3               0.777    0.111    7.021    0.000    0.560    0.993
##     BI4               0.519    0.088    5.923    0.000    0.347    0.691
##   AT_MM =~                                                              
##     AT1               1.000                               1.000    1.000
##     AT2               0.926    0.069   13.505    0.000    0.791    1.060
##     AT3               0.853    0.126    6.772    0.000    0.606    1.100
##     AT4               1.081    0.083   13.002    0.000    0.918    1.244
##     AT5               1.107    0.108   10.269    0.000    0.896    1.318
##     AT6               1.164    0.109   10.722    0.000    0.951    1.377
##     AT7               1.315    0.121   10.904    0.000    1.079    1.552
##     AT8               1.245    0.096   12.922    0.000    1.056    1.434
##     AT9               1.195    0.095   12.544    0.000    1.008    1.381
##   SN_MM =~                                                              
##     SN1               1.000                               1.000    1.000
##     SN2               0.913    0.054   17.040    0.000    0.808    1.018
##     SN3               0.651    0.068    9.620    0.000    0.518    0.783
##     SN4               0.708    0.052   13.735    0.000    0.607    0.809
##   PBC_MM =~                                                             
##     PBC1              1.000                               1.000    1.000
##     PBC2              0.779    0.114    6.847    0.000    0.556    1.002
##     PBC3              0.862    0.096    9.002    0.000    0.674    1.049
##   PRF_MM =~                                                             
##     PRF1              1.000                               1.000    1.000
##     PRF2              0.975    0.028   34.775    0.000    0.920    1.030
##     PRF3              0.842    0.033   25.657    0.000    0.778    0.906
##     PRF4              0.922    0.030   31.224    0.000    0.864    0.980
##     PRF5              0.761    0.040   18.868    0.000    0.682    0.840
##     PRF6              0.664    0.046   14.308    0.000    0.573    0.755
##     PRF7              0.868    0.035   25.137    0.000    0.800    0.936
##     PRF8              0.816    0.039   20.838    0.000    0.739    0.893
##     PRF9              0.875    0.035   24.739    0.000    0.806    0.945
##     PRF10             0.862    0.037   23.099    0.000    0.789    0.935
##   PU_MM =~                                                              
##     PU1               1.000                               1.000    1.000
##     PU2               0.888    0.044   20.379    0.000    0.802    0.973
##     PU3               0.912    0.041   22.406    0.000    0.832    0.991
##   CINS_MM =~                                                            
##     CINS              1.000                               1.000    1.000
##    Std.lv  Std.all
##                   
##     0.911    0.868
##     1.081    0.789
##     0.707    0.599
##     0.473    0.557
##                   
##     0.655    0.773
##     0.607    0.721
##     0.559    0.497
##     0.709    0.749
##     0.726    0.625
##     0.763    0.813
##     0.862    0.799
##     0.816    0.875
##     0.783    0.768
##                   
##     1.122    0.907
##     1.025    0.852
##     0.730    0.589
##     0.795    0.661
##                   
##     1.062    0.926
##     0.827    0.562
##     0.915    0.737
##                   
##     1.905    0.903
##     1.858    0.920
##     1.604    0.873
##     1.757    0.878
##     1.450    0.780
##     1.265    0.624
##     1.654    0.846
##     1.554    0.862
##     1.667    0.796
##     1.642    0.868
##                   
##     1.242    0.934
##     1.102    0.841
##     1.132    0.896
##                   
##     1.281    1.000
## 
## Regressions:
##                    Estimate  Std.Err  z-value  P(>|z|) ci.lower ci.upper
##   BI_MM ~                                                               
##     AT_MM     (a1)    0.795    0.106    7.481    0.000    0.587    1.003
##     SN_MM     (a2)    0.183    0.060    3.062    0.002    0.066    0.300
##     PBC_MM    (a3)    0.151    0.043    3.536    0.000    0.067    0.235
##     CINS_MM   (b5)    0.077    0.035    2.193    0.028    0.008    0.146
##   AT_MM ~                                                               
##     SN_MM     (b1)    0.335    0.045    7.423    0.000    0.247    0.424
##     PRF_MM    (b2)   -0.041    0.019   -2.206    0.027   -0.078   -0.005
##     PU_MM     (b3)    0.099    0.030    3.289    0.001    0.040    0.158
##   PBC_MM ~                                                              
##     CINS_MM   (b4)    0.153    0.049    3.124    0.002    0.057    0.248
##    Std.lv  Std.all
##                   
##     0.572    0.572
##     0.225    0.225
##     0.176    0.176
##     0.108    0.108
##                   
##     0.574    0.574
##    -0.119   -0.119
##     0.187    0.187
##                   
##     0.184    0.184
## 
## Covariances:
##                    Estimate  Std.Err  z-value  P(>|z|) ci.lower ci.upper
##  .AT3 ~~                                                                
##    .AT5               0.342    0.060    5.712    0.000    0.224    0.459
##  .PRF8 ~~                                                               
##    .PRF10             0.334    0.075    4.460    0.000    0.187    0.481
##  .BI3 ~~                                                                
##    .BI4               0.241    0.063    3.830    0.000    0.118    0.364
##  .AT3 ~~                                                                
##    .AT4               0.012    0.039    0.298    0.766   -0.065    0.089
##  .PRF6 ~~                                                               
##    .PRF9              0.647    0.147    4.398    0.000    0.359    0.936
##  .AT1 ~~                                                                
##    .AT4               0.114    0.029    3.922    0.000    0.057    0.171
##   SN_MM ~~                                                              
##     PRF_MM           -0.590    0.143   -4.129    0.000   -0.870   -0.310
##     PU_MM             0.405    0.097    4.175    0.000    0.215    0.595
##     CINS_MM           0.455    0.089    5.099    0.000    0.280    0.630
##   PRF_MM ~~                                                             
##     PU_MM            -0.220    0.161   -1.371    0.170   -0.535    0.095
##     CINS_MM          -0.396    0.137   -2.896    0.004   -0.664   -0.128
##   PU_MM ~~                                                              
##     CINS_MM           0.174    0.091    1.914    0.056   -0.004    0.352
##    Std.lv  Std.all
##                   
##     0.342    0.387
##                   
##     0.334    0.391
##                   
##     0.241    0.361
##                   
##     0.012    0.019
##                   
##     0.647    0.322
##                   
##     0.114    0.338
##                   
##    -0.276   -0.276
##     0.291    0.291
##     0.317    0.317
##                   
##    -0.093   -0.093
##    -0.162   -0.162
##                   
##     0.109    0.109
## 
## Variances:
##                    Estimate  Std.Err  z-value  P(>|z|) ci.lower ci.upper
##    .BI1               0.272    0.047    5.751    0.000    0.179    0.365
##    .BI2               0.711    0.096    7.399    0.000    0.523    0.899
##    .BI3               0.892    0.131    6.801    0.000    0.635    1.149
##    .BI4               0.498    0.070    7.109    0.000    0.361    0.635
##    .AT1               0.290    0.036    8.036    0.000    0.219    0.361
##    .AT2               0.340    0.048    7.072    0.000    0.246    0.434
##    .AT3               0.951    0.090   10.587    0.000    0.775    1.127
##    .AT4               0.393    0.054    7.239    0.000    0.287    0.500
##    .AT5               0.819    0.068   11.983    0.000    0.685    0.953
##    .AT6               0.298    0.042    7.148    0.000    0.216    0.380
##    .AT7               0.420    0.065    6.435    0.000    0.292    0.548
##    .AT8               0.205    0.037    5.470    0.000    0.131    0.278
##    .AT9               0.425    0.053    8.029    0.000    0.321    0.529
##    .SN1               0.271    0.052    5.186    0.000    0.168    0.373
##    .SN2               0.398    0.088    4.542    0.000    0.226    0.569
##    .SN3               1.004    0.171    5.858    0.000    0.668    1.340
##    .SN4               0.813    0.106    7.676    0.000    0.605    1.020
##    .PBC1              0.188    0.094    2.007    0.045    0.004    0.372
##    .PBC2              1.481    0.167    8.855    0.000    1.153    1.809
##    .PBC3              0.704    0.131    5.366    0.000    0.447    0.962
##    .PRF1              0.823    0.123    6.670    0.000    0.581    1.065
##    .PRF2              0.627    0.122    5.134    0.000    0.388    0.867
##    .PRF3              0.806    0.103    7.831    0.000    0.604    1.008
##    .PRF4              0.916    0.120    7.617    0.000    0.681    1.152
##    .PRF5              1.351    0.173    7.803    0.000    1.011    1.690
##    .PRF6              2.509    0.208   12.078    0.000    2.102    2.917
##    .PRF7              1.088    0.124    8.757    0.000    0.844    1.331
##    .PRF8              0.832    0.091    9.120    0.000    0.653    1.011
##    .PRF9              1.612    0.182    8.861    0.000    1.255    1.968
##    .PRF10             0.881    0.113    7.773    0.000    0.659    1.103
##    .PU1               0.225    0.061    3.711    0.000    0.106    0.344
##    .PU2               0.502    0.060    8.313    0.000    0.384    0.621
##    .PU3               0.316    0.070    4.496    0.000    0.178    0.454
##    .CINS              0.000                               0.000    0.000
##    .BI_MM             0.287    0.056    5.147    0.000    0.178    0.396
##    .AT_MM             0.222    0.042    5.330    0.000    0.140    0.303
##     SN_MM             1.260    0.111   11.367    0.000    1.043    1.477
##    .PBC_MM            1.089    0.139    7.817    0.000    0.816    1.361
##     PRF_MM            3.628    0.215   16.898    0.000    3.208    4.049
##     PU_MM             1.542    0.171    9.041    0.000    1.208    1.877
##     CINS_MM           1.642    0.098   16.708    0.000    1.449    1.835
##    Std.lv  Std.all
##     0.272    0.247
##     0.711    0.378
##     0.892    0.641
##     0.498    0.690
##     0.290    0.403
##     0.340    0.480
##     0.951    0.753
##     0.393    0.439
##     0.819    0.609
##     0.298    0.339
##     0.420    0.361
##     0.205    0.235
##     0.425    0.409
##     0.271    0.177
##     0.398    0.275
##     1.004    0.653
##     0.813    0.563
##     0.188    0.143
##     1.481    0.684
##     0.704    0.457
##     0.823    0.185
##     0.627    0.154
##     0.806    0.239
##     0.916    0.229
##     1.351    0.391
##     2.509    0.611
##     1.088    0.285
##     0.832    0.256
##     1.612    0.367
##     0.881    0.246
##     0.225    0.127
##     0.502    0.292
##     0.316    0.198
##     0.000    0.000
##     0.346    0.346
##     0.516    0.516
##     1.000    1.000
##     0.966    0.966
##     1.000    1.000
##     1.000    1.000
##     1.000    1.000
## 
## R-Square:
##                    Estimate
##     BI1               0.753
##     BI2               0.622
##     BI3               0.359
##     BI4               0.310
##     AT1               0.597
##     AT2               0.520
##     AT3               0.247
##     AT4               0.561
##     AT5               0.391
##     AT6               0.661
##     AT7               0.639
##     AT8               0.765
##     AT9               0.591
##     SN1               0.823
##     SN2               0.725
##     SN3               0.347
##     SN4               0.437
##     PBC1              0.857
##     PBC2              0.316
##     PBC3              0.543
##     PRF1              0.815
##     PRF2              0.846
##     PRF3              0.761
##     PRF4              0.771
##     PRF5              0.609
##     PRF6              0.389
##     PRF7              0.715
##     PRF8              0.744
##     PRF9              0.633
##     PRF10             0.754
##     PU1               0.873
##     PU2               0.708
##     PU3               0.802
##     CINS              1.000
##     BI_MM             0.654
##     AT_MM             0.484
##     PBC_MM            0.034
## 
## Defined Parameters:
##                    Estimate  Std.Err  z-value  P(>|z|) ci.lower ci.upper
##     a1b1              0.267    0.044    6.012    0.000    0.180    0.354
##     a1b2             -0.033    0.015   -2.130    0.033   -0.063   -0.003
##     a1b3              0.079    0.026    2.966    0.003    0.027    0.131
##     a3b4              0.023    0.010    2.347    0.019    0.004    0.042
##     total1            0.449    0.059    7.626    0.000    0.334    0.565
##     total2            0.100    0.034    2.933    0.003    0.033    0.167
##    Std.lv  Std.all
##     0.329    0.329
##    -0.068   -0.068
##     0.107    0.107
##     0.032    0.032
##     0.554    0.554
##     0.141    0.141
```

## 7.3 Calculating the \(f\)2

Calculate the \(f\)2 for
each scenario (leave one path out at a time; for more details see Cohen,
1988).

---


---

Use the following formula (Chin, 1998; Cohen, 1988):

\(f\)2 = \(R\)2included – \(R\)2excluded / (1 –
\(R\)2included)

\(R\)2included
:

```
  BI  = 0.654  
  AT  = 0.484  
  PBC = 0.034
```

---

The path from AT to BI was omitted in this model. How does the \(f\)2 turn out?

```
f2_BI_AT
## [1] 0.5028902
```

The path from SN to BI was omitted in this model. How does the \(f\)2 turn out?

```
f2_BI_SN
## [1] 0.03468208
```

The path from PBC to BI was omitted in this model. How does the \(f\)2 turn out?

```
f2_BI_PBC
## [1] 0.0867052
```

The path from SN to AT was omitted in this model. How does the \(f\)2 turn out?

```
f2_AT_SN
## [1] 0.5271318
```

The path from PRF to AT was omitted in this model. How does the \(f\)2 turn out?

```
f2_AT_PRF
## [1] 0.01550388
```

The path from PU to AT was omitted in this model. How does the \(f\)2 turn out?

```
f2_AT_PU
## [1] 0.04457364
```

The path from CINS to PBC was omitted in this model. How does the
\(f\)2 turn out? (The path
remains the same, as it is the only one on PBC.)

```
f2_PBC_CIN 
## [1] 0.03519669
```

The path from PU to AT was omitted in this model. How does the \(f\)2 turn out?

```
f2_BI_CINS
## [1] 0.03468208
```

## 7.4 Mediators, indirect and total effects

### 7.4.1 Installing and loading packages

Load the package `bruceR` only right before using it.
Otherwise an other packages such as the `EFAtools` could be
masked and an error message could be displayed (e.g.,“Attaching package:
‘bruceR’ The following object is masked from ‘package:EFAtools’:EFA”).
Also, load the package `lavaan` again.

```
if(!require(pacman, quietly = TRUE)) install.packages("pacman")
p_unload("pacman", negate = TRUE)
p_load("bruceR",                   # bias-corrected and accelerated (BCa) percentile bootstrapping (Bao, 2022)
       "lavaan")                   # boot (Rosseel, 2012)
```

### 7.4.2 Analyse the mediators, indirect and total effects

In lavaan, the standard method for calculating effects is the delta
method (Sobel, 1982). As this method is critically discussed (Zhao et
al., 2010), also conduct a bootstrap with 5,000 resamples (Bao, 2022;
Preacher & Hayes, 2008).  
**NOTE:** RStudio needs some time for bootstrapping and
uses a far amount of your systems working memory capacity.

```
## lavaan 0.6-11 ended normally after 59 iterations
## 
##   Estimator                                         ML
##   Optimization method                           NLMINB
##   Number of model parameters                        87
##                                                       
##   Number of observations                           309
##                                                       
## Model Test User Model:
##                                               Standard      Robust
##   Test Statistic                               942.948     820.630
##   Degrees of freedom                               508         508
##   P-value (Chi-square)                           0.000       0.000
##   Scaling correction factor                                  1.149
##        Satorra-Bentler correction                                 
## 
## Model Test Baseline Model:
## 
##   Test statistic                              8161.167    6359.664
##   Degrees of freedom                               561         561
##   P-value                                        0.000       0.000
##   Scaling correction factor                                  1.283
## 
## User Model versus Baseline Model:
## 
##   Comparative Fit Index (CFI)                    0.943       0.946
##   Tucker-Lewis Index (TLI)                       0.937       0.940
##                                                                   
##   Robust Comparative Fit Index (CFI)                         0.952
##   Robust Tucker-Lewis Index (TLI)                            0.947
## 
## Loglikelihood and Information Criteria:
## 
##   Loglikelihood user model (H0)             -14249.600  -14249.600
##   Loglikelihood unrestricted model (H1)             NA          NA
##                                                                   
##   Akaike (AIC)                               28673.201   28673.201
##   Bayesian (BIC)                             28998.001   28998.001
##   Sample-size adjusted Bayesian (BIC)        28722.072   28722.072
## 
## Root Mean Square Error of Approximation:
## 
##   RMSEA                                          0.053       0.045
##   90 Percent confidence interval - lower         0.047       0.039
##   90 Percent confidence interval - upper         0.058       0.050
##   P-value RMSEA <= 0.05                          0.200       0.957
##                                                                   
##   Robust RMSEA                                               0.048
##   90 Percent confidence interval - lower                     0.042
##   90 Percent confidence interval - upper                     0.054
## 
## Standardized Root Mean Square Residual:
## 
##   SRMR                                           0.077       0.077
## 
## Parameter Estimates:
## 
##   Standard errors                           Robust.sem
##   Information                                 Expected
##   Information saturated (h1) model          Structured
## 
## Latent Variables:
##                    Estimate  Std.Err  z-value  P(>|z|) ci.lower ci.upper
##   BI_MM =~                                                              
##     BI1               1.000                               1.000    1.000
##     BI2               1.187    0.081   14.624    0.000    1.028    1.346
##     BI3               0.777    0.111    7.021    0.000    0.560    0.993
##     BI4               0.519    0.088    5.923    0.000    0.347    0.691
##   AT_MM =~                                                              
##     AT1               1.000                               1.000    1.000
##     AT2               0.926    0.069   13.505    0.000    0.791    1.060
##     AT3               0.853    0.126    6.772    0.000    0.606    1.100
##     AT4               1.081    0.083   13.002    0.000    0.918    1.244
##     AT5               1.107    0.108   10.269    0.000    0.896    1.318
##     AT6               1.164    0.109   10.722    0.000    0.951    1.377
##     AT7               1.315    0.121   10.904    0.000    1.079    1.552
##     AT8               1.245    0.096   12.922    0.000    1.056    1.434
##     AT9               1.195    0.095   12.544    0.000    1.008    1.381
##   SN_MM =~                                                              
##     SN1               1.000                               1.000    1.000
##     SN2               0.913    0.054   17.040    0.000    0.808    1.018
##     SN3               0.651    0.068    9.620    0.000    0.518    0.783
##     SN4               0.708    0.052   13.735    0.000    0.607    0.809
##   PBC_MM =~                                                             
##     PBC1              1.000                               1.000    1.000
##     PBC2              0.779    0.114    6.847    0.000    0.556    1.002
##     PBC3              0.862    0.096    9.002    0.000    0.674    1.049
##   PRF_MM =~                                                             
##     PRF1              1.000                               1.000    1.000
##     PRF2              0.975    0.028   34.775    0.000    0.920    1.030
##     PRF3              0.842    0.033   25.657    0.000    0.778    0.906
##     PRF4              0.922    0.030   31.224    0.000    0.864    0.980
##     PRF5              0.761    0.040   18.868    0.000    0.682    0.840
##     PRF6              0.664    0.046   14.308    0.000    0.573    0.755
##     PRF7              0.868    0.035   25.137    0.000    0.800    0.936
##     PRF8              0.816    0.039   20.838    0.000    0.739    0.893
##     PRF9              0.875    0.035   24.739    0.000    0.806    0.945
##     PRF10             0.862    0.037   23.099    0.000    0.789    0.935
##   PU_MM =~                                                              
##     PU1               1.000                               1.000    1.000
##     PU2               0.888    0.044   20.379    0.000    0.802    0.973
##     PU3               0.912    0.041   22.406    0.000    0.832    0.991
##   CINS_MM =~                                                            
##     CINS              1.000                               1.000    1.000
##    Std.lv  Std.all
##                   
##     0.911    0.868
##     1.081    0.789
##     0.707    0.599
##     0.473    0.557
##                   
##     0.655    0.773
##     0.607    0.721
##     0.559    0.497
##     0.709    0.749
##     0.726    0.625
##     0.763    0.813
##     0.862    0.799
##     0.816    0.875
##     0.783    0.768
##                   
##     1.122    0.907
##     1.025    0.852
##     0.730    0.589
##     0.795    0.661
##                   
##     1.062    0.926
##     0.827    0.562
##     0.915    0.737
##                   
##     1.905    0.903
##     1.858    0.920
##     1.604    0.873
##     1.757    0.878
##     1.450    0.780
##     1.265    0.624
##     1.654    0.846
##     1.554    0.862
##     1.667    0.796
##     1.642    0.868
##                   
##     1.242    0.934
##     1.102    0.841
##     1.132    0.896
##                   
##     1.281    1.000
## 
## Regressions:
##                    Estimate  Std.Err  z-value  P(>|z|) ci.lower ci.upper
##   BI_MM ~                                                               
##     AT_MM     (a1)    0.795    0.106    7.481    0.000    0.587    1.003
##     SN_MM     (a2)    0.183    0.060    3.062    0.002    0.066    0.300
##     PBC_MM    (a3)    0.151    0.043    3.536    0.000    0.067    0.235
##     CINS_MM   (b5)    0.077    0.035    2.193    0.028    0.008    0.146
##   AT_MM ~                                                               
##     SN_MM     (b1)    0.335    0.045    7.423    0.000    0.247    0.424
##     PRF_MM    (b2)   -0.041    0.019   -2.206    0.027   -0.078   -0.005
##     PU_MM     (b3)    0.099    0.030    3.289    0.001    0.040    0.158
##   PBC_MM ~                                                              
##     CINS_MM   (b4)    0.153    0.049    3.124    0.002    0.057    0.248
##    Std.lv  Std.all
##                   
##     0.572    0.572
##     0.225    0.225
##     0.176    0.176
##     0.108    0.108
##                   
##     0.574    0.574
##    -0.119   -0.119
##     0.187    0.187
##                   
##     0.184    0.184
## 
## Covariances:
##                    Estimate  Std.Err  z-value  P(>|z|) ci.lower ci.upper
##  .AT3 ~~                                                                
##    .AT5               0.342    0.060    5.712    0.000    0.224    0.459
##  .PRF8 ~~                                                               
##    .PRF10             0.334    0.075    4.460    0.000    0.187    0.481
##  .BI3 ~~                                                                
##    .BI4               0.241    0.063    3.830    0.000    0.118    0.364
##  .AT3 ~~                                                                
##    .AT4               0.012    0.039    0.298    0.766   -0.065    0.089
##  .PRF6 ~~                                                               
##    .PRF9              0.647    0.147    4.398    0.000    0.359    0.936
##  .AT1 ~~                                                                
##    .AT4               0.114    0.029    3.922    0.000    0.057    0.171
##   SN_MM ~~                                                              
##     PRF_MM           -0.590    0.143   -4.129    0.000   -0.870   -0.310
##     PU_MM             0.405    0.097    4.175    0.000    0.215    0.595
##     CINS_MM           0.455    0.089    5.099    0.000    0.280    0.630
##   PRF_MM ~~                                                             
##     PU_MM            -0.220    0.161   -1.371    0.170   -0.535    0.095
##     CINS_MM          -0.396    0.137   -2.896    0.004   -0.664   -0.128
##   PU_MM ~~                                                              
##     CINS_MM           0.174    0.091    1.914    0.056   -0.004    0.352
##    Std.lv  Std.all
##                   
##     0.342    0.387
##                   
##     0.334    0.391
##                   
##     0.241    0.361
##                   
##     0.012    0.019
##                   
##     0.647    0.322
##                   
##     0.114    0.338
##                   
##    -0.276   -0.276
##     0.291    0.291
##     0.317    0.317
##                   
##    -0.093   -0.093
##    -0.162   -0.162
##                   
##     0.109    0.109
## 
## Variances:
##                    Estimate  Std.Err  z-value  P(>|z|) ci.lower ci.upper
##    .BI1               0.272    0.047    5.751    0.000    0.179    0.365
##    .BI2               0.711    0.096    7.399    0.000    0.523    0.899
##    .BI3               0.892    0.131    6.801    0.000    0.635    1.149
##    .BI4               0.498    0.070    7.109    0.000    0.361    0.635
##    .AT1               0.290    0.036    8.036    0.000    0.219    0.361
##    .AT2               0.340    0.048    7.072    0.000    0.246    0.434
##    .AT3               0.951    0.090   10.587    0.000    0.775    1.127
##    .AT4               0.393    0.054    7.239    0.000    0.287    0.500
##    .AT5               0.819    0.068   11.983    0.000    0.685    0.953
##    .AT6               0.298    0.042    7.148    0.000    0.216    0.380
##    .AT7               0.420    0.065    6.435    0.000    0.292    0.548
##    .AT8               0.205    0.037    5.470    0.000    0.131    0.278
##    .AT9               0.425    0.053    8.029    0.000    0.321    0.529
##    .SN1               0.271    0.052    5.186    0.000    0.168    0.373
##    .SN2               0.398    0.088    4.542    0.000    0.226    0.569
##    .SN3               1.004    0.171    5.858    0.000    0.668    1.340
##    .SN4               0.813    0.106    7.676    0.000    0.605    1.020
##    .PBC1              0.188    0.094    2.007    0.045    0.004    0.372
##    .PBC2              1.481    0.167    8.855    0.000    1.153    1.809
##    .PBC3              0.704    0.131    5.366    0.000    0.447    0.962
##    .PRF1              0.823    0.123    6.670    0.000    0.581    1.065
##    .PRF2              0.627    0.122    5.134    0.000    0.388    0.867
##    .PRF3              0.806    0.103    7.831    0.000    0.604    1.008
##    .PRF4              0.916    0.120    7.617    0.000    0.681    1.152
##    .PRF5              1.351    0.173    7.803    0.000    1.011    1.690
##    .PRF6              2.509    0.208   12.078    0.000    2.102    2.917
##    .PRF7              1.088    0.124    8.757    0.000    0.844    1.331
##    .PRF8              0.832    0.091    9.120    0.000    0.653    1.011
##    .PRF9              1.612    0.182    8.861    0.000    1.255    1.968
##    .PRF10             0.881    0.113    7.773    0.000    0.659    1.103
##    .PU1               0.225    0.061    3.711    0.000    0.106    0.344
##    .PU2               0.502    0.060    8.313    0.000    0.384    0.621
##    .PU3               0.316    0.070    4.496    0.000    0.178    0.454
##    .CINS              0.000                               0.000    0.000
##    .BI_MM             0.287    0.056    5.147    0.000    0.178    0.396
##    .AT_MM             0.222    0.042    5.330    0.000    0.140    0.303
##     SN_MM             1.260    0.111   11.367    0.000    1.043    1.477
##    .PBC_MM            1.089    0.139    7.817    0.000    0.816    1.361
##     PRF_MM            3.628    0.215   16.898    0.000    3.208    4.049
##     PU_MM             1.542    0.171    9.041    0.000    1.208    1.877
##     CINS_MM           1.642    0.098   16.708    0.000    1.449    1.835
##    Std.lv  Std.all
##     0.272    0.247
##     0.711    0.378
##     0.892    0.641
##     0.498    0.690
##     0.290    0.403
##     0.340    0.480
##     0.951    0.753
##     0.393    0.439
##     0.819    0.609
##     0.298    0.339
##     0.420    0.361
##     0.205    0.235
##     0.425    0.409
##     0.271    0.177
##     0.398    0.275
##     1.004    0.653
##     0.813    0.563
##     0.188    0.143
##     1.481    0.684
##     0.704    0.457
##     0.823    0.185
##     0.627    0.154
##     0.806    0.239
##     0.916    0.229
##     1.351    0.391
##     2.509    0.611
##     1.088    0.285
##     0.832    0.256
##     1.612    0.367
##     0.881    0.246
##     0.225    0.127
##     0.502    0.292
##     0.316    0.198
##     0.000    0.000
##     0.346    0.346
##     0.516    0.516
##     1.000    1.000
##     0.966    0.966
##     1.000    1.000
##     1.000    1.000
##     1.000    1.000
## 
## R-Square:
##                    Estimate
##     BI1               0.753
##     BI2               0.622
##     BI3               0.359
##     BI4               0.310
##     AT1               0.597
##     AT2               0.520
##     AT3               0.247
##     AT4               0.561
##     AT5               0.391
##     AT6               0.661
##     AT7               0.639
##     AT8               0.765
##     AT9               0.591
##     SN1               0.823
##     SN2               0.725
##     SN3               0.347
##     SN4               0.437
##     PBC1              0.857
##     PBC2              0.316
##     PBC3              0.543
##     PRF1              0.815
##     PRF2              0.846
##     PRF3              0.761
##     PRF4              0.771
##     PRF5              0.609
##     PRF6              0.389
##     PRF7              0.715
##     PRF8              0.744
##     PRF9              0.633
##     PRF10             0.754
##     PU1               0.873
##     PU2               0.708
##     PU3               0.802
##     CINS              1.000
##     BI_MM             0.654
##     AT_MM             0.484
##     PBC_MM            0.034
## 
## Defined Parameters:
##                    Estimate  Std.Err  z-value  P(>|z|) ci.lower ci.upper
##     a1b1              0.267    0.044    6.012    0.000    0.180    0.354
##     a1b2             -0.033    0.015   -2.130    0.033   -0.063   -0.003
##     a1b3              0.079    0.026    2.966    0.003    0.027    0.131
##     a3b4              0.023    0.010    2.347    0.019    0.004    0.042
##     total1            0.449    0.059    7.626    0.000    0.334    0.565
##     total2            0.100    0.034    2.933    0.003    0.033    0.167
##    Std.lv  Std.all
##     0.329    0.329
##    -0.068   -0.068
##     0.107    0.107
##     0.032    0.032
##     0.554    0.554
##     0.141    0.141
```

```
## 
## Fit Measures (lavaan):
## χ²(508, N = 309) = 942.948, p = 2e-28 ***
## χ²/df = 1.856
## AIC = 28673.201 (Akaike Information Criterion)
## BIC = 28998.001 (Bayesian Information Criterion)
## CFI = 0.943 (Comparative Fit Index)
## TLI = 0.937 (Tucker-Lewis Index; Non-Normed Fit Index, NNFI)
## NFI = 0.884 (Normed Fit Index)
## IFI = 0.943 (Incremental Fit Index)
## GFI = 0.851 (Goodness-of-Fit Index)
## AGFI = 0.826 (Adjusted Goodness-of-Fit Index)
## RMSEA = 0.053, 90% CI [0.047, 0.058] (Root Mean Square Error of Approximation)
## SRMR = 0.077 (Standardized Root Mean Square Residual)
## 
## Model Estimates (lavaan):
## ────────────────────────────────────────────────────────────────────────────────────
##                           Estimate    S.E.      z     p     BootLLCI BootULCI   Beta
## ────────────────────────────────────────────────────────────────────────────────────
## Latent Variables:                                                                   
##   BI_MM =~ BI1               1.000 (0.000)    Inf <.001 ***                    0.868
##   BI_MM =~ BI2               1.187 (0.077) 15.415 <.001 ***    1.063    1.373  0.789
##   BI_MM =~ BI3               0.777 (0.110)  7.050 <.001 ***    0.583    1.010  0.599
##   BI_MM =~ BI4               0.519 (0.092)  5.666 <.001 ***    0.369    0.730  0.557
##   AT_MM =~ AT1               1.000 (0.000)    Inf <.001 ***                    0.773
##   AT_MM =~ AT2               0.926 (0.072) 12.837 <.001 ***    0.813    1.105  0.721
##   AT_MM =~ AT3               0.853 (0.134)  6.372 <.001 ***    0.642    1.189  0.497
##   AT_MM =~ AT4               1.081 (0.088) 12.330 <.001 ***    0.937    1.285  0.749
##   AT_MM =~ AT5               1.107 (0.111)  9.954 <.001 ***    0.928    1.370  0.625
##   AT_MM =~ AT6               1.164 (0.114) 10.181 <.001 ***    0.987    1.441  0.813
##   AT_MM =~ AT7               1.315 (0.125) 10.485 <.001 ***    1.124    1.624  0.799
##   AT_MM =~ AT8               1.245 (0.101) 12.388 <.001 ***    1.099    1.509  0.875
##   AT_MM =~ AT9               1.195 (0.099) 12.014 <.001 ***    1.033    1.437  0.768
##   SN_MM =~ SN1               1.000 (0.000)    Inf <.001 ***                    0.907
##   SN_MM =~ SN2               0.913 (0.049) 18.617 <.001 ***    0.820    1.011  0.852
##   SN_MM =~ SN3               0.651 (0.070)  9.351 <.001 ***    0.516    0.789  0.589
##   SN_MM =~ SN4               0.708 (0.054) 13.020 <.001 ***    0.600    0.812  0.661
##   PBC_MM =~ PBC1             1.000 (0.000)    Inf <.001 ***                    0.926
##   PBC_MM =~ PBC2             0.779 (0.119)  6.537 <.001 ***    0.563    1.025  0.562
##   PBC_MM =~ PBC3             0.862 (0.088)  9.774 <.001 ***    0.682    1.030  0.737
##   PRF_MM =~ PRF1             1.000 (0.000)    Inf <.001 ***                    0.903
##   PRF_MM =~ PRF2             0.975 (0.029) 34.156 <.001 ***    0.916    1.030  0.920
##   PRF_MM =~ PRF3             0.842 (0.034) 25.099 <.001 ***    0.772    0.906  0.873
##   PRF_MM =~ PRF4             0.922 (0.030) 30.988 <.001 ***    0.863    0.982  0.878
##   PRF_MM =~ PRF5             0.761 (0.042) 18.188 <.001 ***    0.675    0.841  0.780
##   PRF_MM =~ PRF6             0.664 (0.046) 14.357 <.001 ***    0.570    0.752  0.624
##   PRF_MM =~ PRF7             0.868 (0.035) 24.513 <.001 ***    0.799    0.937  0.846
##   PRF_MM =~ PRF8             0.816 (0.042) 19.591 <.001 ***    0.732    0.897  0.862
##   PRF_MM =~ PRF9             0.875 (0.036) 23.985 <.001 ***    0.800    0.943  0.796
##   PRF_MM =~ PRF10            0.862 (0.039) 21.843 <.001 ***    0.783    0.936  0.868
##   PU_MM =~ PU1               1.000 (0.000)    Inf <.001 ***                    0.934
##   PU_MM =~ PU2               0.888 (0.044) 20.034 <.001 ***    0.802    0.976  0.841
##   PU_MM =~ PU3               0.912 (0.041) 22.491 <.001 ***    0.828    0.989  0.896
##   CINS_MM =~ CINS            1.000 (0.000)    Inf <.001 ***                    1.000
## Regression Paths:                                                                   
##   BI_MM <- AT_MM (a1)        0.795 (0.117)  6.824 <.001 ***    0.579    1.035  0.572
##   BI_MM <- SN_MM (a2)        0.183 (0.066)  2.759  .006 **     0.056    0.318  0.225
##   BI_MM <- PBC_MM (a3)       0.151 (0.052)  2.876  .004 **     0.050    0.255  0.176
##   BI_MM <- CINS_MM (b5)      0.077 (0.035)  2.199  .028 *      0.012    0.147  0.108
##   AT_MM <- SN_MM (b1)        0.335 (0.047)  7.123 <.001 ***    0.246    0.432  0.574
##   AT_MM <- PRF_MM (b2)      -0.041 (0.019) -2.188  .029 *     -0.080   -0.006 -0.119
##   AT_MM <- PU_MM (b3)        0.099 (0.031)  3.208  .001 **     0.047    0.168  0.187
##   PBC_MM <- CINS_MM (b4)     0.153 (0.050)  3.047  .002 **     0.058    0.253  0.184
## Defined Effects:                                                                    
##   a1b1                       0.267 (0.047)  5.626 <.001 ***    0.186    0.379  0.329
##   a1b2                      -0.033 (0.016) -2.086  .037 *     -0.067   -0.005 -0.068
##   a1b3                       0.079 (0.028)  2.804  .005 **     0.034    0.145  0.107
##   a3b4                       0.023 (0.011)  2.009  .045 *      0.006    0.053  0.032
##   total1                     0.449 (0.067)  6.686 <.001 ***    0.323    0.585  0.554
##   total2                     0.100 (0.035)  2.830  .005 **     0.033    0.172  0.141
## ────────────────────────────────────────────────────────────────────────────────────
## Note. Bias-Corrected and Accelerated (BCa) Percentile Bootstrap Confidence Interval (CI) and SE.
```

To cite all used packages, you can use the function
`citation()`. Include the references in your reference
list.

```
citation("rstudioapi")
citation("here")
citation("lattice")
citation("survival")
citation("Formula")
citation("ggplot2")
citation("Hmisc")
citation("pacman")
citation("dplyr")
citation("naniar")
citation("haven")
citation("psych")                 
citation("car")                   
citation("janitor")               
citation("questionr")  
citation("MVN")
citation("lmtest")
citation("carData")
citation("corrplot")
citation("apaTables")
citation("PerformanceAnalytics")
citation("RColorBrewer")
citation("lavaan")   
citation("GPArotation")
citation("semTools")
citation("EFAtools")
citation("bruceR")
```

# 8 References

Arbuckle, J. L., Marcoulides, G. A., & Schumacker, R. E. (1996).
Full information estimation in the presence of incomplete data. In G.
Marcoulides & R. Schumacker (Eds.), Advanced structural equation
modeling: Issues and techniques (Vol. 243, pp. 277). Lawrence
Erlbaum.  
Bao, H.-W.-S. (2022). bruceR: Broadly useful convenient and efficient R
functions. R package version 0.8.x. https://CRAN.R-project.org/package=bruceR Barnier, J.,
Briatte, F., & Larmarange, J. (2022). questionr: Functions to make
surveys processing easier. R package version 0.7.7. https://CRAN.R-project.org/package=questionr.  
Bernaards, C. A., & Jennrich, R. I. (2005). Gradient projection
algorithms and software for arbitrary rotation criteria in factor
analysis. Educational and Psychological Measurement, 65, 676-696. http://www.stat.ucla.edu/research/gpa  
Browne, M. W., & Cudeck, R. (1992). Alternative ways of assessing
model fit. Sociological Methods & Research, 21(2), 230–258. https://doi.org/https://doi.org/10.1177/0049124192021002005  
Chin, W. W. (1998). The partial least squares approach to structural
equation modeling. In G. A. Marcoulides (Ed.), Modern methods for
business research (Vol. 295, pp. 295–336). Lawrence Erlbaum.  
Cohen, J. (1988). Statistical power for the social sciences.  
Davison, A. C., & Hinkley, D. V. (1997) Bootstrap methods and their
αpplications. Cambridge University Press, Cambridge. ISBN
0-521-57391-2  
Enders, C. K. (2006). Analyzing structural equation models with missing
data. Structural equation modeling: A second course, 2, 493-519.  
Enders, C. K. (2013). Analyzing structural equation models with missing
data. In G. R. Hancock & R. O. Mueller (Eds.), Structural equation
modeling: A second course (pp. 493–519). IAP Information Age
Publishing.  
Firke, S. (2021). janitor: Simple tools for examining and cleaning dirty
data. R package version 2.1.0. https://CRAN.R-project.org/package=janitor  
Fornell, C., & Larcker, D. F. (1981). Evaluating structural equation
models with unobservable variables and measurement error. Journal of
Marketing Research, 18(1), 39–50. https://doi.org/https://doi.org/10.2307/3151312  
Fox, J., & Weisberg, S. (2019). An R companion to applied
regression. Sage Publications. https://socialsciences.mcmaster.ca/jfox/Books/Companion/  
Gefen, D., Rigdon, E. E., & Straub, D. W. (2011). An update and
extension to SEM guidelines for admnistrative and social science
research. Management Information Systems Quarterly, 35. https://doi.org/https://doi.org/10.2307/23044042  
Fox, J., Weisberg, S., & Price, B. (2022). carData: Companion to
applied regression data sets. R package version 3.0-5. https://CRAN.R-project.org/package=carData  
Google Groups (2017). Composite reliability (CFA). https://groups.google.com/g/lavaan/c/TpWINJo\_CRI  
Grund, S. (2017). Multiple imputation of missing data in multilevel
research Christian-Albrechts-University of Kiel]. Kiel, Germany.  
Hair, J. F., Black, W. C., Babin, B. J., & Anderson, R. E. (2010).
Multivariate data: Analysis & global perspectives. Pearson
Education.  
Harrell, F. E. Jr. (2022). Hmisc: Harrell miscellaneous. R package
version 4.7-0. https://CRAN.R-project.org/package=Hmisc  
Henseler, J., Ringle, C. M., & Sarstedt, M. (2015). A new criterion
for assessing discriminant validity in variance-based structural
equation modeling. Journal of the Academy of Marketing Science, 43(1),
115–135. https://doi.org/https://doi.org/10.1007/s11747-014-0403-8  
Hu, L. t., & Bentler, P. M. (1999). Cutoff criteria for fit indexes
in covariance structure analysis: Conventional criteria versus new
alternatives. Structural equation modeling: A multidisciplinary journal,
6(1), 1–55. https://doi.org/https://doi.org/10.1080/10705519909540118  
International Business Machines Corporation (2021). IBM SPSS Statistics
for MacOS (Version 28.0) [Computer software]. Armonk, NY:IBM Corp.

Jöreskog, K. G. (1969). A general approach to confirmatory maximum
likelihood factor analysis. Psychometrika, 34(2), 183–202. https://doi.org/https://doi.org/10.1007/BF02289343  
Jorgensen, T. D., Pornprasertmanit, S., Schoemann, A. M., & Rosseel,
Y. (2022). semTools: Useful tools for structural equation modeling. R
package version 0.5-6. https://CRAN.R-project.org/package=semTools  
Kamel, G., & Guillaume, B. (2019). Structural equation modeling with
lavaan. John Wiley & Sons.  
Kline, R. B. (2015). Principles and practice of structural equation
modeling. Guilford Press. https://search.library.wisc.edu/catalog/9910110667902121  
Korkmaz, S., Göksülük, D., & Zararsiz, G. (2014). MVN: An R package
for assessing multivariate normality. R JOURNAL, 6(2). https://doi.org/https://journal.r-project.org/archive/2014-2/korkmaz-goksuluk-zararsiz.pdf  
Little, R. J. (1988). A test of missing completely at random for
multivariate data with missing values. Journal of the American
Statistical Association, 83(404), 1198–1202. https://doi.org/10.1080/01621459.1988.10478722  
Marsh, H. W., Hau, K.-T., & Wen, Z. (2004). In search of golden
rules: Comment on hypothesis-testing approaches to setting cutoff values
for fit indexes and dangers in overgeneralizing Hu and Bentler’s (1999)
findings. Structural equation modeling: A multidisciplinary journal,
11(3), 320–341. https://doi.org/https://doi.org/10.1207/s15328007sem1103\_2  
Ministry for School and Education of North Rhine-Westphalia. (2021).
Curricula for the primary level in North Rhine-Westphalia - German,
English, Art, Mathematics, Müller K (2020). Here: A simpler way to find
your files. R package version 1.0.1. https://CRAN.R-project.org/package=here  
Music, Practical Philosophy, Protestant Religious Education, Catholic
Religious Education, Social Studies and General Science, Sport
[Lehrpläne für die Primarstufe in Nordrhein-Westfalen - Deutsch,
Englisch, Kunst, Mathematik, Musik, Praktische Philosophie, Evangelische
Religionslehre, Katholische Religionslehre, Sachunterricht, Sport].
Retrieved 06 July 2022 from https://www.schulentwicklung.nrw.de/lehrplaene/upload/klp\_PS/ps\_lp\_sammelband\_2021\_08\_02.pdf  
Neter, J., Wasserman, W., & Kutner, M. H. (1990). Applied linear
statistical models: Regression, analysis of variance, and experimental
designs (3 ed.). Burr Ridge (Ill.) : Irwin. http://lib.ugent.be/catalog/rug01:000244432  
Neuwirth, E. (2022). RColorBrewer: ColorBrewer Palettes. R package
version 1.1-3. https://CRAN.R-project.org/package=RColorBrewer  
Peterson, B. G., Carl, P. (2020). PerformanceAnalytics: Econometric
tools for performance and risk analysis. R package version 2.0.4. https://CRAN.R-project.org/package=PerformanceAnalytics  
Podsakoff, P. M., MacKenzie, S. B., Lee, J. Y., & Podsakoff, N. P.
(2003). Common method biases in behavioral research: A critical review
of the literature and recommended remedies. Journal of Applied
Psychology, 88(5), 879–903. https://doi.org/10.1037/0021-9010.88.5.879  
Preacher, K. J., & Hayes, A. F. (2008). Asymptotic and resampling
strategies for assessing and comparing indirect effects in multiple
mediator models. Behavior Research Methods, 40(3), 879–891. https://doi.org/https://doi.org/10.3758/BRM.40.3.879  
Revelle, W. (2022) psych: Procedures for personality and psychological
research. Northwestern University, Evanston, Illinois, USA. https://CRAN.R-project.org/package=psych Version =
2.2.5  
R Core Team (2022). R: A language and environment for statistical
computing. R Foundation for Statistical Computing, Vienna, Austria. https://www.R-project.org/  
Rinker, T. W., & Kurkiewicz, D. (2017). pacman: Package management
for R. version 0.5.0. Buffalo, New York. http://github.com/trinker/pacman Rosseel, Y. (2012).
lavaan: An R package for structural equation modeling. Journal of
Statistical Software, 48(2), 1–36. https://doi.org/10.18637/jss.v048.i02  
Sarkar, D. (2008) Lattice: Multivariate Data Visualization with R.
Springer, New York. ISBN 978-0-387-75968-5  
Satorra, A., & Bentler, P. M. (2001). A scaled difference chi-square
test statistic for moment structure analysis. Psychometrika, 66(4),
507-514.  
Semin, G. R., Higgins, T., de Montes, L. G., Estourget, Y., &
Valencia, J. F. (2005). Linguistic signatures of regulatory focus: How
abstraction fits promotion more than prevention. Journal of Personality
and Social Psychology, 89(1), 36–45. https://doi.org/10.1037/0022-3514.89.1.36  
Simmering, M. J., Fuller, C. M., Richardson, H. A., Ocal, Y., &
Atinc, G. M. (2014). Marker variable choice, reporting, and
interpretation in the detection of common method variance.
Organizational Research Methods, 18(3), 473–511. https://doi.org/10.1177/1094428114560023  
Sobel, M. E. (1982). Asymptotic confidence intervals for indirect
effects in structural equation models. Sociological Methodology, 13,
290–312. https://doi.org/https://doi.org/10.2307/270723  
Stanley, D. (2021). apaTables: Create American Psychological Association
(APA) style tables. R package version 2.0.8. https://CRAN.R-project.org/package=apaTables  
Steiner, M. D., & Grieder, S. G. (2020). EFAtools: An R package with
fast and flexible implementations of exploratory factor analysis tools.
Journal of Open Source Software, 5(53), 2521. https://doi.org/10.21105/joss.02521  
Therneau, T. M. (2022). A package for survival analysis in R. R package
version 3.3-1. https://CRAN.R-project.org/package=survival  
Therneau,T. M., & Grambsch, P. M. (2000). Modeling survival data:
Extending the Cox Model. Springer, New York. ISBN 0-387-98784-3.  
Tierney. N., Cook, D., McBain, M., & Fay, C. (2021). naniar: Data
structures, summaries, and visualisations for missing data. R package
version 0.6.1. https://CRAN.R-project.org/package=naniar Ushey, K.,
Allaire, J., Wickham, H., & Ritchie, G. (2022). rstudioapi: Safely
Access the RStudio API. R package version 0.14. https://CRAN.R-project.org/package=rstudioapi.  
Wei, T., & Simko, V. (2021). R package ‘corrplot’: Visualization of
a correlation matrix (Version 0.92). https://github.com/taiyun/corrplot  
Wickham, H. (2016). ggplot2: Elegant graphics for data analysis.
Springer-Verlag, New York.  
Wickham. H., François, R., Henry, L., & Müller, K. (2022). dplyr: A
grammar of data manipulation. R package version 1.0.9. https://CRAN.R-project.org/package=dplyr  
Wickham, H., Miller, E., & Smith, D. (2022). haven: Import and
export ‘SPSS’, ‘Stata’ and ‘SAS’ Files. R package version 2.5.0. https://CRAN.R-project.org/package=haven  
Xie, Y., Allaire, J. J., & Grolemund, G. (2018). R markdown: The
definitive guide. Chapman and Hall/CRC. https://bookdown.org/yihui/rmarkdown/  
Yuan, K.-H., & Bentler, P. M. (2000). 5. Three likelihood-based
methods for mean and covariance structure analysis with nonnormal
missing data. Sociological Methodology, 30(1), 165–200. https://doi.org/https://doi.org/10.1111/0081-1750.00078  
Zeileis, A., & Croissant, Y. (2010). Extended model formulas in R:
Multiple parts and multiple responses. Journal of Statistical Software,
34(1), 1–13. doi:10.18637/jss.v034.i01. https://doi.org/10.18637/jss.v034.i01  
Zeileis, A., & Hothorn, T. (2002). Diagnostic checking in regression
relationships. R News 2(3), 7–10. https://CRAN.R-project.org/doc/Rnews/ Zhao, X., Lynch,
J. G., Jr., & Chen, Q. (2010). Reconsidering Baron and Kenny: Myths
and truths about mediation analysis. Journal of Consumer Research,
37(2), 197–206. https://doi.org/https://doi.org/10.1086/651257
